# Distribution Patterns of Polyphosphate Metabolism Pathway and Its Relationships With Bacterial Durability and Virulence

**DOI:** 10.3389/fmicb.2018.00782

**Published:** 2018-04-24

**Authors:** Liang Wang, Jiawei Yan, Michael J. Wise, Qinghua Liu, James Asenso, Yue Huang, Shiyun Dai, Zhanzhong Liu, Yan Du, Daoquan Tang

**Affiliations:** ^1^Department of Bioinformatics, School of Medical Informatics, Xuzhou Medical University, Xuzhou, China; ^2^Xuzhou Infectious Diseases Hospital, Xuzhou, China; ^3^School of Computer Science and Software Engineering, University of Western Australia, Perth, WA, Australia; ^4^The Marshall Centre for Infectious Diseases Research and Training, University of Western Australia, Perth, WA, Australia; ^5^Jiangsu Key Laboratory of New Drug Research and Clinical Pharmacy, School of Pharmacy, Xuzhou Medical University, Xuzhou, China; ^6^School of Anesthesiology, Xuzhou Medical University, Xuzhou, China; ^7^Center for Experimental Animals, Xuzhou Medical University, Xuzhou, China

**Keywords:** polyphosphate, virulence, durability, proteome, lifestyle, hidden Markov model, phylogenetics, sit-and-wait hypothesis

## Abstract

Inorganic polyphosphate (polyP) is a linear polymer of orthophosphate residues. It is reported to be present in all life forms. Experimental studies showed that polyP plays important roles in bacterial durability and virulence. Here we investigated the relationships of polyP with bacterial durability and virulence theoretically. Bacterial lifestyle, environmental persistence, virulence factors (VFs), and species evolution are all included in the analysis. The presence of seven genes involved in polyP metabolism (*ppk1*, *ppk2*, *pap*, *surE*, *gppA*, *ppnK*, and *ppgK*) and 2595 core VFs were verified in 944 bacterial reference proteomes for distribution patterns via HMMER. Proteome size and VFs were compared in terms of gain and loss of polyP pathway. Literature mining and phylogenetic analysis were recruited to support the study. Our analyzes revealed that the presence of polyP metabolism is positively correlated with bacterial proteome size and the number of virulence genes. A potential relationship of polyP in bacterial lifestyle and environmental durability is suggested. Evolutionary analysis shows that polyP genes are randomly lost along the phylogenetic tree. In sum, based on our theoretical analysis, we confirmed that bacteria with polyP metabolism are associated with high environmental durability and more VFs.

## Introduction

Inorganic polyphosphates (polyP) are homogenous polymers consisting of orthophosphate residues linked by phosphoanhydride bonds (Brown and Kornberg, 2008; Rao et al., 2009; Albi and Serrano, 2016). polyP can be simply synthesized through dehydration of orthophosphates at elevated temperature and it is ubiquitous in environment, especially in extreme places like volcanoes and deep oceans (Achbergerova and Nahalka, 2011). Thus, polyP has been predicted to be present in prebiotic times as a polyanionic scaffold for macromolecular assembly (Brown and Kornberg, 2004), although different opinions exist (Keefe and Miller, 1995). A recent study confirmed that polyP with bacterial origin plays an essential role in oligotrophic microbial ecosystem in coral reefs and may contribute to the phosphorus cycle in Earth’s early history (Zhang et al., 2015).

First discovered by Arthur Meyer in microorganisms and named volutin due to its pink color stained by blue dyes in 1904, the substance was further identified as polyP in 1947 by J. M. Wiame (Kornberg, 1995). Since its discovery, polyP metabolism has been experimentally linked to a variety of functions in prokaryotes, such as replication, survival, and virulence, etc., because of its known connections with more than 500 adenosine triphosphate (ATP) reactions (Brown and Kornberg, 2008; Achbergerova and Nahalka, 2011; Moreno and Docampo, 2013; Albi and Serrano, 2016). polyP has been claimed to be present in all cells in nature and serves as a rich energy and phosphate source (Zhang et al., 2002; Brown and Kornberg, 2008; Rao et al., 2009; Achbergerova and Nahalka, 2011). Due to this widely accepted belief, few studies focused on polyP deficient bacteria. Although polyP has been identified and quantitatively measured in mammalian cells, the corresponding enzymes for polyP synthesis and degradation are still elusive and the functions of polyP in eukaryotes are not well defined (Pavlov et al., 2010; Moreno and Docampo, 2013). The only prokaryotic polyP enzyme with eukaryotic homologs is polyphosphate kinase 1 (PPK1), which is found in the yeast *Candida humicola* (34% similarity with *Helicobacter pylori* PPK1) and in the soil amoeba *Dictyostelium discoideum* (30% similarity with *Escherichia coli* PPK1) (Brown and Kornberg, 2004, 2008; Rao et al., 2009; Moreno and Docampo, 2013). Due to the rare incidence of polyP in eukaryotes, it is proposed that the eukaryotic homolog of PPK1 actually originated from prokaryotes (Rao et al., 2009). On the other hand, lack of homologies between eukaryotic and prokaryotic enzymes for polyP metabolism can serve as potential targets for drug development (Brown and Kornberg, 2004; Singh et al., 2016).

**FIGURE 1:**
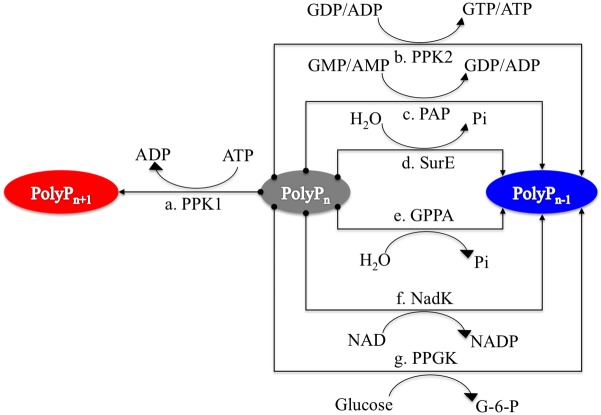
Schematic illustration of seven enzymes involved in polyphosphate synthesis and utilization in prokaryotes. PPK1 represents the synthesis enzyme for polyP metabolism while other six enzymes, PPK2, PAP, SurE, GPPA, NadK, and PPGK, have preferences for polyP degradation although the reactions are reversible. Synthesis pathway is linked to red oval while degradation pathways have linkages with blue oval.

Bacterial polyP metabolism involves two steps, that is, synthesis and degradation (**Figure 1**). A total of seven enzymes (structural genes) are directly linked with these processes (Rao et al., 2009), which are: PPK1 (Ahn and Kornberg, 1990; Akiyama et al., 1992), polyphosphate kinase 2 (PPK2; Zhang et al., 2002), polyphosphate:AMP phosphotransferase (PAP; Rao et al., 2009), 5′/3′-nucleotidase (SurE; Proudfoot et al., 2004), exopolyphosphatase (PPX; Choi et al., 2012), NAD kinase (NadK) (Nakamichi et al., 2013), and polyphosphate glucokinase (PPGK; Hsieh et al., 1996). 1,3-Diphosphoglycerate-polyphosphate phosphotransferase (EC 2.7.4.17) is also reported to facilitate the formation of polyP in bacteria by using 3-phospho-D-glycerol-phosphate as the phosphate donor. However, the enzyme will not be considered in this study because it has never been purified and studied experimentally (Kulaev et al., 2004). For details, please refer to **Table 1**.

**Table 1:** Representative reactions directly involved in bacterial polyphosphate metabolism pathways.

Bacteria	Gene	Enzyme	L^#^	UPID^#^	PID^#^	PN^#^	EC	Reaction
*Escherichia coli* K12	*ppk1*	Polyphosphate kinase 1 (PPK1)	687	P0A7B1	PF02503	PP_kinase	2.7.4.1	(polyP)*_n_* + ATP ↔ (polyP)*_n_*_+__1_ + ADP
*Pseudomonas aeruginosa* PAO1	*ppk2*	Polyphosphate kinase 2 (PPK2)	304	Q9I154	PF03976	PPK2	/	(polyP)*_n_* + GDP/ADP ↔ (polyP)*_n_*_-__1_ + GTP/ATP
*Pseudomonas aeruginosa* PAO1	*pap*	Polyphosphate:AMP phosphotransferase (PAP)	496	Q9HYF1	PF03976	PPK2	/	(polyP)*_n_* + GMP/AMP ↔ (polyP)*_n_*_-__1_ + GDP/ADP
*Escherichia coli* K12	*surE*	5′/3′-Nucleotidase (SurE)	292	P0A840	PF01975	SurE	3.1.3.2	(polyP)*_n_* + H_2_O ↔ (polyP)*_n_*_-1_ + P_i_
*Escherichia coli* K12	*gppA*	Exopolyphosphatase (PPX)	513	P0AFL6	PF02541	Ppx-GppA	3.6.1.11	(polyP)*_n_* + H_2_O ↔ (polyP)*_n_*_-__1_ + P_i_ pppGpp ↔ ppGpp + P_i_
*Mycobacterium tuberculosis* H37Rv	*ppnK*	NAD kinase	306	P9WHV7	PF01513	NAD_kinase	2.7.1.23	(polyP)*_n_* + NAD ↔ (polyP)*_n_*_-__1_ + NADP
*Mycobacterium tuberculosis* H37Rv	*ppgK*	Polyphosphate glucokinase	265	P9WIN1	PF00480	ROK	2.7.1.63	(polyP)*_n_* + glucose ↔ (polyP)*_n_*_-__1_ + glucose-6-P
*Dictyostelium discoideum*	*arpABCEFGH^∗^*	Actin-related protein	/	/	PF00022	Actin	/	Arp(G) +*n*ATP ↔ Arp(F) + (polyP)*n* + *n*ADP


*^#^L, enzyme length; UPID, UniProt ID; PID, Pfam ID; PN, Pfam name. ^∗^*arpABCEFGH* is a set of actin-related genes and their corresponding protein products form a complex and are suspected to be an unknown mechanism for polyP synthesis in bacteria.*

Polyphosphate is one of the five energy storage compounds identified in bacteria according to the principles proposed by Wilkinson (1963) and Wang and Wise (2011). So far, its metabolism has been linked with bacterial durability and virulence phenotypes based on limited experimental evidence (Rashid et al., 2000b; Kim et al., 2002; Jahid et al., 2006; Seufferheld et al., 2008). Durability and virulence of major pathogens *Shigella* spp., *Salmonella* spp., *Mycobacterium tuberculosis*, *Pseudomonas aeruginosa*, *Vibrio cholerae*, and *Francisella tularensis*, etc. are all compromised when there are mutations of polyP metabolism related genes, especially the principal gene *ppk1* for polyP synthesis (Rashid et al., 2000b; Kim et al., 2002; Jahid et al., 2006; Choi et al., 2012; Chuang et al., 2013; Nikel et al., 2013). In addition, mobility is an essential mechanism for a majority of pathogens in order to invade and establish infections in hosts. Motility is impaired in six *ppk1*-mutated pathogens, which further proves the roles of polyP in bacterial pathogenesis (Rashid et al., 2000a). In contrast, a mutated *Aerobacter aerogenes* without inorganic polyP accumulation is reported to have no obvious physiological disabilities (Harold and Harold, 1963). However, physiological impacts seen in experimental studies cannot provide a systematic support for the roles of polyP in bacterial environmental persistence and pathogenicity. In addition, because of the assumed ubiquity of polyP across organisms, there is no study examining whether polyP-free bacteria exist (Albi and Serrano, 2016). Several studies have used BLAST to search for the presence of principal polyP genes, *ppk1* and *ppk2*, in microorganisms (Zhang et al., 2002; Rao et al., 2009; Whitehead et al., 2014). Zhang et al. (2002) noticed a group of microorganisms lacking of both *ppk1* and *ppk2* genes, most of which belong to the domain of Archaea. Of the 27 bacteria without *ppk1* and *ppk2*, some of them turn out to be vector-borne or obligate intracellular pathogens, such as *Buchnera aphidicola*, *Rickettsia prowazekii*, and *Ureaplasma urealyticum*, etc. (Zhang et al., 2002). Rao et al. (2009) also summarized the distribution of *ppk1* and *ppk2* in a set of bacteria without mentioning those lacking both of the two genes. In order to explain why *ppk1* and *ppk2*-deficient bacteria exist, after a complete search of available complete genome, Whitehead et al. (2014) proposed that current knowledge may be limited and unknown mechanisms exist for polyP synthesis. However, this suggestion is still based on the presumption that polyP is present in all organisms.

To theoretically examine the potential relationship of polyP with bacterial durability and virulence, we investigated the issue from several aspects: host–pathogen interactions, environmental persistence, proteome size, virulence factors (VFs), and phylogenetics, etc., together with polyP metabolism pathway distribution. Hidden Markov models (HMMs) were used in order to search for homologous sequences of polyP metabolism related enzymes, thoroughly. As for VFs, BLAST was recruited to explore virulence genes in studied bacterial proteomes. We confirmed the concurrent relationship between *ppk1* and *ppk2*, as previously proposed (Whitehead et al., 2014). In addition, the lifestyle of bacteria with full polyP pathway tend to be more diverse and more durable in the environment, with a majority of bacteria dwelling in soil or marine environments and adopting a free-living lifestyle, while those completely losing polyP metabolism are heavily host-dependent with obligate intracellular or symbiotic lifestyle. Our study also revealed that polyP metabolism is positively related to the number of VFs. However, having a large number of VFs does not necessarily mean high virulence in a bacterium; it might also indicate potential for it to be an emerging pathogen. Finally, phylogenetic analysis identified a random loss pattern for polyP genes in bacteria. In sum, our study systematically investigated the relationships of polyP with bacterial durability and virulence through bioinformatic analysis, which provided insights into bacterial polyP metabolism. The study can serve as theoretical basis for further experimental exploration of the relationships between bacterial durability and virulence based on the sit-and-wait hypothesis (Wang et al., 2017).

## Materials and Methods

### Collection of Bacterial Proteomes

Bacterial proteomes were downloaded from UniProt (The UniProt Consortium, 2017) in February 2016 by using filters of Bacteria and Reference Proteomes. A total of 3442 bacterial proteomes were collected. Unclassified or inappropriately named bacteria were all excluded from the collection, such as Acetobacteraceae bacterium, *Candidatus*, secondary endosymbiont of *Ctenarytaina eucalypti*, bacteria symbiont, and SAR116 cluster alpha proteobacterium HIMB100, etc. Only those with names in compliance with classical binominal nomenclature were collected. Proteome size of *Anoxybacillus flavithermus* (taxid: 33934) did not match updated UniProt data, so was also excluded. In addition, considering the skewed distribution of some bacterial species in different genera, only one representative bacterium was allowed from each genus in order to make the analysis less biased. A final set of 944 bacterial proteomes was used for the bioinformatics analysis in this study. A complete list of all the bacteria with their polyP pathway distributions is available in the Supplementary Table S1.

### Homologs of polyP Metabolism Enzymes

Enzymes directly linked with polyP metabolism in bacteria were first collected through a comprehensive up-to-date review of literature (Zhang et al., 2002; Brown and Kornberg, 2008; Albi and Serrano, 2016). Integrative information for the seven enzymes such as gene name, protein length, and identifier number was sourced from highly reliable online databases such as UniProt and Pfam, etc., and can be found in **Table 1** (Finn et al., 2010; The UniProt Consortium, 2017). The actin-related protein complex is currently only found in eukaryote *D. discoideum* and assumed to be an unidentified polyP synthesis enzyme in bacteria due to its distant homology (Whitehead et al., 2014). All seven actin-related proteins, ArpA, ArpB, ArpC, ArpE, ArpF, ArpG, and ArpH, share the same actin domain according to the Pfam sequence analysis. Thus, its distribution among bacteria is also included in Supplementary Table S1 just for reference. Since PPK2 and PAP share the same HMM, we combine the two enzymes together as a single search. HMMs of all enzymes were constructed using HMMER, based on multiple sequence alignments downloaded directly from Pfam database (Eddy, 2003; Finn et al., 2010). The command hmmsearch from the HMMER suite was used to search all proteomes for homologous sequences of the listed enzymes in **Table 1** with e-value set to 1e-50. Since the *arp* genes are only distantly related to corresponding sequences in bacteria, an e-value of 1e-10 was used for that search. The results were recorded for further analysis, and are here found in Supplementary Table S1. Short Python scripts (Cock et al., 2009) were written to perform file handling and text mining in order to integrate data from hmmsearch results. Based on the distribution of polyP pathway metabolism enzymes, bacteria were divided into six groups: PPK1_2, No_PPK1_2, PPK1, PPK2, Full_Path, and No_Path. PPK1_2 represents those bacteria with both PPK1 and PPK2 enzymes. No_PPK1_PPK2 means bacteria without PPK1 and PPK2. For the PPK1 group, bacteria must have PPK1 but no PPK2 while all other enzymes are optional. The same rule applies to PPK2 group. For Full_Path, all related enzymes must be present in the proteome while No_Path means no polyP metabolism enzymes were detected in the group of bacteria.

### Homologs of Bacterial VFs

The VF sequence package includes 2595 non-redundant core bacterial VFs sourced from VFDB (downloadable from VFDB website^1^; Chen et al., 2005). The phmmer command from HMMER package was used to search for VF homologs in proteomes using an e-value of 1e-50. Results were all further processed for text mining through a set of short python scripts in order to calculate the number of homologous VFs in each bacteria (Cock et al., 2009). VF numbers were then used for comparative analysis by correlating with polyP metabolism distribution.

### Data Mining of Bacterial Lifestyle and Related Phenotypes

Proteomes with complete gain or loss of polyP pathway were further investigated. Genome metadata from Pathosystems Resource Integration Center (PATRIC) were explored through data mining methods for bacterial lifestyle and pathogenicity wherever needed (Wattam et al., 2017). For each bacterium, related literature was manually reviewed for extracting relevant information. For detailed results, please refer to Supplementary Table S2 for bacteria with polyP and Supplementary Table S3 for bacteria without polyP.

### Phylogenetic Analysis

NCBI taxonomy identifiers were mined in the genome metadata sourced from PATRIC through text matching of proteome IDs and organism IDs, respectively, which were later used for constructing a phylogenetic tree via phyloT^2^ in order to investigate distribution patterns of polyP-gain and polyP-loss bacteria in evolutionary settings (Letunic and Bork, 2007; Wattam et al., 2014). phyloT uses NCBI taxonomy identifier to generate a pruned tree automatically in user-defined output format. Complete clades can be simply included, with interruption at desired taxonomic levels and with optional filtering of unwanted nodes. Tree visualization and annotation were undertaken through the online editing tool iTOL with pre-defined tol_binary and tol_simple_bar templates, etc. (Letunic and Bork, 2016).

### Statistical Analysis and Data Visualization

Python scripts and R programming, especially the ggplot2 package, were used throughout the study for data visualization (R Core Team, 2015). Unpaired two-tailed Student’s *t*-test was used for statistical analysis. Significant difference was defined as *p*-value < 0.05.

## Results and Discussion

A set of completely annotated bacterial reference proteomes belonging to 944 bacterial genera was analyzed from four aspects in this study through HMM- and BLAST-based homologous sequence searching. These were: the distribution of polyP pathways, the number of VFs, gain or loss of polyP in associated with bacterial lifestyle and durability, and the evolution of polyP metabolism. Since bacterial proteome size can be a reflection of bacterial lifestyle to some degree (Moran, 2002; McCutcheon and Moran, 2011), we incorporated this factor into the analysis.

### Enzymes Involved in polyP Metabolism

First isolated from *E. coli*, PPK1 is a major polyP synthetic enzyme that is highly conserved and widely distributed in bacteria including numerous pathogens (Zhang et al., 2002; Brown and Kornberg, 2004). It reversibly catalyses elongation of polyP chains by transferring phosphate from the terminal of ATP, though the reaction favors synthesis (Rao et al., 2009). Pathogens such as *M. tuberculosis* and *P. aeruginosa*, etc., lacking *ppk1* are referred to as PPK1-null mutants and still have certain level of polyP accumulation (Brown and Kornberg, 2004; Rao et al., 2009), which led to the identification of PPK2 (Zhang et al., 2002). Unlike PPK1 favoring the synthesis reaction, PPK2 prefers a polyP driven synthesis of GTP and ATP from GDP and ADP (Brown and Kornberg, 2004). Analysis of PPK1 and PPK2 homologs among microorganisms showed that the PPK1 only genotype is present in 60% taxa and less than half of taxa have PPK1 and PPK2 together (Whitehead et al., 2014). In addition, PPK2 is rarely found alone (Zhang et al., 2002; Whitehead et al., 2014). In fact, through BLAST searching of bacterial genomes, a significant number of bacteria do not have either PPK1 or PPK2 (Zhang et al., 2002; Whitehead et al., 2014). This could be due to the limitation of BLAST search for distant homological sequence identification (Skewes-Cox et al., 2014) or due to unknown mechanisms existing in bacteria for polyP synthesis (Whitehead et al., 2014). For example, an Arp complex (DdPPK2) similar to actin-related proteins in DdPPK1-deficient slime mold *D. discoideum* is found to be responsible for polyP synthesis (Gomez-Garcia and Kornberg, 2004) and has distant homologs in bacteria (Supplementary Table S1; Whitehead et al., 2014). Thus, there is a need to confirm the existence of alternative polyP synthesis enzymes. Otherwise, for reasons that require further investigation, some bacterial species appear not to need polyP.

Polyphosphate:AMP phosphotransferase is a degradative enzyme of polyP, and a homolog of PPK2, which was initially purified from *Acinetobacter* strain 210A (Rao et al., 2009). It catalyses the formation of ADP and ATP from AMP by utilizing polyP, and was first found in *Corynebacterium xerosis* (Kulaev et al., 2004). Profile HMM analysis shows that PPK2 and PAP belong to the same family (Finn et al., 2010). One of the most important polyP catabolism enzymes is PPX. Its main function is to progressively break phosphoanhydride bonds of polyP through hydrolysis (Achbergerova and Nahalka, 2011). There are two PPXs in bacteria: PPX1 encoded by *ppx* and PPX2 encoded by *gppA* (Rao et al., 2009). The two enzymes show great similarity and are grouped by profile HMM analysis into the same family with a single Ppx-GppA domain (Finn et al., 2010). Notably, *ppx* normally forms an operon with *ppk* in bacterial genomes (Akiyama et al., 1993). In addition to polyP hydrolysis, GPPA also breaks down guanosine pentaphosphate (pppGpp), an important second messenger, into ppGpp in bacteria, which links polyP metabolism with bacterial stringent responses (Kulaev et al., 2004). However, this does not exclude the existence of other enzymes with PPX activities. For example, the nucleotidase SurE was reported to hydrolyze short-chain polyP (Proudfoot et al., 2004). It is noteworthy that no endopolyphosphatase has ever been discovered in prokaryotes (Kulaev et al., 2004). As for NadK, it is present in both eukaryotes and prokaryotes with its main function of using polyP as a phosphate donor for the phosphorylation of NAD to NADP. Finally, PPGK catalyses the formation of glucose-6-phosphate by using polyP as phosphate donor (Rao et al., 2009). This reaction is evolutionarily important since it is the first support for polyP as an energy and phosphate donor that is independent of nucleoside phosphate system (Kulaev et al., 2004). **Table 1** summarizes the polyP metabolism in bacteria.

### Distribution of Directly Related polyP Enzymes

**FIGURE 2:**
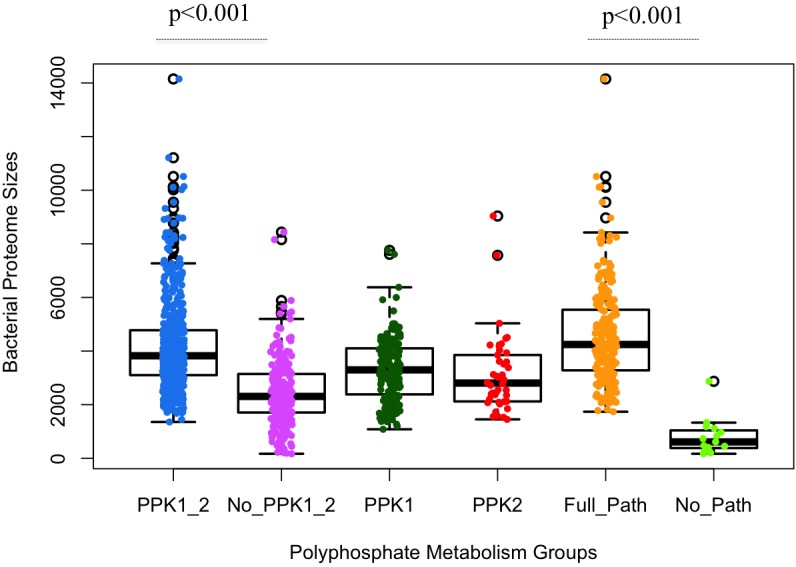
Statistical analysis of polyphosphate metabolism abilities and bacterial proteome sizes via boxplots overlaid with dots. *X*-axis shows the six previously described polyP groups. *Y*-axis indicates the number of proteins in a proteome. Each circled dot represents a single bacterium. Proteome sizes of PPK1_2/No_PPK1_2 and Full_Path/No_Path show statistically significant differences between each other, respectively. Unpaired two-tailed Student’s *t*-test was used (*p*-value < 0.001).

Through HMM-based search for homologous sequences of polyP metabolism enzymes, we obtained a map of the distribution of polyP metabolism pathway in our database of bacterial proteomes (Supplementary Table S1). Significant differences in proteome size were observed among different groups of bacteria, versus gain and loss of polyP metabolism enzymes (**Figure 2**). Initial analysis confirms that the two major polyP metabolism enzymes PPK1 and PPK2 are widely conserved in bacteria, respectively (Zhang et al., 2002; Brown and Kornberg, 2004, 2008). The number of bacteria in the six polyP groups, that is PPK1_2, No_PPK1_2, PPK1, PPK2, Full_Path, and No_Path, were 504, 234, 165, 42, 214, and 15, respectively. PPK1_2 had the highest amount of bacteria while near one-fourth of the studied bacterial genera had neither PPK1 nor PPK2. Correlation analysis via two-sample unequal variance Student’s *t*-test showed that the difference between PPK1_2 and No_PPK1_2 in terms of bacterial proteome size was significant (*p* < 0.001; **Figure 2**). Thus, concurrence of *ppk1* and *ppk2* in polyP metabolism is statistically correlated with larger bacterial proteome size, although the two genes do not normally form an operon. In contrast, the bacteria without both ppk1 and ppk2 tend to have smaller proteomes. Further analysis showed that *ppk1* and *ppx* are the most abundant pair of genes co-existing in studied proteomes (627 bacterial genera). However, this is not surprising since the two genes normally form operon in genome (Akiyama et al., 1993) and will not be considered in this study. PPK1 or PPK2 only groups have fewer species than the set with both enzymes, and the PPK2 only category contains 42 out of 944 genera, which supports the idea that PPK1 and PPK2 are more likely present in the genome concurrently and may have strong links in metabolic network within bacteria while PPK2 is rarely found alone (Whitehead et al., 2014). Finally, the No_Path group has only 15 bacteria, which indicates that the chance for bacteria to lose both polyP synthesis and degradation abilities is very rare, and accords well with previous discovery that polyP metabolism is widely spread in organisms (Brown and Kornberg, 2004, 2008; Kulaev et al., 2004; Rao et al., 2009). However, it also confirms that not all bacteria accumulate and utilize polyP as an energy mechanism, which is worth of further investigation.

### Relationships Between polyP Metabolism and Bacterial Pathogenicity

**FIGURE 3:**
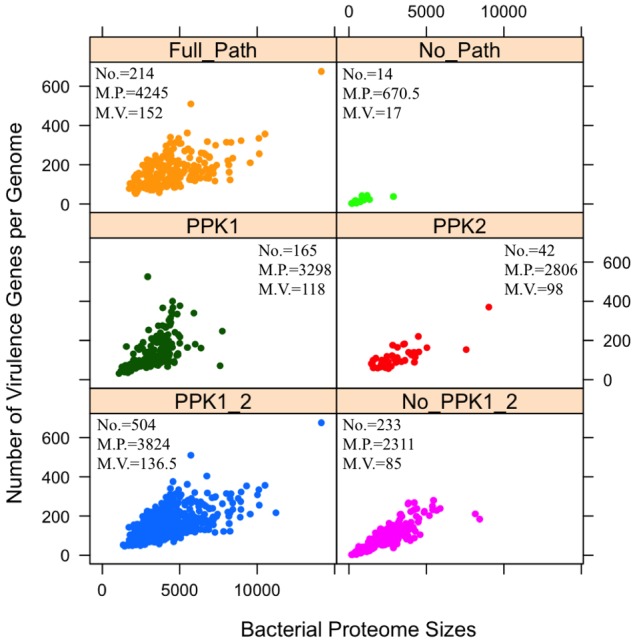
Analysis of distribution patterns of bacterial virulence factors in terms of polyphosphate metabolism via scattered dot plots. Each sub-graph represents one of the six previously described polyP groups. For each group, numbers of bacterial species (No.), median number of proteome sizes (M.P.), and median number of virulence factors (M.V.) are presented. *Y*-axis indicates the number of virulence factors in a proteome. Each circled dot represents a single bacterium. Unpaired two-tailed Student’s *t*-test shows significant differences in two groups: PPK1_2/No_PPK1_2 and Full_Path/No_Path, in terms of the number of virulence factors (*p* < 0.001).

The sit-and-wait hypothesis predicts that bacterial durability is positively correlated with virulence (Walther and Ewald, 2004; Wang et al., 2017). Since polyP is experimentally proven to contribute to bacterial persistence (Rashid et al., 2000b; Kim et al., 2002; Jahid et al., 2006; Brown and Kornberg, 2008; Rao et al., 2009; Albi and Serrano, 2016; Peng et al., 2016) and plays an essential role in bacterial durability as an energy storage mechanism (Achbergerova and Nahalka, 2011; Wang and Wise, 2011), we postulate that bacteria with polyP metabolism pathway might have more virulence genes and *vice versa*. Thus, we investigated whether polyP metabolism gain or loss could have a relationship with the number of bacterial virulence genes. The correlation analysis is shown in the form of scattered dots representing the number of virulence genes corresponding to bacterial proteome sizes (**Figure 3**).

A positive relationship between polyP metabolism and the number of VFs has been observed in **Figure 3** through statistical analysis. By comparing the PPK1_2 and No_PPK1_2 groups, a significant difference in the number of virulence genes exists (unpaired two-tailed Student’s *t*-test, *p* < 0.001). Bacteria with both PPK1 and PPK2 enzymes tend to have more virulence genes while those without PPK1 and PPK2 have fewer virulence genes, which indicate that pathogens with polyP metabolism might be more virulent or have the potential to cause diseases as emerging pathogens. This result sheds a light on the linkage between durability and virulence, which might theoretically support the sit-and-wait hypothesis (Walther and Ewald, 2004). Comparison of Full_Path and No_Path groups is the extreme situation of comparing PPK1_2 and No_PPK1_2 groups, where the gap between the two groups is larger with statistical significant in terms of number of virulence genes (unpaired two-tailed Student’s *t*-test, *p* < 0.001). In fact, more studies are linking bacterial energy storage metabolism with bacterial virulence (Zevenhuizen and Ebbink, 1974; Wang and Bakken, 1998; Rashid et al., 2000b; Kim et al., 2002; Bourassa and Camilli, 2009; Lerner et al., 2009; Rao et al., 2009; Finkelstein et al., 2010; Chandra et al., 2011; Sirakova et al., 2012; Moreno and Docampo, 2013; Peng et al., 2016). Those without energy storage compounds tend to be mildly virulent or not virulent at all (Bourassa and Camilli, 2009; Peng et al., 2016; Wang et al., 2017). Thus, polyP may serve as a good candidate for testing the sit-and-wait hypothesis experimentally. In addition, by comparing No_PPK1_2 and No_Path groups using Student’s *t*-test (*p* < 0.001) in terms of the number of VFs, significant difference is observed, which shed lights on how the presence of such enzymes [e.g., PAP, SurE, guanosine pentaphosphate phosphohydrolase/exopolyphosphatase (GPPA/PPX), NadK, PPGK] can contribute to higher virulence, etc. In fact, some of the enzymes have already been linked with bacterial virulence through experimental studies. For example, GPPA/PPX has been extensively studied in pathogenic bacteria, such as *E. coli*, *Campylobacter jejuni*, *P. aeruginosa*, *Bacillus cereus*, and *M. tuberculosis*, through which *ppx* gene was linked with a variety of pathogenic phenotypes like viability, biofilm formation, type III secretion system, sporulation and swarming mobility, etc. (Rivas et al., 2003; He et al., 2014; Groisillier et al., 2015). As for other enzymes, it may be worth further investigation for their roles in pathogenesis.

### polyP Contributes to Diverse Lifestyle and Environmental Persistence

There is a wide distribution of bacterial proteome sizes in this study. It is clear that these bacteria are at different evolutionary stages from obligate intracellular to free-living bacteria (Henrissat et al., 2002). As for the 14 bacteria without any polyP metabolism enzymes, the average proteome size is 818 amino acids. All of them are (endo)-symbionts, obligate intracellular pathogens, or host-associated/vector-borne parasites (Supplementary Table S3; Nelson and Stegen, 2015). According to the genome reduction theory, bacteria experiencing genome minimization have a deletion bias toward gene loss and energy metabolism genes are normally eliminated in bacteria such as *Rickettsia* species, *Mycoplasma* species, and *Buchnera* species, etc., due to their exclusive dependence on hosts for energy (Moran, 2002). Thus, loss of polyP metabolism is possibly linked with bacterial parasitic or symbiotic lifestyle, a situation similar to glycogen metabolism (Henrissat et al., 2002). In contrast, proteome size of Full_Path group ranges from 1737 (*Thermanaerovibrio acidaminovorans*) to 14,150 (*Mumia flava*) with an average of 4580, which includes bacteria with a diversity of lifestyles, such as extremophiles, free-living bacteria, or less commonly, host-associated pathogens, etc. (Supplementary Table S2). Most of the bacteria have a preference for environmental living, dwelling in soil or water. Occasionally reported human pathogens in this group emphasize their potential as emerging pathogens, e.g., *Bacteroides thetaiotaomicron* for gastro-intestinal tract infection and *Dysgonomonas gadei* for gall bladder infection (Mishra and Imlay, 2013; Vaughan and Forbes, 2014). Proteome sizes between Full_Path and No_Path groups are significantly different through two-sample unequal variance *t*-test analysis (*p* < 0.001), the result of which strengthens the positive correlation between polyP metabolism and durable bacterial lifestyle.

### Evolution of polyP Metabolism in Bacteria

**FIGURE 4:**
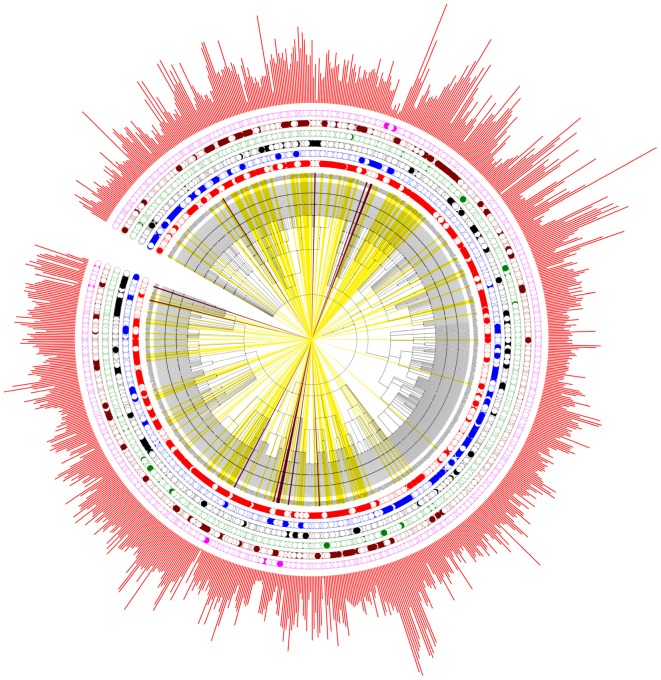
Phylogenetic analysis of the six artificially divided bacterial groups based on gain or loss of polyP-related enzymes. PPK1_2 (red dots), No_PPK1_2 (blue dots), PPK1 (black dots), PPK2 (green dots), Full_Path (brown dots), and No_Path (pink dots). If a bacterium fall into any one of the groups, a dot is marked at the corresponding circled layer. The outmost layer represents bacterial proteome sizes (red bars). In addition, bacteria in Full_Path group are marked by yellow strips while those belonging to No_Path group are colored by dark brown strips.

In order to get an overview of the polyP metabolism in bacteria, we also explored the distribution patterns of different bacterial groups in evolutionary settings. An NCBI taxonomy identifier based phylogenetic tree was constructed via phyloT for the 944 genera. The six previously defined bacterial groups, together with bacterial proteome sizes, are incorporated into the tree (**Figure 4**). PPK1 and PPK2 are the two key enzymes in bacterial polyP metabolism. Of the 944 bacterial species, 669 and 546 have PPK1 and PPK2 enzymes, respectively (Supplementary Table S1). Although comparatively few bacteria have PPK2 only, the enzyme is actually older than PPK1, which was recently confirmed by a phylogenetic study (Achbergerová and Nahálka, 2014). The conclusion supports the hypothesis that bacteria were first capable of utilizing environmental polyP and then developed the ability to synthesize polyP. In this study, all the six groups of bacteria spread along the phylogenetic tree with no apparently clustered patterns, which indicates that polyP metabolism is widely distributed in bacteria and loss of polyP-related genes might be niche-dependent and due to the selective pressure of a particular environment, rather than biological traits or taxonomic relatedness of species. In order to understand how bacteria lose polyP-related enzymes, a more subtle study with the assistance of experiments will be required.

## Conclusion and Future Perspectives

The sit-and-wait hypothesis predicts that pathogenic bacteria with longer external survival time tend to evolve toward higher virulence (Walther and Ewald, 2004; Wang et al., 2017). Thus, the correlation of durability and virulence phenotypes should be reflected at the genomic level. polyP has long been considered as a widespread energy storage mechanism in bacteria, contributing to bacterial persistence in hash conditions (Brown and Kornberg, 2008; Rao et al., 2009; Chuang et al., 2013; Moreno and Docampo, 2013; Nikel et al., 2013; Albi and Serrano, 2016). In this study, we thoroughly searched the distribution of polyP metabolism related enzymes in 944 bacterial genera. According to our study, gain of key polyP metabolism enzymes, PPK1 and PPK2, is significantly linked to higher numbers of VFs. This conclusion connects bacterial durability with virulence and provides a preliminary support for the sit-and-wait hypothesis. In addition, bacteria with polyP metabolism pathway are associated with comparatively larger proteome size, which reflects their complex life cycle while those without polyP metabolism are exclusively host-associated organisms. The result matches well with a previous study, which examined the distribution of glycogen metabolism enzymes in bacteria (Henrissat et al., 2002) and indicates that gain or loss of energy storage mechanism might be a potential indicator of host–bacteria relationship. Literature mining of bacterial lifestyles for 214 Full_Path and 14 No_Path bacteria validated the observation that bacteria with polyP pathway may have a more durable lifestyle, while phylogenetic study showed that polyP metabolism is widespread across bacterial genera (**Figure 4**). However, no evolutionary relatedness is identified in terms of enzyme loss of polyP enzymes.

Although multiple studies have already shown that polyP deficiency reduces bacterial biofilm formation (Rashid et al., 2000b), survival rate (Rao et al., 2009), and stress resistance (Kim et al., 2002), there is no study on how inactivation of polyP enzymes has an impact on the evolution of bacterial virulence. Serial passaging of pathogens through host–environment–host cycles, such as *P. aeruginosa* with PPK1 and/or PPK2 deletion, will reveal how virulence evolves by comparing with wild-type strains that go through the same cycles. Genome sequencing of the passaged wild-type and mutated pathogens will also reveal the types of genes lost during the serial passages. Such experiments, if matched with the theoretical analysis in this study, will be reliable evidence at molecular level for investigating the role of durability in the evolution of virulence.

## Author Contributions

LW and JY conceived the core idea. LW wrote the draft manuscript. JY, QL, JA, YH, SD, ZL, YD, and DT did data collection and visualization. MW contributed to data comparison and statistical analysis. MW, QL, and JA also contributed to manuscript writing.

## Conflict of Interest Statement

The authors declare that the research was conducted in the absence of any commercial or financial relationships that could be construed as a potential conflict of interest.

## Footnotes

1 http://www.mgc.ac.cn/VFs/

2 http://phylot.biobyte.de
