# Supplementary material for: Distribution Patterns of Polyphosphate Metabolism Pathway and Its Relationships With Bacterial Durability and Virulence
Source: Front Microbiol. 2018 Apr 24;9:782. doi: 10.3389/fmicb.2018.00782 (PMC5932413; doi:10.3389/fmicb.2018.00782)
Supplement: Supplementary file 1 [file Table_1.DOCX]

| **Table S1. Distribution of PolyP metabolism related enzymes and virulence factors in 944 bacterial species (genera)** | | | | | | | | | | | | |
| --- | --- | --- | --- | --- | --- | --- | --- | --- | --- | --- | --- | --- |
|  | | | | | | | | | | | | |
| **Taxonomy ID** | **Genus** | **Species** | **Proteome ID** | **Protein count** | ***ppk1*** | ***ppk2/pap*** | ***surE*** | ***ppx/***  ***gppA*** | ***ppnK*** | ***ppgK*** | ***arp**** | **Virulence** |
| 48 | *Archangium* | *gephyra* | UP000035579 | 10110 | 1 | 1 | 1 | 3 | 1 | 3 | 0 | 335 |
| 451 | *Tatlockia* | *micdadei* | UP000032414 | 2989 | 1 | 0 | 1 | 0 | 1 | 0 | 1 | 253 |
| 575 | *Raoultella* | *planticola* | UP000032530 | 2923 | 1 | 0 | 0 | 0 | 0 | 3 | 0 | 69 |
| 632 | *Yersinia* | *pestis* | UP000000815 | 3909 | 1 | 0 | 1 | 2 | 1 | 5 | 1 | 366 |
| 728 | *Avibacterium* | *paragallinarum* | UP000033614 | 2171 | 0 | 0 | 1 | 1 | 1 | 4 | 1 | 98 |
| 1044 | *Erythrobacter* | *longus* | UP000027647 | 3219 | 1 | 1 | 1 | 2 | 1 | 1 | 0 | 109 |
| 1076 | *Rhodopseudomonas* | *palustris* | UP000032515 | 5580 | 1 | 2 | 1 | 2 | 0 | 0 | 0 | 199 |
| 1140 | *Synechococcus* | *elongatus* | UP000002717 | 2657 | 1 | 1 | 1 | 1 | 1 | 1 | 1 | 89 |
| 1238 | *Piscirickettsia* | *salmonis* | UP000029541 | 2947 | 1 | 0 | 1 | 1 | 1 | 1 | 1 | 141 |
| 1274 | *Dermacoccus* | *nishinomiyaensis* | UP000027986 | 2255 | 1 | 1 | 0 | 2 | 1 | 3 | 1 | 68 |
| 1392 | *Bacillus* | *anthracis* | UP000000594 | 5493 | 1 | 1 | 0 | 1 | 2 | 2 | 1 | 166 |
| 1473 | *Virgibacillus* | *pantothenticus* | UP000036780 | 3923 | 0 | 0 | 1 | 0 | 1 | 5 | 3 | 139 |
| 1661 | *Trueperella* | *pyogenes* | UP000019771 | 1984 | 1 | 0 | 0 | 1 | 1 | 8 | 0 | 65 |
| 2026 | *Thermoactinomyces* | *vulgaris* | UP000037969 | 3820 | 0 | 0 | 0 | 0 | 1 | 3 | 1 | 99 |
| 2045 | *Nocardioides* | *simplex* | UP000030300 | 5426 | 1 | 2 | 0 | 2 | 1 | 6 | 1 | 151 |
| 2340 | *Solemya* | *velum* | UP000030856 | 2657 | 1 | 2 | 1 | 1 | 1 | 0 | 1 | 104 |
| 2754 | *Synergistes* | *jonesii* | UP000027665 | 2481 | 0 | 0 | 1 | 1 | 1 | 1 | 0 | 65 |
| 28885 | *Hydrogenovibrio* | *marinus* | UP000027341 | 2330 | 1 | 1 | 1 | 1 | 1 | 1 | 1 | 147 |
| 31964 | *Clavibacter* | *michiganensis* | UP000001318 | 3058 | 1 | 0 | 0 | 0 | 1 | 5 | 0 | 79 |
| 33033 | *Parvimonas* | *micra* | UP000031386 | 1418 | 0 | 0 | 0 | 0 | 1 | 0 | 0 | 54 |
| 34073 | *Variovorax* | *paradoxus* | UP000035170 | 6788 | 1 | 2 | 1 | 1 | 1 | 4 | 0 | 318 |
| 36849 | *Oxobacter* | *pfennigii* | UP000050326 | 4273 | 1 | 0 | 1 | 3 | 1 | 0 | 4 | 158 |
| 36870 | *Wigglesworthia* | *glossinidia* | UP000000562 | 617 | 0 | 0 | 1 | 0 | 1 | 0 | 1 | 45 |
| 40754 | *Thioploca* | *ingrica* | UP000031623 | 3951 | 1 | 0 | 1 | 1 | 1 | 1 | 0 | 184 |
| 46914 | *Devosia* | *riboflavina* | UP000028981 | 4273 | 1 | 1 | 1 | 1 | 1 | 10 | 0 | 176 |
| 47500 | *Aneurinibacillus* | *migulanus* | UP000037269 | 5649 | 1 | 1 | 1 | 1 | 1 | 2 | 0 | 236 |
| 55969 | *Leucobacter* | *komagatae* | UP000032120 | 2703 | 1 | 2 | 0 | 1 | 0 | 3 | 0 | 103 |
| 56107 | *Cylindrospermum* | *stagnale* | UP000010475 | 6200 | 1 | 1 | 2 | 1 | 1 | 2 | 0 | 190 |
| 56110 | *Oscillatoria* | *acuminata* | UP000010367 | 5755 | 1 | 1 | 2 | 1 | 1 | 1 | 1 | 171 |
| 56780 | *Syntrophus* | *aciditrophicus* | UP000001933 | 3145 | 0 | 0 | 1 | 0 | 1 | 0 | 1 | 142 |
| 59374 | *Fibrobacter* | *succinogenes* | UP000000517 | 2871 | 0 | 0 | 1 | 1 | 1 | 0 | 0 | 72 |
| 59505 | *Actinotignum* | *schaalii* | UP000035032 | 1443 | 1 | 1 | 0 | 1 | 1 | 3 | 0 | 47 |
| 62977 | *Acinetobacter* | *baylyi* | UP000000430 | 3263 | 1 | 1 | 1 | 1 | 1 | 0 | 1 | 150 |
| 63186 | *Zobellia* | *galactanivorans* | UP000008898 | 4708 | 1 | 1 | 1 | 1 | 1 | 3 | 0 | 85 |
| 63737 | *Nostoc* | *punctiforme* | UP000001191 | 6573 | 1 | 2 | 2 | 1 | 1 | 2 | 0 | 258 |
| 68170 | *Lechevalieria* | *aerocolonigenes* | UP000033393 | 8237 | 1 | 1 | 0 | 3 | 2 | 14 | 0 | 216 |
| 69279 | *Aquamicrobium* | *defluvii* | UP000019849 | 4454 | 1 | 1 | 2 | 2 | 1 | 2 | 0 | 173 |
| 71421 | *Haemophilus* | *influenzae* | UP000000579 | 1707 | 0 | 0 | 1 | 1 | 1 | 2 | 0 | 115 |
| 74109 | *Photobacterium* | *profundum* | UP000000593 | 5328 | 1 | 1 | 1 | 2 | 1 | 5 | 1 | 270 |
| 75379 | *Thiomonas* | *intermedia* | UP000002185 | 3134 | 1 | 3 | 1 | 1 | 1 | 1 | 0 | 172 |
| 76114 | *Aromatoleum* | *aromaticum* | UP000006552 | 4483 | 1 | 0 | 1 | 1 | 1 | 0 | 0 | 183 |
| 78245 | *Xanthobacter* | *autotrophicus* | UP000002417 | 4971 | 1 | 4 | 1 | 2 | 1 | 1 | 0 | 204 |
| 80854 | *Moritella* | *viscosa* | UP000032438 | 4219 | 0 | 0 | 1 | 1 | 1 | 2 | 1 | 268 |
| 82374 | *Anaerovibrio* | *lipolyticus* | UP000030993 | 2437 | 0 | 0 | 1 | 1 | 1 | 0 | 1 | 110 |
| 83219 | *Sulfitobacter* | *mediterraneus* | UP000027337 | 3649 | 1 | 2 | 1 | 2 | 0 | 0 | 1 | 116 |
| 83333 | *Escherichia* | *coli* | UP000000625 | 4306 | 1 | 0 | 1 | 2 | 1 | 7 | 1 | 303 |
| 85962 | *Helicobacter* | *pylori* | UP000000429 | 1553 | 1 | 0 | 1 | 1 | 1 | 0 | 1 | 169 |
| 89187 | *Roseovarius* | *nubinhibens* | UP000005954 | 3545 | 1 | 1 | 1 | 2 | 0 | 0 | 1 | 111 |
| 93059 | *Prochlorococcus* | *marinus* | UP000000788 | 1855 | 1 | 0 | 1 | 1 | 1 | 0 | 0 | 44 |
| 96561 | *Desulfococcus* | *oleovorans* | UP000008561 | 3255 | 0 | 0 | 1 | 0 | 1 | 0 | 1 | 127 |
| 99287 | *Salmonella* | *typhimurium* | UP000001014 | 4533 | 1 | 0 | 1 | 2 | 1 | 6 | 1 | 400 |
| 110935 | *Agreia* | *bicolorata* | UP000032503 | 3109 | 1 | 2 | 0 | 0 | 1 | 10 | 0 | 85 |
| 111780 | *Stanieria* | *cyanosphaera* | UP000010473 | 4751 | 1 | 1 | 2 | 1 | 1 | 1 | 0 | 181 |
| 118168 | *Coleofasciculus* | *chthonoplastes* | UP000003835 | 8193 | 1 | 1 | 2 | 1 | 1 | 1 | 0 | 162 |
| 122586 | *Neisseria* | *meningitidis* | UP000000425 | 2001 | 1 | 0 | 1 | 1 | 1 | 0 | 0 | 131 |
| 123214 | *Persephonella* | *marina* | UP000001366 | 2048 | 0 | 0 | 1 | 1 | 1 | 1 | 1 | 106 |
| 138119 | *Desulfitobacterium* | *hafniense* | UP000001946 | 5014 | 1 | 0 | 1 | 2 | 1 | 1 | 0 | 218 |
| 153721 | *Sporocytophaga* | *myxococcoides* | UP000030185 | 5042 | 1 | 1 | 1 | 2 | 1 | 1 | 1 | 136 |
| 154981 | *Aliiroseovarius* | *crassostreae* | UP000050471 | 3465 | 1 | 2 | 1 | 2 | 0 | 0 | 1 | 95 |
| 156889 | *Magnetococcus* | *marinus* | UP000002586 | 3594 | 0 | 2 | 1 | 1 | 1 | 1 | 1 | 183 |
| 157733 | *Anaerobacillus* | *macyae* | UP000035996 | 3973 | 0 | 0 | 0 | 0 | 1 | 5 | 0 | 151 |
| 158190 | *Sphaerochaeta* | *pleomorpha* | UP000005632 | 3150 | 0 | 0 | 1 | 0 | 1 | 6 | 1 | 86 |
| 158822 | *Cedecea* | *neteri* | UP000029516 | 4399 | 1 | 0 | 2 | 2 | 1 | 7 | 1 | 314 |
| 159087 | *Dechloromonas* | *aromatica* | UP000000550 | 4155 | 1 | 2 | 1 | 1 | 1 | 1 | 1 | 250 |
| 160492 | *Xylella* | *fastidiosa* | UP000000812 | 2772 | 1 | 0 | 1 | 1 | 1 | 0 | 1 | 106 |
| 160660 | *Acidihalobacter* | *prosperus* | UP000029273 | 2685 | 1 | 1 | 1 | 0 | 1 | 1 | 0 | 139 |
| 165597 | *Crocosphaera* | *watsonii* | UP000003922 | 5659 | 1 | 2 | 2 | 1 | 1 | 1 | 0 | 130 |
| 167879 | *Colwellia* | *psychrerythraea* | UP000000547 | 4872 | 0 | 0 | 1 | 1 | 1 | 0 | 0 | 225 |
| 169963 | *Listeria* | *monocytogenes* | UP000000817 | 2844 | 0 | 0 | 0 | 0 | 2 | 8 | 2 | 153 |
| 176299 | *Agrobacterium* | *fabrum* | UP000000813 | 5344 | 1 | 1 | 1 | 2 | 0 | 8 | 0 | 257 |
| 177416 | *Francisella* | *tularensis* | UP000001174 | 1528 | 0 | 1 | 0 | 1 | 1 | 2 | 0 | 73 |
| 177437 | *Desulfobacterium* | *autotrophicum* | UP000000442 | 4846 | 0 | 0 | 0 | 0 | 1 | 0 | 1 | 219 |
| 177439 | *Desulfotalea* | *psychrophila* | UP000000602 | 3233 | 1 | 2 | 0 | 0 | 1 | 0 | 1 | 153 |
| 183763 | *Streptomonospora* | *alba* | UP000031675 | 4307 | 1 | 0 | 0 | 1 | 1 | 9 | 1 | 122 |
| 186479 | *Kouleothrix* | *aurantiaca* | UP000050509 | 7836 | 1 | 1 | 0 | 1 | 1 | 9 | 0 | 122 |
| 187101 | *Sneathia* | *amnii* | UP000033103 | 1182 | 0 | 0 | 0 | 0 | 1 | 2 | 3 | 31 |
| 187272 | *Alkalilimnicola* | *ehrlichii* | UP000001962 | 2862 | 1 | 1 | 1 | 1 | 1 | 0 | 0 | 209 |
| 189518 | *Leptospira* | *interrogans* | UP000001408 | 3676 | 1 | 1 | 1 | 1 | 0 | 1 | 0 | 85 |
| 190485 | *Xanthomonas* | *campestris* | UP000001010 | 4127 | 1 | 1 | 1 | 1 | 0 | 2 | 1 | 190 |
| 190650 | *Caulobacter* | *crescentus* | UP000001816 | 3720 | 1 | 1 | 1 | 2 | 0 | 3 | 1 | 146 |
| 194439 | *Chlorobium* | *tepidum* | UP000001007 | 2250 | 2 | 1 | 1 | 2 | 1 | 2 | 0 | 62 |
| 195105 | *Haematobacter* | *massiliensis* | UP000028826 | 3806 | 1 | 1 | 1 | 2 | 0 | 1 | 0 | 134 |
| 197221 | *Thermosynechococcus* | *elongatus* | UP000000440 | 2451 | 1 | 1 | 1 | 1 | 2 | 1 | 1 | 84 |
| 198628 | *Dickeya* | *dadantii* | UP000006859 | 4532 | 1 | 0 | 0 | 2 | 1 | 8 | 1 | 335 |
| 203120 | *Leuconostoc* | *mesenteroides* | UP000000362 | 2002 | 1 | 0 | 0 | 2 | 1 | 2 | 0 | 70 |
| 203122 | *Saccharophagus* | *degradans* | UP000001947 | 3999 | 1 | 0 | 0 | 1 | 1 | 2 | 1 | 221 |
| 203123 | *Oenococcus* | *oeni* | UP000000774 | 1682 | 1 | 0 | 0 | 1 | 1 | 2 | 0 | 61 |
| 203124 | *Trichodesmium* | *erythraeum* | UP000008878 | 4342 | 1 | 0 | 3 | 1 | 1 | 1 | 0 | 132 |
| 203267 | *Tropheryma* | *whipplei* | UP000002200 | 805 | 0 | 0 | 0 | 0 | 1 | 1 | 0 | 30 |
| 203275 | *Tannerella* | *forsythia* | UP000005436 | 2978 | 1 | 0 | 1 | 0 | 1 | 2 | 0 | 73 |
| 203907 | *Blochmannia* | *floridanus* | UP000002192 | 583 | 0 | 0 | 0 | 0 | 1 | 0 | 1 | 32 |
| 204536 | *Sulfurihydrogenibium* | *azorense* | UP000001369 | 1708 | 0 | 0 | 1 | 1 | 1 | 1 | 1 | 95 |
| 204669 | *Koribacter* | *versatilis* | UP000002432 | 4771 | 1 | 2 | 0 | 1 | 1 | 6 | 1 | 179 |
| 204773 | *Herminiimonas* | *arsenicoxydans* | UP000006697 | 3272 | 1 | 1 | 1 | 1 | 1 | 0 | 0 | 200 |
| 206672 | *Bifidobacterium* | *longum* | UP000000439 | 1725 | 1 | 1 | 0 | 2 | 1 | 9 | 0 | 52 |
| 207949 | *Bermanella* | *marisrubri* | UP000004263 | 3303 | 1 | 0 | 2 | 1 | 1 | 1 | 1 | 195 |
| 207954 | *Neptuniibacter* | *caesariensis* | UP000002171 | 3687 | 1 | 2 | 1 | 1 | 1 | 1 | 1 | 214 |
| 212042 | *Anaplasma* | *phagocytophilum* | UP000001943 | 1330 | 0 | 0 | 0 | 0 | 0 | 0 | 0 | 23 |
| 215803 | *Enhygromyxa* | *salina* | UP000031599 | 8157 | 0 | 0 | 1 | 1 | 1 | 0 | 0 | 211 |
| 216432 | *Croceibacter* | *atlanticus* | UP000002297 | 2702 | 2 | 1 | 1 | 1 | 1 | 0 | 0 | 77 |
| 218491 | *Pectobacterium* | *atrosepticum* | UP000007966 | 4459 | 1 | 0 | 0 | 2 | 1 | 4 | 1 | 346 |
| 221109 | *Oceanobacillus* | *iheyensis* | UP000000822 | 3490 | 0 | 0 | 0 | 0 | 2 | 6 | 3 | 148 |
| 221126 | *Algibacter* | *lectus* | UP000029644 | 4609 | 1 | 1 | 1 | 0 | 1 | 0 | 0 | 69 |
| 221988 | *Mannheimia* | *succiniciproducens* | UP000000607 | 2367 | 0 | 0 | 1 | 1 | 1 | 2 | 0 | 76 |
| 222891 | *Neorickettsia* | *sennetsu* | UP000001942 | 932 | 0 | 0 | 1 | 0 | 0 | 0 | 0 | 22 |
| 224324 | *Aquifex* | *aeolicus* | UP000000798 | 1553 | 0 | 0 | 1 | 1 | 1 | 1 | 1 | 71 |
| 224911 | *Bradyrhizobium* | *diazoefficiens* | UP000002526 | 8253 | 1 | 3 | 1 | 2 | 0 | 1 | 0 | 290 |
| 226185 | *Enterococcus* | *faecalis* | UP000001415 | 3240 | 0 | 0 | 0 | 0 | 1 | 4 | 0 | 135 |
| 226186 | *Bacteroides* | *thetaiotaomicron* | UP000001414 | 4782 | 2 | 1 | 1 | 1 | 1 | 5 | 0 | 119 |
| 227377 | *Coxiella* | *burnetii* | UP000002671 | 1815 | 0 | 0 | 1 | 0 | 1 | 0 | 1 | 105 |
| 228400 | *Histophilus* | *somni* | UP000008543 | 1971 | 0 | 0 | 1 | 1 | 1 | 4 | 1 | 93 |
| 228405 | *Hyphomonas* | *neptunium* | UP000001959 | 3499 | 1 | 1 | 1 | 2 | 0 | 0 | 1 | 116 |
| 228410 | *Nitrosomonas* | *europaea* | UP000001416 | 2375 | 1 | 0 | 1 | 1 | 1 | 0 | 0 | 153 |
| 229920 | *Leptolinea* | *tardivitalis* | UP000050430 | 2936 | 0 | 2 | 1 | 0 | 0 | 5 | 1 | 106 |
| 229921 | *Levilinea* | *saccharolytica* | UP000050501 | 3080 | 1 | 2 | 1 | 0 | 0 | 8 | 1 | 94 |
| 234267 | *Solibacter* | *usitatus* | UP000000671 | 7761 | 1 | 2 | 0 | 1 | 1 | 6 | 2 | 263 |
| 234621 | *Rhodococcus* | *erythropolis* | UP000002204 | 6424 | 1 | 4 | 0 | 2 | 1 | 6 | 0 | 174 |
| 235909 | *Geobacillus* | *kaustophilus* | UP000001172 | 3516 | 0 | 0 | 0 | 0 | 2 | 4 | 3 | 147 |
| 240015 | *Acidobacterium* | *capsulatum* | UP000002207 | 3363 | 1 | 0 | 0 | 1 | 1 | 2 | 1 | 125 |
| 242619 | *Porphyromonas* | *gingivalis* | UP000000588 | 1863 | 1 | 0 | 1 | 0 | 1 | 0 | 0 | 56 |
| 243090 | *Rhodopirellula* | *baltica* | UP000001025 | 7271 | 1 | 2 | 1 | 1 | 1 | 2 | 0 | 117 |
| 243159 | *Acidithiobacillus* | *ferrooxidans* | UP000001362 | 3120 | 1 | 1 | 1 | 1 | 1 | 1 | 0 | 136 |
| 243164 | *Dehalococcoides* | *mccartyi* | UP000008289 | 1502 | 0 | 0 | 1 | 0 | 1 | 1 | 0 | 33 |
| 243231 | *Geobacter* | *sulfurreducens* | UP000000577 | 3402 | 1 | 1 | 1 | 2 | 1 | 1 | 1 | 202 |
| 243233 | *Methylococcus* | *capsulatus* | UP000006821 | 2925 | 1 | 1 | 1 | 1 | 1 | 1 | 1 | 156 |
| 243265 | *Photorhabdus* | *luminescens* | UP000002514 | 4556 | 1 | 0 | 1 | 2 | 1 | 2 | 1 | 367 |
| 243274 | *Thermotoga* | *maritima* | UP000008183 | 1852 | 0 | 0 | 1 | 0 | 1 | 6 | 0 | 60 |
| 243365 | *Chromobacterium* | *violaceum* | UP000001424 | 4397 | 1 | 2 | 1 | 1 | 1 | 1 | 0 | 341 |
| 244592 | *Labrenzia* | *alexandrii* | UP000004703 | 5363 | 1 | 3 | 1 | 2 | 1 | 4 | 0 | 191 |
| 246194 | *Carboxydothermus* | *hydrogenoformans* | UP000002706 | 2615 | 0 | 0 | 1 | 1 | 1 | 1 | 2 | 85 |
| 246195 | *Dichelobacter* | *nodosus* | UP000000248 | 1273 | 1 | 0 | 1 | 1 | 1 | 1 | 0 | 65 |
| 246197 | *Myxococcus* | *xanthus* | UP000002402 | 7314 | 1 | 1 | 1 | 2 | 1 | 3 | 0 | 299 |
| 246200 | *Ruegeria* | *pomeroyi* | UP000001023 | 4269 | 0 | 3 | 1 | 2 | 0 | 1 | 1 | 115 |
| 251221 | *Gloeobacter* | *violaceus* | UP000000557 | 4406 | 1 | 1 | 1 | 1 | 2 | 1 | 3 | 141 |
| 251229 | *Chroococcidiopsis* | *thermalis* | UP000010384 | 5740 | 1 | 2 | 2 | 1 | 1 | 3 | 0 | 268 |
| 257314 | *Lactobacillus* | *johnsonii* | UP000000581 | 1809 | 0 | 0 | 0 | 0 | 1 | 4 | 2 | 70 |
| 259536 | *Psychrobacter* | *arcticus* | UP000000546 | 2099 | 1 | 2 | 0 | 1 | 1 | 0 | 1 | 84 |
| 261317 | *Buchnera* | *aphidicola* | UP000006811 | 359 | 0 | 0 | 0 | 0 | 0 | 0 | 0 | 12 |
| 262316 | *Mycobacterium* | *paratuberculosis* | UP000000580 | 4316 | 1 | 1 | 0 | 2 | 1 | 3 | 0 | 162 |
| 262543 | *Exiguobacterium* | *sibiricum* | UP000001681 | 3005 | 1 | 1 | 0 | 1 | 1 | 2 | 1 | 132 |
| 1112230 | *Chlamydia* | *psittaci* | UP000014827 | 2871 | 0 | 0 | 0 | 0 | 0 | 0 | 0 | 38 |
| 263358 | *Verrucosispora* | *maris* | UP000008308 | 5998 | 1 | 0 | 1 | 2 | 1 | 14 | 0 | 181 |
| 264201 | *Protochlamydia* | *amoebophila* | UP000000529 | 2023 | 0 | 0 | 1 | 1 | 1 | 1 | 0 | 71 |
| 264203 | *Zymomonas* | *mobilis* | UP000001173 | 1779 | 1 | 1 | 1 | 2 | 1 | 1 | 0 | 104 |
| 264251 | *Cellulosimicrobium* | *funkei* | UP000035265 | 3980 | 1 | 1 | 1 | 2 | 1 | 10 | 0 | 99 |
| 264462 | *Bdellovibrio* | *bacteriovorus* | UP000008080 | 3583 | 1 | 0 | 0 | 1 | 1 | 1 | 0 | 120 |
| 264731 | *Prevotella* | *ruminicola* | UP000000927 | 2761 | 0 | 0 | 1 | 0 | 1 | 6 | 0 | 71 |
| 264732 | *Moorella* | *thermoacetica* | UP000007053 | 2451 | 0 | 0 | 1 | 1 | 1 | 2 | 1 | 87 |
| 265072 | *Methylobacillus* | *flagellatus* | UP000002440 | 2609 | 1 | 1 | 1 | 1 | 1 | 1 | 0 | 198 |
| 265311 | *Mesoplasma* | *florum* | UP000006647 | 683 | 0 | 0 | 0 | 0 | 0 | 2 | 0 | 15 |
| 266117 | *Rubrobacter* | *xylanophilus* | UP000006637 | 3127 | 1 | 0 | 1 | 0 | 1 | 3 | 1 | 93 |
| 266264 | *Cupriavidus* | *metallidurans* | UP000002429 | 6364 | 1 | 5 | 1 | 1 | 1 | 0 | 0 | 272 |
| 266835 | *Rhizobium* | *loti* | UP000000552 | 7255 | 1 | 2 | 1 | 2 | 0 | 6 | 0 | 296 |
| 266940 | *Kineococcus* | *radiotolerans* | UP000001116 | 4674 | 1 | 2 | 0 | 2 | 1 | 13 | 0 | 147 |
| 267608 | *Ralstonia* | *solanacearum* | UP000001436 | 5002 | 1 | 1 | 1 | 1 | 1 | 0 | 0 | 296 |
| 269798 | *Cytophaga* | *hutchinsonii* | UP000001822 | 3771 | 1 | 1 | 1 | 2 | 1 | 2 | 0 | 79 |
| 269800 | *Thermobifida* | *fusca* | UP000000434 | 3085 | 1 | 0 | 0 | 1 | 1 | 6 | 0 | 95 |
| 270498 | *Catabacter* | *hongkongensis* | UP000034076 | 3148 | 1 | 1 | 1 | 0 | 1 | 11 | 0 | 120 |
| 272123 | *Anabaena* | *cylindrica* | UP000010474 | 5797 | 1 | 1 | 2 | 1 | 1 | 2 | 0 | 165 |
| 272560 | *Burkholderia* | *pseudomallei* | UP000000605 | 5717 | 1 | 2 | 1 | 1 | 1 | 1 | 0 | 510 |
| 272563 | *Peptoclostridium* | *difficile* | UP000001978 | 3762 | 0 | 0 | 0 | 1 | 1 | 4 | 1 | 137 |
| 272568 | *Gluconacetobacter* | *diazotrophicus* | UP000001176 | 3783 | 1 | 1 | 1 | 2 | 1 | 1 | 1 | 134 |
| 272624 | *Legionella* | *pneumophila* | UP000000609 | 2930 | 1 | 0 | 1 | 0 | 1 | 0 | 1 | 525 |
| 272635 | *Mycoplasma* | *pulmonis* | UP000000528 | 778 | 0 | 0 | 0 | 0 | 0 | 1 | 0 | 12 |
| 272843 | *Pasteurella* | *multocida* | UP000000809 | 2015 | 0 | 0 | 1 | 1 | 1 | 4 | 0 | 134 |
| 272942 | *Rhodobacter* | *capsulatus* | UP000002361 | 3632 | 1 | 2 | 1 | 2 | 0 | 3 | 1 | 175 |
| 573234 | *Hodgkinia* | *cicadicola* | UP000002741 | 169 | 0 | 0 | 0 | 0 | 0 | 0 | 0 | 3 |
| 273068 | *Caldanaerobacter* | *subterraneus* | UP000000555 | 2545 | 0 | 0 | 1 | 1 | 1 | 5 | 1 | 104 |
| 1399147 | *Holospora* | *obtusa* | UP000019112 | 1116 | 0 | 0 | 0 | 0 | 0 | 0 | 1 | 30 |
| 273121 | *Wolinella* | *succinogenes* | UP000000422 | 2028 | 1 | 1 | 1 | 2 | 1 | 0 | 1 | 155 |
| 278197 | *Pediococcus* | *pentosaceus* | UP000000773 | 1755 | 0 | 0 | 0 | 1 | 1 | 6 | 0 | 58 |
| 281090 | *Leifsonia* | *xyli* | UP000001306 | 2001 | 0 | 0 | 0 | 0 | 1 | 8 | 0 | 61 |
| 281689 | *Desulfuromonas* | *acetoxidans* | UP000005695 | 3204 | 1 | 1 | 1 | 2 | 1 | 0 | 1 | 215 |
| 283942 | *Idiomarina* | *loihiensis* | UP000001171 | 2608 | 1 | 0 | 1 | 2 | 1 | 0 | 0 | 158 |
| 287752 | *Aurantimonas* | *manganoxydans* | UP000000321 | 3625 | 1 | 1 | 1 | 2 | 1 | 1 | 0 | 154 |
| 288705 | *Renibacterium* | *salmoninarum* | UP000002007 | 3421 | 1 | 2 | 0 | 2 | 1 | 10 | 0 | 93 |
| 289376 | *Thermodesulfovibrio* | *yellowstonii* | UP000000718 | 1982 | 0 | 0 | 1 | 1 | 1 | 1 | 0 | 114 |
| 290338 | *Citrobacter* | *koseri* | UP000008148 | 5019 | 1 | 0 | 1 | 2 | 1 | 7 | 1 | 377 |
| 290339 | *Cronobacter* | *sakazakii* | UP000000260 | 4421 | 1 | 0 | 1 | 2 | 1 | 6 | 1 | 291 |
| 290340 | *Arthrobacter* | *aurescens* | UP000000637 | 4565 | 1 | 2 | 0 | 2 | 1 | 9 | 0 | 116 |
| 290397 | *Anaeromyxobacter* | *dehalogenans* | UP000001935 | 4345 | 0 | 0 | 1 | 1 | 1 | 1 | 2 | 199 |
| 290398 | *Chromohalobacter* | *salexigens* | UP000000239 | 3298 | 1 | 0 | 1 | 1 | 1 | 1 | 1 | 209 |
| 290512 | *Prosthecochloris* | *aestuarii* | UP000002725 | 2319 | 1 | 1 | 1 | 2 | 1 | 2 | 0 | 72 |
| 290633 | *Gluconobacter* | *oxydans* | UP000006375 | 2626 | 1 | 2 | 0 | 2 | 1 | 3 | 1 | 123 |
| 292415 | *Thiobacillus* | *denitrificans* | UP000008291 | 2826 | 1 | 1 | 1 | 1 | 1 | 1 | 0 | 183 |
| 292459 | *Symbiobacterium* | *thermophilum* | UP000000417 | 3313 | 0 | 0 | 1 | 0 | 1 | 4 | 2 | 116 |
| 292563 | *Cyanobacterium* | *stanieri* | UP000010483 | 2831 | 1 | 1 | 2 | 1 | 1 | 1 | 0 | 94 |
| 292564 | *Cyanobium* | *gracile* | UP000010388 | 3261 | 1 | 1 | 1 | 1 | 1 | 1 | 0 | 94 |
| 300267 | *Shigella* | *dysenteriae* | UP000002716 | 3897 | 1 | 0 | 1 | 2 | 1 | 6 | 1 | 276 |
| 300852 | *Thermus* | *thermophilus* | UP000000532 | 2227 | 0 | 0 | 2 | 0 | 0 | 2 | 0 | 52 |
| 302409 | *Ehrlichia* | *ruminantium* | UP000000533 | 948 | 0 | 0 | 1 | 0 | 0 | 0 | 0 | 23 |
| 305900 | *Endozoicomonas* | *elysicola* | UP000027997 | 4147 | 1 | 0 | 1 | 1 | 1 | 4 | 1 | 164 |
| 309798 | *Coprothermobacter* | *proteolyticus* | UP000001732 | 1481 | 0 | 0 | 1 | 0 | 1 | 1 | 0 | 50 |
| 309801 | *Thermomicrobium* | *roseum* | UP000000447 | 2858 | 0 | 0 | 0 | 0 | 1 | 2 | 2 | 81 |
| 309807 | *Salinibacter* | *ruber* | UP000008674 | 2812 | 0 | 0 | 1 | 2 | 1 | 1 | 0 | 87 |
| 312153 | *Polynucleobacter* | *necessarius* | UP000000231 | 2074 | 0 | 2 | 1 | 1 | 1 | 0 | 0 | 78 |
| 312309 | *Vibrio* | *fischeri* | UP000000537 | 3813 | 0 | 0 | 1 | 1 | 1 | 5 | 1 | 262 |
| 313594 | *Polaribacter* | *irgensii* | UP000003053 | 2556 | 0 | 2 | 1 | 1 | 1 | 1 | 0 | 56 |
| 313595 | *Psychroflexus* | *torquis* | UP000008514 | 3457 | 1 | 1 | 1 | 1 | 1 | 0 | 0 | 70 |
| 313596 | *Robiginitalea* | *biformata* | UP000009049 | 3211 | 1 | 1 | 1 | 1 | 1 | 0 | 1 | 70 |
| 313606 | *Microscilla* | *marina* | UP000004095 | 8248 | 1 | 1 | 1 | 2 | 1 | 1 | 0 | 123 |
| 313624 | *Nodularia* | *spumigena* | UP000019325 | 5211 | 1 | 1 | 2 | 1 | 1 | 2 | 0 | 157 |
| 313628 | *Lentisphaera* | *araneosa* | UP000004947 | 4982 | 1 | 1 | 0 | 1 | 0 | 3 | 0 | 94 |
| 314230 | *Blastopirellula* | *marina* | UP000004358 | 6010 | 1 | 2 | 1 | 1 | 1 | 2 | 0 | 149 |
| 314231 | *Fulvimarina* | *pelagi* | UP000004310 | 3739 | 1 | 0 | 1 | 2 | 1 | 2 | 0 | 118 |
| 314232 | *Loktanella* | *vestfoldensis* | UP000004507 | 3061 | 1 | 1 | 1 | 2 | 0 | 3 | 1 | 100 |
| 314256 | *Oceanicola* | *granulosus* | UP000003635 | 3791 | 1 | 1 | 1 | 3 | 0 | 4 | 1 | 141 |
| 314260 | *Parvularcula* | *bermudensis* | UP000001302 | 2687 | 1 | 0 | 1 | 2 | 0 | 0 | 1 | 99 |
| 314265 | *Pelagibaca* | *bermudensis* | UP000006230 | 5420 | 1 | 2 | 1 | 2 | 0 | 0 | 1 | 184 |
| 314271 | *Maritimibacter* | *alkaliphilus* | UP000002931 | 4692 | 1 | 2 | 1 | 2 | 0 | 1 | 1 | 112 |
| 314275 | *Alteromonas* | *mediterranea* | UP000001870 | 3938 | 1 | 1 | 0 | 2 | 1 | 0 | 0 | 195 |
| 314278 | *Nitrococcus* | *mobilis* | UP000003374 | 3484 | 1 | 0 | 1 | 1 | 1 | 0 | 0 | 200 |
| 314283 | *Reinekea* | *blandensis* | UP000005953 | 4236 | 1 | 0 | 0 | 2 | 1 | 3 | 1 | 229 |
| 314285 | *Congregibacter* | *litoralis* | UP000019205 | 3868 | 1 | 2 | 0 | 1 | 1 | 1 | 0 | 171 |
| 314345 | *Mariprofundus* | *ferrooxydans* | UP000005297 | 2856 | 1 | 2 | 0 | 2 | 1 | 2 | 0 | 129 |
| 314724 | *Borrelia* | *turicatae* | UP000001205 | 818 | 0 | 0 | 0 | 0 | 1 | 1 | 0 | 33 |
| 316274 | *Herpetosiphon* | *aurantiacus* | UP000000787 | 5254 | 1 | 1 | 1 | 1 | 1 | 7 | 0 | 204 |
| 317025 | *Thiomicrospira* | *crunogena* | UP000002713 | 2187 | 1 | 1 | 1 | 1 | 1 | 1 | 1 | 149 |
| 317619 | *Prochlorothrix* | *hollandica* | UP000034681 | 3617 | 0 | 1 | 2 | 1 | 1 | 0 | 1 | 98 |
| 317655 | *Sphingopyxis* | *alaskensis* | UP000006578 | 3184 | 1 | 1 | 1 | 2 | 0 | 0 | 0 | 114 |
| 318167 | *Shewanella* | *frigidimarina* | UP000000684 | 3990 | 0 | 0 | 1 | 1 | 1 | 1 | 1 | 223 |
| 318586 | *Paracoccus* | *denitrificans* | UP000000361 | 5019 | 1 | 3 | 1 | 2 | 0 | 1 | 1 | 222 |
| 320771 | *Pedosphaera* | *parvula* | UP000003688 | 6492 | 1 | 1 | 0 | 1 | 1 | 3 | 0 | 194 |
| 322710 | *Azotobacter* | *vinelandii* | UP000002424 | 4990 | 1 | 3 | 1 | 1 | 1 | 1 | 1 | 308 |
| 323097 | *Nitrobacter* | *hamburgensis* | UP000001953 | 4243 | 1 | 2 | 1 | 2 | 0 | 0 | 0 | 167 |
| 323848 | *Nitrosospira* | *multiformis* | UP000002718 | 2732 | 1 | 1 | 1 | 1 | 1 | 0 | 0 | 158 |
| 324602 | *Chloroflexus* | *aurantiacus* | UP000002008 | 3850 | 1 | 1 | 1 | 1 | 1 | 3 | 1 | 133 |
| 324925 | *Pelodictyon* | *phaeoclathratiforme* | UP000002724 | 2685 | 1 | 1 | 1 | 2 | 1 | 2 | 1 | 94 |
| 326298 | *Sulfurimonas* | *denitrificans* | UP000002714 | 2084 | 1 | 1 | 1 | 1 | 1 | 0 | 1 | 141 |
| 326424 | *Frankia* | *alni* | UP000000657 | 6710 | 1 | 2 | 1 | 2 | 1 | 5 | 1 | 178 |
| 329726 | *Acaryochloris* | *marina* | UP000000268 | 8172 | 1 | 1 | 1 | 1 | 1 | 1 | 1 | 199 |
| 330214 | *Nitrospira* | *defluvii* | UP000001660 | 4263 | 1 | 2 | 1 | 2 | 1 | 0 | 1 | 202 |
| 331113 | *Simkania* | *negevensis* | UP000000496 | 2516 | 0 | 0 | 1 | 0 | 1 | 1 | 0 | 81 |
| 332411 | *Aquitalea* | *magnusonii* | UP000033764 | 4103 | 1 | 2 | 1 | 1 | 1 | 0 | 0 | 243 |
| 334413 | *Finegoldia* | *magna* | UP000001319 | 1813 | 1 | 1 | 0 | 1 | 1 | 0 | 1 | 65 |
| 335541 | *Syntrophomonas* | *wolfei* | UP000001968 | 2473 | 0 | 0 | 1 | 1 | 1 | 1 | 0 | 99 |
| 335543 | *Syntrophobacter* | *fumaroxidans* | UP000001784 | 4012 | 0 | 0 | 1 | 1 | 1 | 3 | 1 | 168 |
| 335992 | *Pelagibacter* | *ubique* | UP000002528 | 1354 | 0 | 0 | 0 | 0 | 0 | 1 | 1 | 44 |
| 338565 | *Erwinia* | *tasmaniensis* | UP000001726 | 3618 | 1 | 0 | 1 | 2 | 1 | 3 | 1 | 275 |
| 338963 | *Pelobacter* | *carbinolicus* | UP000002534 | 3292 | 0 | 0 | 1 | 1 | 1 | 1 | 1 | 208 |
| 338969 | *Rhodoferax* | *ferrireducens* | UP000008332 | 4401 | 1 | 2 | 1 | 1 | 1 | 2 | 0 | 262 |
| 339671 | *Actinobacillus* | *succinogenes* | UP000001114 | 2077 | 0 | 0 | 1 | 1 | 1 | 2 | 0 | 106 |
| 340071 | *Criblamydia* | *sequanensis* | UP000031552 | 2418 | 0 | 0 | 1 | 0 | 1 | 1 | 0 | 80 |
| 340099 | *Thermoanaerobacter* | *pseudethanolicus* | UP000002156 | 2193 | 0 | 0 | 1 | 1 | 1 | 6 | 2 | 82 |
| 340100 | *Bordetella* | *petrii* | UP000001225 | 4912 | 1 | 1 | 1 | 1 | 1 | 0 | 0 | 268 |
| 342108 | *Magnetospirillum* | *magneticum* | UP000007058 | 4514 | 1 | 2 | 1 | 2 | 0 | 1 | 2 | 168 |
| 342610 | *Pseudoalteromonas* | *atlantica* | UP000001981 | 4271 | 1 | 1 | 1 | 2 | 1 | 0 | 0 | 198 |
| 343509 | *Sodalis* | *glossinidius* | UP000001932 | 2490 | 1 | 0 | 1 | 2 | 1 | 3 | 1 | 171 |
| 344747 | *Gimesia* | *maris* | UP000003087 | 6463 | 1 | 1 | 1 | 1 | 1 | 4 | 0 | 163 |
| 345309 | *Luteibacter* | *yeojuensis* | UP000033651 | 3844 | 1 | 1 | 1 | 1 | 0 | 0 | 0 | 216 |
| 349124 | *Halorhodospira* | *halophila* | UP000000647 | 2406 | 1 | 1 | 1 | 1 | 1 | 0 | 0 | 149 |
| 349161 | *Desulfotomaculum* | *reducens* | UP000001556 | 3220 | 0 | 0 | 1 | 2 | 1 | 0 | 1 | 124 |
| 349163 | *Acidiphilium* | *cryptum* | UP000000245 | 3521 | 1 | 1 | 0 | 2 | 0 | 2 | 1 | 181 |
| 349521 | *Hahella* | *chejuensis* | UP000000238 | 6751 | 1 | 1 | 1 | 1 | 1 | 0 | 1 | 404 |
| 349741 | *Akkermansia* | *muciniphila* | UP000001031 | 2137 | 1 | 2 | 0 | 0 | 1 | 2 | 1 | 68 |
| 350688 | *Alkaliphilus* | *oremlandii* | UP000000269 | 2828 | 0 | 0 | 0 | 1 | 1 | 0 | 2 | 124 |
| 351348 | *Marinobacter* | *hydrocarbonoclasticus* | UP000000998 | 4170 | 1 | 1 | 1 | 1 | 1 | 1 | 1 | 244 |
| 351607 | *Acidothermus* | *cellulolyticus* | UP000008221 | 2157 | 0 | 0 | 0 | 1 | 1 | 5 | 1 | 86 |
| 351627 | *Caldicellulosiruptor* | *saccharolyticus* | UP000000256 | 2629 | 0 | 0 | 0 | 0 | 1 | 2 | 1 | 91 |
| 352165 | *Pyramidobacter* | *piscolens* | UP000006462 | 2755 | 0 | 0 | 0 | 1 | 1 | 0 | 2 | 72 |
| 696127 | *Midichloria* | *mitochondrii* | UP000006639 | 1181 | 0 | 0 | 0 | 0 | 0 | 0 | 0 | 43 |
| 357804 | *Psychromonas* | *ingrahamii* | UP000000639 | 3530 | 1 | 0 | 1 | 2 | 1 | 3 | 0 | 189 |
| 357809 | *Clostridium* | *phytofermentans* | UP000000370 | 3891 | 0 | 0 | 0 | 0 | 1 | 4 | 0 | 132 |
| 358681 | *Brevibacillus* | *brevis* | UP000001877 | 5887 | 0 | 0 | 1 | 0 | 1 | 5 | 0 | 239 |
| 359391 | *Brucella* | *abortus* | UP000002719 | 3022 | 1 | 1 | 1 | 2 | 1 | 2 | 0 | 152 |
| 360095 | *Bartonella* | *bacilliformis* | UP000000643 | 1255 | 0 | 0 | 0 | 0 | 1 | 0 | 0 | 67 |
| 360106 | *Campylobacter* | *fetus* | UP000000760 | 1715 | 1 | 1 | 1 | 1 | 1 | 0 | 1 | 143 |
| 360411 | *Bellilinea* | *caldifistulae* | UP000050514 | 2990 | 1 | 1 | 1 | 1 | 0 | 5 | 1 | 108 |
| 363253 | *Lawsonia* | *intracellularis* | UP000002430 | 1342 | 0 | 0 | 1 | 0 | 1 | 0 | 1 | 84 |
| 365044 | *Polaromonas* | *naphthalenivorans* | UP000000644 | 4879 | 1 | 4 | 1 | 1 | 1 | 1 | 0 | 171 |
| 365046 | *Ramlibacter* | *tataouinensis* | UP000008385 | 3880 | 1 | 0 | 1 | 1 | 1 | 2 | 0 | 132 |
| 369723 | *Salinispora* | *tropica* | UP000000235 | 4522 | 1 | 0 | 0 | 2 | 1 | 7 | 0 | 165 |
| 370438 | *Pelotomaculum* | *thermopropionicum* | UP000006556 | 2884 | 0 | 0 | 1 | 1 | 1 | 1 | 0 | 74 |
| 370622 | *Aureimonas* | *altamirensis* | UP000030826 | 3519 | 1 | 2 | 1 | 1 | 1 | 1 | 0 | 154 |
| 373903 | *Halothermothrix* | *orenii* | UP000000719 | 2324 | 0 | 0 | 1 | 1 | 1 | 5 | 2 | 98 |
| 374463 | *Baumannia* | *cicadellinicola* | UP000002427 | 595 | 0 | 0 | 0 | 0 | 1 | 0 | 1 | 17 |
| 375451 | *Roseobacter* | *denitrificans* | UP000007029 | 4118 | 1 | 2 | 1 | 2 | 0 | 1 | 1 | 143 |
| 377629 | *Teredinibacter* | *turnerae* | UP000009080 | 4249 | 1 | 2 | 0 | 1 | 1 | 2 | 1 | 259 |
| 378753 | *Kocuria* | *rhizophila* | UP000008838 | 2352 | 0 | 1 | 1 | 2 | 1 | 2 | 0 | 61 |
| 378806 | *Stigmatella* | *aurantiaca* | UP000001351 | 8307 | 1 | 1 | 1 | 3 | 1 | 6 | 0 | 314 |
| 379066 | *Gemmatimonas* | *aurantiaca* | UP000002209 | 3932 | 1 | 1 | 1 | 1 | 1 | 2 | 0 | 167 |
| 380703 | *Aeromonas* | *hydrophila* | UP000000756 | 4121 | 1 | 1 | 1 | 2 | 1 | 5 | 1 | 296 |
| 381764 | *Fervidobacterium* | *nodosum* | UP000002415 | 1725 | 0 | 0 | 1 | 0 | 1 | 2 | 0 | 64 |
| 388399 | *Sagittula* | *stellata* | UP000005713 | 5025 | 1 | 1 | 1 | 2 | 0 | 1 | 1 | 151 |
| 388413 | *Algoriphagus* | *machipongonensis* | UP000003919 | 3932 | 1 | 1 | 1 | 2 | 1 | 3 | 0 | 90 |
| 388467 | *Planktothrix* | *agardhii* | UP000027395 | 4186 | 1 | 2 | 1 | 1 | 2 | 1 | 0 | 118 |
| 391009 | *Thermosipho* | *melanesiensis* | UP000001110 | 1877 | 0 | 0 | 1 | 0 | 1 | 1 | 0 | 62 |
| 391165 | *Granulibacter* | *bethesdensis* | UP000001963 | 2430 | 1 | 2 | 0 | 2 | 1 | 1 | 1 | 104 |
| 391295 | *Streptococcus* | *suis* | UP000000243 | 2178 | 0 | 0 | 0 | 0 | 1 | 3 | 0 | 85 |
| 391587 | *Kordia* | *algicida* | UP000002945 | 4477 | 1 | 1 | 1 | 1 | 1 | 0 | 1 | 92 |
| 391619 | *Phaeobacter* | *inhibens* | UP000002914 | 3853 | 0 | 2 | 1 | 2 | 0 | 2 | 1 | 139 |
| 391624 | *Oceanibulbus* | *indolifex* | UP000003257 | 4147 | 1 | 1 | 1 | 2 | 1 | 1 | 1 | 118 |
| 391625 | *Plesiocystis* | *pacifica* | UP000005801 | 8437 | 0 | 0 | 0 | 1 | 1 | 4 | 0 | 184 |
| 391626 | *Octadecabacter* | *antarcticus* | UP000005307 | 4327 | 1 | 1 | 1 | 2 | 0 | 4 | 1 | 120 |
| 391735 | *Verminephrobacter* | *eiseniae* | UP000000374 | 4911 | 1 | 2 | 1 | 1 | 1 | 2 | 0 | 222 |
| 392499 | *Sphingomonas* | *wittichii* | UP000001989 | 5313 | 1 | 2 | 1 | 2 | 0 | 1 | 0 | 164 |
| 394096 | *Hyalangium* | *minutum* | UP000028725 | 8967 | 1 | 1 | 1 | 2 | 1 | 2 | 0 | 323 |
| 394221 | *Maricaulis* | *maris* | UP000001964 | 3060 | 1 | 1 | 1 | 2 | 0 | 1 | 1 | 103 |
| 395493 | *Beggiatoa* | *alba* | UP000005744 | 3440 | 1 | 1 | 1 | 1 | 1 | 0 | 0 | 173 |
| 395494 | *Gallionella* | *capsiferriformans* | UP000001235 | 2853 | 1 | 1 | 1 | 1 | 1 | 0 | 0 | 206 |
| 395495 | *Leptothrix* | *cholodnii* | UP000001693 | 4343 | 1 | 3 | 1 | 2 | 1 | 1 | 0 | 259 |
| 395963 | *Beijerinckia* | *indica* | UP000001695 | 3774 | 1 | 2 | 1 | 2 | 1 | 2 | 0 | 153 |
| 395965 | *Methylocella* | *silvestris* | UP000002257 | 3816 | 1 | 2 | 1 | 2 | 1 | 1 | 0 | 161 |
| 396513 | *Staphylococcus* | *carnosus* | UP000000444 | 2460 | 1 | 1 | 0 | 1 | 1 | 2 | 0 | 85 |
| 396588 | *Thioalkalivibrio* | *sulfidiphilus* | UP000002383 | 3272 | 1 | 2 | 1 | 1 | 1 | 1 | 0 | 209 |
| 397945 | *Acidovorax* | *citrulli* | UP000002596 | 4602 | 1 | 1 | 1 | 1 | 1 | 1 | 0 | 283 |
| 398578 | *Delftia* | *acidovorans* | UP000000784 | 5968 | 1 | 2 | 1 | 2 | 1 | 0 | 0 | 320 |
| 398580 | *Dinoroseobacter* | *shibae* | UP000006833 | 4086 | 1 | 2 | 1 | 2 | 0 | 1 | 1 | 128 |
| 398720 | *Leeuwenhoekiella* | *blandensis* | UP000001601 | 3687 | 2 | 1 | 1 | 1 | 1 | 2 | 0 | 70 |
| 399739 | *Pseudomonas* | *mendocina* | UP000000229 | 4563 | 1 | 3 | 1 | 1 | 1 | 0 | 1 | 376 |
| 399741 | *Serratia* | *proteamaculans* | UP000007074 | 4930 | 1 | 1 | 1 | 2 | 1 | 7 | 1 | 335 |
| 400092 | *Pontibacter* | *korlensis* | UP000033109 | 4103 | 1 | 0 | 1 | 2 | 1 | 4 | 0 | 113 |
| 401526 | *Thermosinus* | *carboxydivorans* | UP000005139 | 2738 | 0 | 0 | 1 | 1 | 1 | 1 | 2 | 117 |
| 402881 | *Parvibaculum* | *lavamentivorans* | UP000006377 | 3580 | 1 | 1 | 1 | 2 | 1 | 1 | 1 | 125 |
| 405948 | *Saccharopolyspora* | *erythraea* | UP000006728 | 7154 | 1 | 1 | 0 | 3 | 1 | 10 | 0 | 196 |
| 406818 | *Xenorhabdus* | *bovienii* | UP000002045 | 4102 | 1 | 0 | 1 | 2 | 1 | 3 | 1 | 266 |
| 407035 | *Salinicoccus* | *halodurans* | UP000034029 | 2634 | 1 | 2 | 0 | 1 | 0 | 3 | 0 | 89 |
| 411154 | *Gramella* | *forsetii* | UP000000755 | 3554 | 1 | 1 | 1 | 2 | 1 | 1 | 0 | 78 |
| 411467 | *Pseudoflavonifractor* | *capillosus* | UP000003639 | 4804 | 1 | 0 | 0 | 1 | 1 | 3 | 0 | 124 |
| 411471 | *Subdoligranulum* | *variabile* | UP000003438 | 3378 | 1 | 1 | 0 | 0 | 1 | 3 | 0 | 102 |
| 411475 | *Flavonifractor* | *plautii* | UP000004459 | 4278 | 0 | 0 | 0 | 0 | 1 | 3 | 0 | 123 |
| 411483 | *Faecalibacterium* | *prausnitzii* | UP000004619 | 3472 | 1 | 0 | 0 | 0 | 1 | 4 | 0 | 76 |
| 411684 | *Hoeflea* | *phototrophica* | UP000004291 | 4185 | 1 | 2 | 1 | 2 | 0 | 1 | 0 | 174 |
| 413404 | *Ruthia* | *magnifica* | UP000002587 | 976 | 0 | 0 | 1 | 0 | 1 | 0 | 1 | 43 |
| 413882 | *Polyangium* | *brachysporum* | UP000035352 | 5541 | 1 | 1 | 1 | 1 | 1 | 2 | 0 | 281 |
| 414684 | *Rhodospirillum* | *centenum* | UP000001591 | 3984 | 1 | 1 | 1 | 2 | 0 | 1 | 1 | 146 |
| 414996 | *Actinopolyspora* | *erythraea* | UP000029737 | 3972 | 1 | 0 | 0 | 2 | 1 | 4 | 0 | 103 |
| 416591 | *Pseudothermotoga* | *lettingae* | UP000002016 | 2040 | 0 | 0 | 1 | 0 | 1 | 7 | 0 | 70 |
| 420662 | *Methylibium* | *petroleiphilum* | UP000000366 | 4359 | 1 | 2 | 1 | 1 | 1 | 1 | 0 | 208 |
| 420890 | *Lactococcus* | *garvieae* | UP000008520 | 1938 | 0 | 0 | 0 | 0 | 1 | 3 | 0 | 68 |
| 426355 | *Methylobacterium* | *radiotolerans* | UP000006589 | 6388 | 1 | 1 | 1 | 2 | 1 | 1 | 0 | 260 |
| 429009 | *Ammonifex* | *degensii* | UP000002620 | 2077 | 0 | 0 | 1 | 1 | 1 | 1 | 0 | 72 |
| 435591 | *Parabacteroides* | *distasonis* | UP000000566 | 3830 | 1 | 0 | 1 | 0 | 1 | 7 | 0 | 125 |
| 438753 | *Azorhizobium* | *caulinodans* | UP000000270 | 4708 | 1 | 1 | 1 | 2 | 1 | 0 | 0 | 206 |
| 439235 | *Desulfatibacillum* | *alkenivorans* | UP000000739 | 5198 | 0 | 0 | 1 | 0 | 1 | 0 | 1 | 202 |
| 439375 | *Ochrobactrum* | *anthropi* | UP000002301 | 4757 | 1 | 1 | 1 | 2 | 1 | 3 | 0 | 209 |
| 441768 | *Acholeplasma* | *laidlawii* | UP000008558 | 1380 | 0 | 0 | 1 | 1 | 0 | 1 | 0 | 52 |
| 443218 | *Amycolicicoccus* | *subflavus* | UP000009235 | 4703 | 1 | 1 | 0 | 2 | 1 | 5 | 0 | 128 |
| 443254 | *Marinitoga* | *piezophila* | UP000007161 | 2044 | 0 | 0 | 1 | 0 | 1 | 2 | 0 | 72 |
| 903503 | *Moranella* | *endobia* | UP000000504 | 405 | 0 | 0 | 0 | 0 | 0 | 0 | 1 | 18 |
| 445709 | *Pandoraea* | *thiooxydans* | UP000036700 | 3963 | 1 | 1 | 1 | 1 | 1 | 0 | 0 | 211 |
| 445932 | *Elusimicrobium* | *minutum* | UP000001029 | 1528 | 0 | 0 | 0 | 0 | 1 | 1 | 1 | 45 |
| 445971 | *Anaerofustis* | *stercorihominis* | UP000005178 | 2331 | 0 | 0 | 0 | 1 | 1 | 0 | 0 | 54 |
| 445972 | *Anaerotruncus* | *colihominis* | UP000003803 | 4421 | 1 | 1 | 0 | 0 | 1 | 9 | 1 | 98 |
| 446462 | *Actinosynnema* | *mirum* | UP000002213 | 6912 | 1 | 1 | 1 | 3 | 1 | 15 | 0 | 241 |
| 446465 | *Brachybacterium* | *faecium* | UP000001919 | 3062 | 0 | 2 | 0 | 1 | 1 | 14 | 0 | 91 |
| 446468 | *Nocardiopsis* | *dassonvillei* | UP000002219 | 5497 | 1 | 0 | 0 | 1 | 1 | 10 | 1 | 164 |
| 446469 | *Sanguibacter* | *keddieii* | UP000000322 | 3710 | 1 | 1 | 0 | 1 | 1 | 13 | 0 | 119 |
| 446470 | *Stackebrandtia* | *nassauensis* | UP000000844 | 6379 | 1 | 0 | 0 | 1 | 1 | 11 | 0 | 161 |
| 446471 | *Xylanimonas* | *cellulosilytica* | UP000002255 | 3441 | 1 | 1 | 1 | 2 | 1 | 9 | 0 | 93 |
| 448385 | *Sorangium* | *cellulosum* | UP000002139 | 9320 | 1 | 1 | 1 | 2 | 0 | 1 | 1 | 354 |
| 449447 | *Microcystis* | *aeruginosa* | UP000001510 | 5981 | 1 | 2 | 1 | 1 | 1 | 1 | 0 | 118 |
| 450851 | *Phenylobacterium* | *zucineum* | UP000001868 | 3838 | 1 | 1 | 1 | 2 | 1 | 1 | 1 | 146 |
| 452471 | *Amoebophilus* | *asiaticus* | UP000001227 | 1253 | 0 | 0 | 0 | 1 | 0 | 0 | 1 | 66 |
| 452637 | *Opitutus* | *terrae* | UP000007013 | 4588 | 1 | 1 | 1 | 1 | 1 | 2 | 1 | 162 |
| 452652 | *Kitasatospora* | *setae* | UP000007076 | 7443 | 1 | 1 | 0 | 2 | 1 | 20 | 1 | 210 |
| 454194 | *Pyrinomonas* | *methylaliphatogenes* | UP000031518 | 3179 | 0 | 0 | 1 | 0 | 1 | 8 | 3 | 167 |
| 457570 | *Natranaerobius* | *thermophilus* | UP000001683 | 2847 | 0 | 0 | 1 | 1 | 1 | 1 | 2 | 88 |
| 458233 | *Macrococcus* | *caseolyticus* | UP000001383 | 2059 | 1 | 1 | 0 | 1 | 1 | 1 | 0 | 83 |
| 459349 | *Cloacimonas* | *acidaminovorans* | UP000002019 | 1813 | 0 | 0 | 1 | 1 | 1 | 1 | 0 | 60 |
| 465515 | *Micrococcus* | *luteus* | UP000000738 | 2207 | 0 | 3 | 0 | 2 | 1 | 1 | 0 | 67 |
| 469371 | *Thermobispora* | *bispora* | UP000006640 | 3545 | 0 | 0 | 0 | 1 | 1 | 7 | 2 | 108 |
| 469378 | *Cryptobacterium* | *curtum* | UP000000954 | 1355 | 1 | 1 | 0 | 1 | 1 | 1 | 1 | 53 |
| 469381 | *Dethiosulfovibrio* | *peptidovorans* | UP000006427 | 2432 | 1 | 1 | 1 | 2 | 1 | 1 | 1 | 104 |
| 469383 | *Conexibacter* | *woesei* | UP000008229 | 5912 | 1 | 1 | 1 | 3 | 1 | 8 | 1 | 166 |
| 469605 | *Fusobacterium* | *gonidiaformans* | UP000002975 | 1890 | 0 | 0 | 0 | 1 | 1 | 4 | 0 | 57 |
| 471852 | *Thermomonospora* | *curvata* | UP000001918 | 4888 | 1 | 0 | 0 | 2 | 1 | 4 | 1 | 133 |
| 471853 | *Beutenbergia* | *cavernae* | UP000007962 | 4195 | 1 | 1 | 1 | 1 | 1 | 16 | 0 | 104 |
| 471854 | *Dyadobacter* | *fermentans* | UP000002011 | 5703 | 2 | 1 | 1 | 2 | 1 | 5 | 0 | 142 |
| 471855 | *Slackia* | *heliotrinireducens* | UP000002026 | 2750 | 1 | 2 | 0 | 1 | 1 | 1 | 1 | 84 |
| 471856 | *Jonesia* | *denitrificans* | UP000000628 | 2495 | 1 | 0 | 0 | 1 | 1 | 9 | 0 | 89 |
| 471857 | *Saccharomonospora* | *viridis* | UP000000841 | 3828 | 1 | 0 | 0 | 3 | 1 | 10 | 0 | 104 |
| 471875 | *Ruminococcus* | *lactaris* | UP000003254 | 2706 | 1 | 1 | 0 | 0 | 1 | 3 | 0 | 62 |
| 471881 | *Proteus* | *penneri* | UP000006464 | 4909 | 1 | 0 | 1 | 2 | 1 | 5 | 1 | 229 |
| 472759 | *Nitrosococcus* | *halophilus* | UP000001844 | 3770 | 1 | 1 | 1 | 1 | 1 | 0 | 0 | 187 |
| 477974 | *Desulforudis* | *audaxviator* | UP000008544 | 2136 | 0 | 0 | 1 | 1 | 1 | 1 | 1 | 79 |
| 478749 | *Marvinbryantia* | *formatexigens* | UP000005561 | 4894 | 2 | 0 | 0 | 1 | 1 | 8 | 0 | 126 |
| 478801 | *Kytococcus* | *sedentarius* | UP000006666 | 2531 | 1 | 3 | 0 | 2 | 1 | 2 | 0 | 72 |
| 479430 | *Actinospica* | *robiniae* | UP000019485 | 191 | 0 | 0 | 0 | 0 | 0 | 1 | 0 | 4 |
| 479431 | *Nakamurella* | *multipartita* | UP000002218 | 5123 | 1 | 4 | 0 | 1 | 1 | 7 | 0 | 109 |
| 479432 | *Streptosporangium* | *roseum* | UP000002029 | 8955 | 1 | 2 | 0 | 2 | 1 | 26 | 1 | 234 |
| 479433 | *Catenulispora* | *acidiphila* | UP000000851 | 8909 | 1 | 2 | 0 | 2 | 1 | 29 | 1 | 194 |
| 479434 | *Sphaerobacter* | *thermophilus* | UP000002027 | 3471 | 0 | 0 | 0 | 0 | 1 | 2 | 1 | 113 |
| 479435 | *Kribbella* | *flavida* | UP000007967 | 6940 | 1 | 1 | 2 | 2 | 1 | 26 | 1 | 156 |
| 479436 | *Veillonella* | *parvula* | UP000007968 | 1843 | 1 | 0 | 1 | 3 | 1 | 0 | 1 | 81 |
| 479437 | *Eggerthella* | *lenta* | UP000001377 | 3054 | 1 | 1 | 0 | 2 | 1 | 1 | 0 | 120 |
| 481448 | *Methylacidiphilum* | *infernorum* | UP000009149 | 2470 | 1 | 1 | 0 | 1 | 1 | 0 | 0 | 50 |
| 262768 | *Onion* | *yellows* | UP000002523 | 730 | 0 | 0 | 0 | 0 | 0 | 0 | 0 | 11 |
| 483547 | *Geoalkalibacter* | *subterraneus* | UP000035036 | 2987 | 0 | 0 | 1 | 0 | 1 | 1 | 1 | 150 |
| 485447 | *Thalassomonas* | *actiniarum* | UP000032568 | 5460 | 0 | 0 | 1 | 1 | 1 | 0 | 0 | 280 |
| 485913 | *Ktedonobacter* | *racemifer* | UP000004508 | 11211 | 1 | 1 | 0 | 1 | 1 | 13 | 0 | 217 |
| 485915 | *Desulfohalobium* | *retbaense* | UP000001052 | 2500 | 0 | 0 | 1 | 0 | 1 | 1 | 1 | 138 |
| 485917 | *Pedobacter* | *heparinus* | UP000000852 | 4249 | 1 | 1 | 1 | 2 | 1 | 4 | 0 | 115 |
| 485918 | *Chitinophaga* | *pinensis* | UP000002215 | 7179 | 2 | 1 | 1 | 2 | 1 | 1 | 0 | 181 |
| 488538 | *Puniceispirillum* | *marinum* | UP000007460 | 2545 | 0 | 1 | 1 | 1 | 0 | 1 | 1 | 91 |
| 489825 | *Moorea* | *producens* | UP000003959 | 7382 | 1 | 1 | 2 | 1 | 1 | 1 | 0 | 198 |
| 493475 | *Paraglaciecola* | *arctica* | UP000006327 | 5330 | 1 | 1 | 0 | 2 | 1 | 1 | 0 | 214 |
| 497964 | *Chthoniobacter* | *flavus* | UP000005824 | 6705 | 1 | 2 | 1 | 0 | 1 | 3 | 0 | 167 |
| 498211 | *Cellvibrio* | *japonicus* | UP000001036 | 3711 | 1 | 1 | 0 | 1 | 1 | 2 | 1 | 235 |
| 498761 | *Heliobacterium* | *modesticaldum* | UP000008550 | 2922 | 1 | 1 | 1 | 2 | 1 | 0 | 1 | 94 |
| 499207 | *Syntrophaceticus* | *schinkii* | UP000046155 | 3167 | 0 | 0 | 1 | 1 | 1 | 2 | 1 | 83 |
| 500635 | *Mitsuokella* | *multacida* | UP000003671 | 2558 | 1 | 0 | 1 | 2 | 1 | 3 | 1 | 93 |
| 502025 | *Haliangium* | *ochraceum* | UP000001880 | 6684 | 1 | 1 | 1 | 2 | 1 | 2 | 1 | 251 |
| 504472 | *Spirosoma* | *linguale* | UP000002028 | 6867 | 1 | 2 | 1 | 3 | 1 | 6 | 0 | 148 |
| 504487 | *Jejuia* | *pallidilutea* | UP000030184 | 3326 | 1 | 2 | 1 | 1 | 1 | 0 | 0 | 72 |
| 504832 | *Oligotropha* | *carboxidovorans* | UP000007730 | 3624 | 1 | 1 | 1 | 2 | 0 | 0 | 0 | 150 |
| 505317 | *Chelonobacter* | *oris* | UP000030380 | 2284 | 1 | 1 | 1 | 1 | 1 | 5 | 1 | 121 |
| 510955 | *Chryseobacterium* | *solincola* | UP000031275 | 2087 | 1 | 1 | 1 | 1 | 1 | 1 | 0 | 60 |
| 511051 | *Caldisericum* | *exile* | UP000004793 | 1566 | 0 | 0 | 1 | 0 | 1 | 2 | 0 | 43 |
| 511995 | *Azobacteroides* | *pseudotrichonymphae* | UP000000723 | 847 | 0 | 0 | 1 | 0 | 1 | 1 | 0 | 21 |
| 512565 | *Actinoplanes* | *missouriensis* | UP000007882 | 8113 | 1 | 4 | 1 | 2 | 1 | 20 | 0 | 221 |
| 515618 | *Riesia* | *pediculicola* | UP000001700 | 540 | 0 | 0 | 0 | 0 | 1 | 0 | 2 | 12 |
| 515619 | *Agathobacter* | *rectalis* | UP000001477 | 3545 | 1 | 1 | 0 | 0 | 1 | 3 | 0 | 93 |
| 515620 | *Eubacterium* | *eligens* | UP000001476 | 2761 | 1 | 0 | 0 | 0 | 1 | 2 | 0 | 101 |
| 515622 | *Butyrivibrio* | *proteoclasticus* | UP000001299 | 3799 | 1 | 0 | 1 | 1 | 1 | 4 | 0 | 157 |
| 515635 | *Dictyoglomus* | *turgidum* | UP000007719 | 1743 | 0 | 0 | 1 | 0 | 1 | 6 | 1 | 73 |
| 517418 | *Chloroherpeton* | *thalassium* | UP000001208 | 2708 | 2 | 1 | 1 | 2 | 1 | 2 | 1 | 82 |
| 518637 | *Holdemanella* | *biformis* | UP000004315 | 2528 | 0 | 0 | 0 | 0 | 1 | 2 | 0 | 60 |
| 518766 | *Rhodothermus* | *marinus* | UP000002221 | 2861 | 1 | 0 | 1 | 2 | 1 | 3 | 1 | 94 |
| 519441 | *Streptobacillus* | *moniliformis* | UP000002072 | 1431 | 0 | 0 | 0 | 0 | 1 | 4 | 3 | 38 |
| 521003 | *Collinsella* | *intestinalis* | UP000003295 | 1783 | 1 | 0 | 0 | 1 | 1 | 2 | 1 | 46 |
| 521045 | *Kosmotoga* | *olearia* | UP000002382 | 2087 | 0 | 0 | 1 | 0 | 1 | 4 | 0 | 55 |
| 521095 | *Atopobium* | *parvulum* | UP000000960 | 1353 | 0 | 0 | 0 | 1 | 1 | 2 | 1 | 50 |
| 521096 | *Tsukamurella* | *paurometabola* | UP000001213 | 4170 | 1 | 2 | 0 | 2 | 1 | 4 | 0 | 132 |
| 521097 | *Capnocytophaga* | *ochracea* | UP000006650 | 2171 | 1 | 0 | 1 | 1 | 1 | 1 | 0 | 67 |
| 521098 | *Alicyclobacillus* | *acidocaldarius* | UP000001917 | 3059 | 1 | 0 | 1 | 1 | 1 | 3 | 2 | 108 |
| 521674 | *Planctopirus* | *limnophila* | UP000002220 | 4258 | 1 | 1 | 1 | 1 | 1 | 2 | 0 | 130 |
| 522306 | *Accumulibacter* | *phosphatis* | UP000001619 | 4438 | 1 | 5 | 1 | 2 | 1 | 1 | 0 | 247 |
| 522373 | *Stenotrophomonas* | *maltophilia* | UP000008840 | 4365 | 1 | 1 | 1 | 1 | 0 | 1 | 1 | 220 |
| 522772 | *Denitrovibrio* | *acetiphilus* | UP000002012 | 2901 | 1 | 1 | 1 | 3 | 1 | 1 | 1 | 137 |
| 523794 | *Leptotrichia* | *buccalis* | UP000001910 | 2218 | 0 | 0 | 0 | 0 | 1 | 2 | 1 | 67 |
| 525284 | *Gardnerella* | *vaginalis* | UP000001453 | 1365 | 1 | 0 | 0 | 1 | 1 | 7 | 0 | 41 |
| 525371 | *Roseomonas* | *cervicalis* | UP000005324 | 4774 | 1 | 1 | 0 | 2 | 0 | 1 | 1 | 186 |
| 525373 | *Sphingobacterium* | *spiritivorum* | UP000006258 | 4471 | 1 | 1 | 1 | 1 | 1 | 4 | 0 | 98 |
| 525897 | *Desulfomicrobium* | *baculatum* | UP000002216 | 3421 | 0 | 0 | 1 | 0 | 1 | 0 | 1 | 198 |
| 525898 | *Sulfurospirillum* | *deleyianum* | UP000002222 | 2243 | 1 | 1 | 1 | 2 | 1 | 0 | 1 | 158 |
| 525903 | *Thermanaerovibrio* | *acidaminovorans* | UP000002030 | 1737 | 1 | 1 | 1 | 2 | 1 | 1 | 1 | 78 |
| 525904 | *Thermobaculum* | *terrenum* | UP000000323 | 2827 | 0 | 0 | 0 | 0 | 1 | 6 | 0 | 82 |
| 525909 | *Acidimicrobium* | *ferrooxidans* | UP000000771 | 1935 | 1 | 1 | 0 | 1 | 1 | 1 | 1 | 50 |
| 525919 | *Anaerococcus* | *prevotii* | UP000002294 | 1795 | 1 | 1 | 0 | 1 | 1 | 3 | 1 | 61 |
| 526218 | *Sebaldella* | *termitidis* | UP000000845 | 4124 | 1 | 1 | 1 | 1 | 1 | 8 | 1 | 92 |
| 526225 | *Geodermatophilus* | *obscurus* | UP000001382 | 4795 | 1 | 2 | 0 | 2 | 1 | 4 | 0 | 122 |
| 526226 | *Gordonia* | *bronchialis* | UP000001219 | 4628 | 1 | 3 | 0 | 2 | 1 | 3 | 0 | 133 |
| 526227 | *Meiothermus* | *silvanus* | UP000001916 | 3383 | 0 | 1 | 1 | 1 | 0 | 3 | 0 | 92 |
| 530564 | *Pirellula* | *staleyi* | UP000001887 | 4711 | 1 | 1 | 2 | 1 | 1 | 5 | 0 | 137 |
| 545696 | *Holdemania* | *filiformis* | UP000005950 | 4221 | 0 | 0 | 0 | 0 | 1 | 1 | 1 | 88 |
| 546269 | *Filifactor* | *alocis* | UP000007468 | 1616 | 0 | 0 | 0 | 2 | 1 | 0 | 0 | 44 |
| 546270 | *Gemella* | *haemolysans* | UP000006004 | 1709 | 0 | 0 | 0 | 0 | 1 | 1 | 0 | 74 |
| 546274 | *Eikenella* | *corrodens* | UP000005837 | 2627 | 1 | 1 | 1 | 1 | 1 | 0 | 1 | 94 |
| 546414 | *Deinococcus* | *deserti* | UP000002208 | 3459 | 1 | 1 | 1 | 1 | 0 | 6 | 0 | 74 |
| 550540 | *Ferrimonas* | *balearica* | UP000006683 | 3781 | 1 | 1 | 1 | 2 | 1 | 1 | 1 | 203 |
| 552518 | *Elstera* | *litoralis* | UP000033774 | 3298 | 1 | 1 | 1 | 2 | 0 | 1 | 1 | 103 |
| 552811 | *Dehalogenimonas* | *lykanthroporepellens* | UP000002349 | 1619 | 0 | 0 | 1 | 0 | 1 | 1 | 0 | 39 |
| 553217 | *Enhydrobacter* | *aerosaccus* | UP000010294 | 2573 | 1 | 2 | 0 | 1 | 1 | 0 | 1 | 110 |
| 555079 | *Thermosediminibacter* | *oceani* | UP000000272 | 2151 | 0 | 0 | 1 | 1 | 1 | 1 | 1 | 97 |
| 555088 | *Dethiobacter* | *alkaliphilus* | UP000006443 | 3162 | 0 | 0 | 1 | 1 | 1 | 1 | 2 | 97 |
| 555500 | *Galbibacter* | *marinus* | UP000007364 | 3099 | 1 | 1 | 1 | 1 | 1 | 1 | 0 | 71 |
| 555778 | *Halothiobacillus* | *neapolitanus* | UP000009102 | 2353 | 1 | 2 | 1 | 1 | 1 | 1 | 0 | 157 |
| 555779 | *Desulfonatronospira* | *thiodismutans* | UP000005496 | 3577 | 0 | 0 | 1 | 0 | 1 | 1 | 2 | 149 |
| 556269 | *Oxalobacter* | *formigenes* | UP000005089 | 2076 | 1 | 1 | 1 | 1 | 1 | 0 | 0 | 116 |
| 557598 | *Laribacter* | *hongkongensis* | UP000002010 | 3208 | 1 | 2 | 1 | 1 | 1 | 1 | 0 | 192 |
| 562729 | *Rheinheimera* | *nanhaiensis* | UP000004374 | 3730 | 1 | 0 | 1 | 2 | 1 | 0 | 0 | 197 |
| 562970 | *Kyrpidia* | *tusciae* | UP000002368 | 3135 | 1 | 0 | 0 | 1 | 1 | 2 | 1 | 127 |
| 563192 | *Bilophila* | *wadsworthia* | UP000006034 | 3779 | 1 | 1 | 1 | 1 | 1 | 0 | 2 | 183 |
| 565034 | *Brachyspira* | *hyodysenteriae* | UP000001803 | 2642 | 0 | 0 | 0 | 0 | 1 | 1 | 1 | 75 |
| 565045 | *Luminiphilus* | *syltensis* | UP000004699 | 2923 | 1 | 0 | 0 | 1 | 1 | 0 | 1 | 114 |
| 566550 | *Hungatella* | *hathewayi* | UP000004968 | 7571 | 0 | 1 | 0 | 1 | 1 | 14 | 0 | 153 |
| 570509 | *Spiroplasma* | *melliferum* | UP000004057 | 1154 | 0 | 0 | 0 | 0 | 1 | 1 | 0 | 18 |
| 572265 | *Hamiltonella* | *defensa* | UP000002334 | 2045 | 0 | 0 | 0 | 0 | 1 | 0 | 1 | 136 |
| 572477 | *Allochromatium* | *vinosum* | UP000001441 | 3211 | 1 | 2 | 1 | 1 | 1 | 0 | 1 | 221 |
| 572479 | *Halanaerobium* | *praevalens* | UP000006866 | 2055 | 0 | 0 | 0 | 1 | 1 | 1 | 1 | 116 |
| 572480 | *Arcobacter* | *nitrofigilis* | UP000000939 | 3125 | 0 | 2 | 1 | 2 | 1 | 0 | 0 | 165 |
| 572544 | *Ilyobacter* | *polytropus* | UP000006875 | 2859 | 1 | 1 | 1 | 2 | 1 | 1 | 1 | 99 |
| 572547 | *Aminobacterium* | *colombiense* | UP000002366 | 1872 | 0 | 0 | 1 | 1 | 1 | 0 | 1 | 58 |
| 573065 | *Asticcacaulis* | *excentricus* | UP000001492 | 3743 | 1 | 1 | 1 | 2 | 0 | 3 | 1 | 125 |
| 357244 | *Orientia* | *tsutsugamushi* | UP000001565 | 966 | 0 | 0 | 0 | 0 | 0 | 0 | 1 | 20 |
| 573413 | *Spirochaeta* | *smaragdinae* | UP000002318 | 4211 | 1 | 0 | 1 | 0 | 1 | 16 | 0 | 153 |
| 574087 | *Acetohalobium* | *arabaticum* | UP000001661 | 2276 | 0 | 0 | 1 | 1 | 1 | 1 | 1 | 101 |
| 575540 | *Isosphaera* | *pallida* | UP000008631 | 3721 | 1 | 0 | 2 | 0 | 1 | 3 | 0 | 113 |
| 575590 | *Bacteroidetes* | *oral* | UP000004685 | 1854 | 0 | 0 | 1 | 0 | 1 | 1 | 0 | 59 |
| 577650 | *Desulfobulbus* | *propionicus* | UP000006365 | 3259 | 1 | 2 | 1 | 0 | 1 | 0 | 1 | 180 |
| 580332 | *Sideroxydans* | *lithotrophicus* | UP000001625 | 2977 | 1 | 1 | 1 | 1 | 1 | 1 | 0 | 197 |
| 580340 | *Thermovirga* | *lienii* | UP000005868 | 1853 | 0 | 0 | 1 | 1 | 1 | 0 | 2 | 81 |
| 582402 | *Hirschia* | *baltica* | UP000002745 | 3187 | 1 | 1 | 1 | 2 | 1 | 1 | 1 | 108 |
| 582515 | *Rubidibacter* | *lacunae* | UP000016960 | 3455 | 1 | 2 | 2 | 1 | 1 | 1 | 0 | 118 |
| 582744 | *Methylovorus* | *glucosetrophus* | UP000002743 | 2907 | 1 | 1 | 1 | 1 | 1 | 1 | 0 | 226 |
| 583355 | *Coraliomargarita* | *akajimensis* | UP000000925 | 3110 | 1 | 2 | 1 | 1 | 1 | 2 | 1 | 79 |
| 584708 | *Aminomonas* | *paucivorans* | UP000005096 | 2391 | 1 | 1 | 1 | 2 | 1 | 1 | 1 | 111 |
| 585394 | *Roseburia* | *hominis* | UP000008178 | 3351 | 1 | 1 | 0 | 1 | 1 | 3 | 0 | 108 |
| 585501 | *Oribacterium* | *sinus* | UP000004121 | 2638 | 1 | 0 | 0 | 0 | 1 | 3 | 0 | 83 |
| 585503 | *Selenomonas* | *noxia* | UP000003701 | 2020 | 0 | 0 | 1 | 1 | 1 | 0 | 1 | 99 |
| 585506 | *Weissella* | *paramesenteroides* | UP000004528 | 1952 | 1 | 0 | 0 | 2 | 1 | 3 | 1 | 58 |
| 585530 | *Brevibacterium* | *mcbrellneri* | UP000005714 | 2432 | 0 | 2 | 0 | 2 | 1 | 1 | 0 | 63 |
| 585531 | *Aeromicrobium* | *marinum* | UP000003111 | 3077 | 1 | 2 | 0 | 2 | 1 | 2 | 0 | 73 |
| 586416 | *Terribacillus* | *aidingensis* | UP000027980 | 3230 | 1 | 0 | 1 | 1 | 1 | 4 | 2 | 132 |
| 589865 | *Desulfurivibrio* | *alkaliphilus* | UP000001508 | 2595 | 0 | 0 | 1 | 0 | 1 | 0 | 2 | 163 |
| 591001 | *Acidaminococcus* | *fermentans* | UP000001902 | 2016 | 1 | 1 | 0 | 2 | 1 | 1 | 1 | 76 |
| 592010 | *Abiotrophia* | *defectiva* | UP000019050 | 1943 | 0 | 0 | 0 | 0 | 1 | 6 | 1 | 85 |
| 592026 | *Catonella* | *morbi* | UP000018227 | 3288 | 0 | 0 | 0 | 0 | 1 | 5 | 1 | 128 |
| 592029 | *Nonlabens* | *dokdonensis* | UP000011173 | 3613 | 2 | 1 | 1 | 1 | 1 | 0 | 0 | 79 |
| 593907 | *Cellulomonas* | *gilvus* | UP000000485 | 3153 | 1 | 2 | 0 | 1 | 1 | 8 | 0 | 106 |
| 595494 | *Tolumonas* | *auensis* | UP000009073 | 3126 | 1 | 0 | 1 | 2 | 1 | 7 | 1 | 171 |
| 596154 | *Alicycliphilus* | *denitrificans* | UP000007938 | 4620 | 1 | 2 | 1 | 1 | 1 | 1 | 0 | 220 |
| 596315 | *Peptostreptococcus* | *stomatis* | UP000003244 | 1600 | 0 | 0 | 0 | 0 | 1 | 1 | 0 | 73 |
| 598659 | *Nautilia* | *profundicola* | UP000000448 | 1730 | 0 | 3 | 1 | 2 | 1 | 0 | 1 | 109 |
| 608538 | *Hydrogenobacter* | *thermophilus* | UP000002574 | 1892 | 0 | 0 | 1 | 1 | 1 | 1 | 1 | 68 |
| 626369 | *Granulicatella* | *elegans* | UP000002939 | 1562 | 0 | 0 | 0 | 0 | 1 | 3 | 1 | 73 |
| 626522 | *Alloprevotella* | *tannerae* | UP000003460 | 2806 | 0 | 0 | 1 | 0 | 1 | 1 | 1 | 54 |
| 626523 | *Shuttleworthia* | *satelles* | UP000003494 | 2247 | 0 | 0 | 0 | 0 | 1 | 3 | 0 | 63 |
| 626939 | *Phascolarctobacterium* | *succinatutens* | UP000004923 | 2148 | 1 | 1 | 0 | 1 | 1 | 0 | 1 | 70 |
| 629741 | *Kingella* | *oralis* | UP000003009 | 3160 | 1 | 1 | 1 | 1 | 1 | 0 | 1 | 95 |
| 630626 | *Shimwellia* | *blattae* | UP000001955 | 3870 | 2 | 0 | 1 | 3 | 1 | 6 | 1 | 206 |
| 631454 | *Lutibaculum* | *baratangense* | UP000017819 | 4164 | 1 | 1 | 1 | 2 | 0 | 1 | 0 | 161 |
| 633147 | *Olsenella* | *uli* | UP000000333 | 1739 | 1 | 0 | 0 | 1 | 1 | 2 | 1 | 74 |
| 633149 | *Brevundimonas* | *subvibrioides* | UP000002696 | 3326 | 1 | 1 | 1 | 2 | 0 | 3 | 1 | 115 |
| 634452 | *Acetobacter* | *pasteurianus* | UP000000948 | 2906 | 1 | 2 | 0 | 2 | 0 | 1 | 1 | 92 |
| 634503 | *Edwardsiella* | *ictaluri* | UP000001485 | 3686 | 1 | 0 | 1 | 2 | 1 | 4 | 1 | 217 |
| 635013 | *Thermincola* | *potens* | UP000002377 | 2908 | 0 | 0 | 1 | 1 | 1 | 1 | 1 | 109 |
| 638303 | *Thermocrinis* | *albus* | UP000002043 | 1592 | 0 | 0 | 1 | 1 | 1 | 1 | 1 | 76 |
| 639282 | *Deferribacter* | *desulfuricans* | UP000001520 | 2338 | 0 | 0 | 1 | 1 | 1 | 1 | 1 | 120 |
| 639283 | *Starkeya* | *novella* | UP000006633 | 4424 | 1 | 3 | 1 | 2 | 1 | 0 | 0 | 180 |
| 640081 | *Dechlorosoma* | *suillum* | UP000005633 | 3432 | 1 | 2 | 1 | 1 | 1 | 1 | 0 | 247 |
| 640132 | *Segniliparus* | *rotundus* | UP000002247 | 3001 | 1 | 1 | 0 | 2 | 1 | 1 | 0 | 93 |
| 641146 | *Scardovia* | *inopinata* | UP000005777 | 1482 | 1 | 0 | 0 | 1 | 1 | 4 | 0 | 45 |
| 641147 | *Simonsiella* | *muelleri* | UP000017813 | 2220 | 1 | 1 | 1 | 1 | 1 | 0 | 0 | 96 |
| 641526 | *Winogradskyella* | *psychrotolerans* | UP000014962 | 4023 | 1 | 1 | 1 | 1 | 1 | 0 | 0 | 79 |
| 642492 | *Cellulosilyticum* | *lentocellum* | UP000008467 | 4150 | 0 | 0 | 0 | 0 | 1 | 4 | 1 | 142 |
| 643562 | *Desulfovibrio* | *aespoeensis* | UP000002191 | 3269 | 0 | 0 | 1 | 0 | 1 | 0 | 1 | 159 |
| 643648 | *Syntrophothermus* | *lipocalidus* | UP000000378 | 2311 | 0 | 0 | 1 | 1 | 1 | 1 | 1 | 94 |
| 643867 | *Marivirga* | *tractuosa* | UP000008720 | 3748 | 1 | 1 | 1 | 2 | 1 | 3 | 2 | 80 |
| 644282 | *Desulfarculus* | *baarsii* | UP000009047 | 3268 | 0 | 0 | 1 | 1 | 1 | 1 | 1 | 181 |
| 644283 | *Micromonospora* | *aurantiaca* | UP000001908 | 6204 | 1 | 2 | 1 | 2 | 1 | 18 | 0 | 177 |
| 644284 | *Arcanobacterium* | *haemolyticum* | UP000000376 | 1717 | 0 | 0 | 0 | 1 | 1 | 5 | 0 | 61 |
| 644966 | *Thermaerobacter* | *marianensis* | UP000008915 | 2324 | 0 | 0 | 1 | 0 | 1 | 3 | 1 | 92 |
| 645991 | *Syntrophobotulus* | *glycolicus* | UP000007488 | 3105 | 1 | 1 | 1 | 2 | 1 | 1 | 1 | 142 |
| 646529 | *Desulfosporosinus* | *acidiphilus* | UP000002892 | 4469 | 0 | 0 | 1 | 0 | 1 | 3 | 0 | 170 |
| 648757 | *Rhodomicrobium* | *vannielii* | UP000001399 | 3513 | 1 | 1 | 0 | 2 | 0 | 1 | 0 | 146 |
| 648996 | *Thermovibrio* | *ammonificans* | UP000006362 | 1812 | 1 | 0 | 1 | 2 | 1 | 1 | 0 | 78 |
| 649349 | *Leadbetterella* | *byssophila* | UP000007435 | 3429 | 1 | 0 | 1 | 2 | 1 | 3 | 0 | 81 |
| 649638 | *Truepera* | *radiovictrix* | UP000000379 | 2924 | 1 | 1 | 1 | 0 | 1 | 3 | 0 | 93 |
| 649756 | *Anaerostipes* | *hadrus* | UP000008960 | 2771 | 2 | 0 | 0 | 0 | 1 | 3 | 0 | 77 |
| 650150 | *Erysipelothrix* | *rhusiopathiae* | UP000007944 | 1679 | 0 | 0 | 0 | 0 | 0 | 5 | 1 | 70 |
| 651182 | *Desulfobacula* | *toluolica* | UP000007347 | 4189 | 0 | 0 | 1 | 0 | 1 | 0 | 1 | 232 |
| 651822 | *Fretibacterium* | *fastidiosum* | UP000008957 | 1434 | 0 | 0 | 0 | 1 | 1 | 1 | 1 | 53 |
| 653733 | *Desulfurispirillum* | *indicum* | UP000002572 | 2551 | 1 | 1 | 1 | 2 | 1 | 1 | 2 | 151 |
| 655812 | *Aerococcus* | *viridans* | UP000003764 | 1929 | 1 | 1 | 0 | 1 | 1 | 3 | 0 | 69 |
| 655815 | *Zunongwangia* | *profunda* | UP000001654 | 4633 | 1 | 0 | 1 | 2 | 1 | 3 | 0 | 93 |
| 657308 | *Gordonibacter* | *pamelaeae* | UP000008805 | 2027 | 0 | 1 | 0 | 2 | 0 | 0 | 0 | 64 |
| 657314 | *Blautia* | *obeum* | UP000008955 | 3155 | 1 | 1 | 0 | 1 | 1 | 2 | 1 | 79 |
| 657316 | *Megamonas* | *hypermegale* | UP000008806 | 2118 | 0 | 0 | 1 | 2 | 1 | 1 | 1 | 52 |
| 660470 | *Mesotoga* | *prima* | UP000002881 | 2499 | 0 | 0 | 1 | 0 | 1 | 8 | 1 | 65 |
| 661478 | *Fimbriimonas* | *ginsengisoli* | UP000027982 | 4817 | 1 | 1 | 1 | 2 | 1 | 3 | 1 | 96 |
| 663278 | *Ethanoligenens* | *harbinense* | UP000001551 | 2663 | 1 | 1 | 0 | 1 | 1 | 4 | 1 | 126 |
| 665126 | *Prosthecomicrobium* | *hirschii* | UP000048984 | 5501 | 1 | 2 | 1 | 2 | 1 | 1 | 0 | 197 |
| 666509 | *Planktomarina* | *temperata* | UP000028680 | 3024 | 1 | 0 | 1 | 2 | 0 | 3 | 1 | 100 |
| 666681 | *Methylotenera* | *versatilis* | UP000000383 | 2752 | 1 | 1 | 1 | 1 | 1 | 1 | 0 | 198 |
| 667014 | *Thermodesulfatator* | *indicus* | UP000006793 | 2184 | 0 | 0 | 1 | 1 | 1 | 0 | 1 | 107 |
| 668336 | *Aggregatibacter* | *actinomycetemcomitans* | UP000002569 | 2276 | 0 | 0 | 1 | 1 | 1 | 3 | 0 | 128 |
| 670307 | *Hyphomicrobium* | *denitrificans* | UP000005952 | 3832 | 1 | 3 | 1 | 2 | 0 | 0 | 0 | 125 |
| 670487 | *Oceanithermus* | *profundus* | UP000008722 | 2372 | 0 | 1 | 1 | 1 | 0 | 1 | 0 | 62 |
| 675635 | *Pseudonocardia* | *dioxanivorans* | UP000007809 | 6620 | 1 | 4 | 0 | 2 | 1 | 5 | 0 | 130 |
| 679192 | *Bulleidia* | *extructa* | UP000005017 | 1415 | 0 | 0 | 0 | 0 | 0 | 1 | 0 | 49 |
| 679200 | *Johnsonella* | *ignava* | UP000003011 | 2284 | 1 | 0 | 0 | 1 | 1 | 3 | 1 | 83 |
| 681645 | *Corynebacterium* | *pseudotuberculosis* | UP000000276 | 2090 | 0 | 2 | 0 | 2 | 1 | 3 | 0 | 72 |
| 686340 | *Methylomicrobium* | *album* | UP000005090 | 3738 | 1 | 1 | 1 | 1 | 1 | 0 | 0 | 232 |
| 688245 | *Comamonas* | *testosteroni* | UP000002360 | 4780 | 1 | 1 | 1 | 1 | 1 | 0 | 0 | 251 |
| 690566 | *Sphingobium* | *chlorophenolicum* | UP000007150 | 4062 | 1 | 1 | 1 | 2 | 0 | 1 | 0 | 142 |
| 693746 | *Oscillibacter* | *valericigenes* | UP000005219 | 4593 | 0 | 0 | 0 | 0 | 1 | 3 | 0 | 105 |
| 694427 | *Paludibacter* | *propionicigenes* | UP000008718 | 3020 | 1 | 0 | 1 | 1 | 1 | 1 | 0 | 80 |
| 694431 | *Desulfurella* | *acetivorans* | UP000019023 | 1825 | 1 | 1 | 1 | 2 | 1 | 1 | 1 | 105 |
| 482235 | *Phytoplasma* | *mali* | UP000002020 | 448 | 0 | 0 | 0 | 0 | 0 | 0 | 0 | 15 |
| 696747 | *Arthrospira* | *platensis* | UP000006803 | 6009 | 1 | 1 | 2 | 1 | 1 | 1 | 0 | 124 |
| 697281 | *Mahella* | *australiensis* | UP000008457 | 2858 | 0 | 0 | 1 | 1 | 1 | 10 | 1 | 99 |
| 697282 | *Methylobacter* | *tundripaludum* | UP000004664 | 4194 | 1 | 2 | 1 | 1 | 1 | 0 | 0 | 274 |
| 698738 | *Oleispira* | *antarctica* | UP000032749 | 3895 | 1 | 0 | 1 | 1 | 1 | 0 | 0 | 204 |
| 698758 | *Amphibacillus* | *xylanus* | UP000006294 | 2384 | 1 | 0 | 0 | 1 | 1 | 6 | 2 | 97 |
| 699246 | *Mageeibacillus* | *indolicus* | UP000008234 | 1566 | 0 | 0 | 0 | 0 | 1 | 1 | 0 | 62 |
| 700015 | *Coriobacterium* | *glomerans* | UP000006851 | 1768 | 1 | 0 | 0 | 1 | 1 | 8 | 1 | 62 |
| 700598 | *Niastella* | *koreensis* | UP000005438 | 7171 | 3 | 1 | 1 | 1 | 1 | 5 | 0 | 156 |
| 701347 | *Enterobacter* | *lignolyticus* | UP000006872 | 4393 | 1 | 1 | 1 | 2 | 1 | 6 | 1 | 312 |
| 702450 | *Turicibacter* | *sanguinis* | UP000002938 | 2779 | 1 | 0 | 1 | 1 | 1 | 7 | 0 | 89 |
| 706191 | *Pantoea* | *ananatis* | UP000001702 | 4241 | 1 | 1 | 1 | 2 | 1 | 4 | 1 | 231 |
| 706433 | *Solobacterium* | *moorei* | UP000004097 | 2034 | 0 | 0 | 0 | 0 | 1 | 2 | 0 | 70 |
| 706434 | *Megasphaera* | *micronuciformis* | UP000003195 | 1771 | 1 | 0 | 1 | 2 | 1 | 0 | 1 | 58 |
| 706587 | *Desulfomonile* | *tiedjei* | UP000006055 | 5412 | 0 | 0 | 1 | 0 | 1 | 0 | 2 | 232 |
| 709032 | *Sulfuricurvum* | *kujiense* | UP000008721 | 2794 | 1 | 1 | 1 | 2 | 1 | 1 | 1 | 178 |
| 709991 | *Odoribacter* | *splanchnicus* | UP000006657 | 3479 | 1 | 0 | 0 | 1 | 1 | 3 | 0 | 108 |
| 710696 | *Intrasporangium* | *calvum* | UP000008914 | 3551 | 1 | 1 | 0 | 2 | 1 | 3 | 1 | 102 |
| 713604 | *Amycolatopsis* | *mediterranei* | UP000006138 | 9551 | 1 | 1 | 1 | 3 | 2 | 23 | 0 | 210 |
| 713887 | *Atelocyanobacterium* | *thalassa* | UP000001405 | 1199 | 1 | 0 | 0 | 0 | 1 | 1 | 0 | 43 |
| 714943 | *Mucilaginibacter* | *paludis* | UP000002774 | 6864 | 2 | 1 | 1 | 2 | 1 | 3 | 0 | 131 |
| 714995 | *Komagataeibacter* | *hansenii* | UP000006468 | 3303 | 1 | 2 | 0 | 2 | 1 | 6 | 1 | 127 |
| 716544 | *Waddlia* | *chondrophila* | UP000001505 | 1919 | 0 | 0 | 1 | 0 | 1 | 0 | 0 | 70 |
| 717231 | *Flexistipes* | *sinusarabici* | UP000006621 | 2185 | 0 | 0 | 1 | 1 | 1 | 1 | 1 | 84 |
| 717605 | *Thermobacillus* | *composti* | UP000010795 | 3802 | 0 | 0 | 0 | 0 | 1 | 5 | 2 | 127 |
| 717772 | *Thioalkalimicrobium* | *aerophilum* | UP000005380 | 2067 | 1 | 1 | 1 | 1 | 1 | 1 | 1 | 137 |
| 717774 | *Marinomonas* | *mediterranea* | UP000001062 | 4118 | 1 | 1 | 1 | 1 | 1 | 1 | 0 | 268 |
| 717960 | *Faecalitalea* | *cylindroides* | UP000008801 | 1442 | 1 | 0 | 0 | 0 | 0 | 0 | 0 | 38 |
| 717962 | *Coprococcus* | *catus* | UP000008798 | 2985 | 1 | 1 | 0 | 1 | 1 | 1 | 0 | 96 |
| 742159 | *Achromobacter* | *piechaudii* | UP000004510 | 5755 | 1 | 2 | 1 | 1 | 0 | 0 | 0 | 290 |
| 742725 | *Alistipes* | *indistinctus* | UP000006008 | 2348 | 1 | 0 | 1 | 1 | 1 | 3 | 0 | 71 |
| 742743 | *Dialister* | *succinatiphilus* | UP000003277 | 2140 | 1 | 2 | 1 | 3 | 1 | 1 | 0 | 68 |
| 742766 | *Dysgonomonas* | *gadei* | UP000004913 | 4153 | 1 | 1 | 1 | 2 | 1 | 6 | 0 | 87 |
| 743718 | *Isoptericola* | *variabilis* | UP000009236 | 2879 | 1 | 1 | 1 | 2 | 1 | 7 | 0 | 76 |
| 744872 | *Treponema* | *caldarium* | UP000000503 | 2754 | 1 | 0 | 1 | 1 | 1 | 2 | 0 | 99 |
| 745277 | *Rahnella* | *aquatilis* | UP000009010 | 4841 | 1 | 0 | 1 | 2 | 1 | 8 | 0 | 334 |
| 745411 | *Gallaecimonas* | *xiamenensis* | UP000006755 | 3798 | 1 | 1 | 1 | 2 | 1 | 2 | 1 | 231 |
| 746697 | *Aequorivita* | *sublithincola* | UP000006049 | 3134 | 1 | 1 | 1 | 1 | 1 | 0 | 1 | 83 |
| 747365 | *Thermodesulfobium* | *narugense* | UP000011765 | 1797 | 1 | 1 | 1 | 2 | 0 | 1 | 0 | 76 |
| 748449 | *Halobacteroides* | *halobius* | UP000010880 | 2452 | 0 | 0 | 1 | 1 | 1 | 2 | 1 | 109 |
| 749219 | *Moraxella* | *catarrhalis* | UP000000930 | 1881 | 1 | 1 | 0 | 2 | 1 | 0 | 0 | 57 |
| 749222 | *Nitratifractor* | *salsuginis* | UP000008633 | 2076 | 1 | 2 | 1 | 2 | 1 | 1 | 1 | 87 |
| 749414 | *Streptomyces* | *bingchenggensis* | UP000000377 | 10019 | 1 | 1 | 0 | 3 | 1 | 25 | 2 | 308 |
| 755178 | *Cyanobacterium* | *aponinum* | UP000010480 | 3415 | 1 | 1 | 2 | 1 | 1 | 1 | 1 | 128 |
| 755732 | *Fluviicola* | *taffensis* | UP000007463 | 4016 | 1 | 0 | 1 | 2 | 1 | 1 | 0 | 94 |
| 756272 | *Rubinisphaera* | *brasiliensis* | UP000006860 | 4710 | 1 | 1 | 1 | 1 | 1 | 2 | 0 | 144 |
| 757424 | *Herbaspirillum* | *seropedicae* | UP000000329 | 4732 | 1 | 2 | 1 | 1 | 1 | 0 | 0 | 312 |
| 760142 | *Hippea* | *maritima* | UP000008139 | 1653 | 0 | 0 | 0 | 1 | 1 | 1 | 1 | 89 |
| 760192 | *Haliscomenobacter* | *hydrossis* | UP000008461 | 6704 | 2 | 1 | 1 | 1 | 1 | 8 | 0 | 133 |
| 761193 | *Runella* | *slithyformis* | UP000000493 | 5750 | 1 | 1 | 1 | 2 | 1 | 7 | 0 | 124 |
| 762903 | *Pseudopedobacter* | *saltans* | UP000000310 | 3780 | 1 | 0 | 1 | 2 | 1 | 3 | 0 | 86 |
| 762948 | *Rothia* | *dentocariosa* | UP000000387 | 2212 | 1 | 1 | 0 | 2 | 1 | 1 | 0 | 62 |
| 762967 | *Sutterella* | *parvirubra* | UP000004956 | 2483 | 1 | 1 | 1 | 1 | 1 | 0 | 0 | 67 |
| 762983 | *Succinatimonas* | *hippei* | UP000018458 | 2169 | 1 | 0 | 0 | 0 | 1 | 2 | 1 | 69 |
| 765910 | *Marichromatium* | *purpuratum* | UP000005275 | 3249 | 1 | 1 | 1 | 1 | 1 | 0 | 1 | 216 |
| 765911 | *Thiocystis* | *violascens* | UP000006062 | 4192 | 1 | 3 | 1 | 1 | 1 | 0 | 1 | 248 |
| 765912 | *Thioflavicoccus* | *mobilis* | UP000010816 | 3528 | 1 | 1 | 1 | 1 | 1 | 0 | 0 | 174 |
| 765913 | *Thiorhodococcus* | *drewsii* | UP000004200 | 4731 | 1 | 3 | 1 | 1 | 1 | 1 | 0 | 314 |
| 765914 | *Thiorhodospira* | *sibirica* | UP000003058 | 2933 | 1 | 1 | 1 | 1 | 1 | 0 | 0 | 178 |
| 765952 | *Parachlamydia* | *acanthamoebae* | UP000000495 | 2784 | 0 | 0 | 1 | 0 | 1 | 1 | 0 | 90 |
| 767434 | *Frateuria* | *aurantia* | UP000005234 | 3098 | 1 | 1 | 1 | 1 | 1 | 0 | 1 | 198 |
| 768066 | *Halomonas* | *elongata* | UP000008707 | 3469 | 1 | 0 | 1 | 1 | 1 | 1 | 0 | 213 |
| 768670 | *Calditerrivibrio* | *nitroreducens* | UP000007039 | 2089 | 1 | 1 | 1 | 2 | 1 | 1 | 1 | 120 |
| 768671 | *Thiocapsa* | *marina* | UP000005459 | 4915 | 1 | 3 | 1 | 1 | 1 | 0 | 0 | 227 |
| 795359 | *Thermodesulfobacterium* | *geofontis* | UP000006583 | 1594 | 0 | 0 | 1 | 1 | 1 | 0 | 1 | 82 |
| 796942 | *Stomatobaculum* | *longum* | UP000018466 | 2070 | 1 | 1 | 0 | 1 | 1 | 1 | 0 | 64 |
| 797473 | *Cardiobacterium* | *valvarum* | UP000004750 | 3026 | 0 | 1 | 1 | 1 | 1 | 0 | 0 | 88 |
| 856793 | *Micavibrio* | *aeruginosavorus* | UP000009286 | 2432 | 0 | 0 | 0 | 0 | 1 | 1 | 1 | 106 |
| 857087 | *Methylomonas* | *methanica* | UP000008888 | 4435 | 1 | 2 | 1 | 1 | 1 | 1 | 0 | 273 |
| 857265 | *Amantichitinum* | *ursilacus* | UP000037939 | 4368 | 1 | 0 | 1 | 1 | 1 | 2 | 0 | 278 |
| 857293 | *Caloramator* | *australicus* | UP000007652 | 2679 | 0 | 0 | 1 | 0 | 1 | 3 | 2 | 92 |
| 861299 | *Gemmatirosa* | *kalamazoonesis* | UP000019151 | 6261 | 1 | 1 | 1 | 1 | 1 | 6 | 0 | 226 |
| 861450 | *Anaeroglobus* | *geminatus* | UP000005481 | 2148 | 1 | 0 | 1 | 1 | 1 | 0 | 1 | 57 |
| 862517 | *Peptoniphilus* | *duerdenii* | UP000003280 | 1987 | 1 | 0 | 0 | 1 | 1 | 0 | 0 | 71 |
| 862719 | *Azospirillum* | *lipoferum* | UP000005667 | 6062 | 1 | 1 | 1 | 2 | 0 | 3 | 1 | 274 |
| 862908 | *Halobacteriovorax* | *marinus* | UP000008963 | 3230 | 0 | 0 | 1 | 2 | 1 | 0 | 1 | 101 |
| 864069 | *Microvirga* | *lotononidis* | UP000003947 | 6918 | 1 | 2 | 1 | 2 | 1 | 6 | 0 | 237 |
| 864564 | *Parascardovia* | *denticolens* | UP000004946 | 1679 | 1 | 0 | 0 | 1 | 1 | 3 | 0 | 51 |
| 864702 | *Oscillatoriales* | *cyanobacterium* | UP000001332 | 4673 | 1 | 1 | 1 | 1 | 1 | 1 | 0 | 138 |
| 865937 | *Gillisia* | *limnaea* | UP000003844 | 3411 | 1 | 1 | 1 | 1 | 1 | 2 | 1 | 90 |
| 865938 | *Weeksella* | *virosa* | UP000008641 | 2045 | 1 | 1 | 1 | 1 | 1 | 0 | 0 | 60 |
| 866499 | *Cloacibacillus* | *evryensis* | UP000023044 | 1082 | 1 | 0 | 0 | 1 | 0 | 0 | 0 | 31 |
| 866536 | *Belliella* | *baltica* | UP000006050 | 3609 | 1 | 0 | 1 | 2 | 1 | 2 | 0 | 73 |
| 866895 | *Halobacillus* | *halophilus* | UP000007397 | 4100 | 1 | 0 | 0 | 1 | 1 | 4 | 3 | 148 |
| 867900 | *Cellulophaga* | *lytica* | UP000007487 | 3281 | 1 | 1 | 1 | 1 | 1 | 1 | 1 | 71 |
| 867902 | *Ornithobacterium* | *rhinotracheale* | UP000006051 | 2138 | 1 | 1 | 1 | 1 | 1 | 2 | 0 | 54 |
| 868864 | *Desulfurobacterium* | *thermolithotrophum* | UP000007102 | 1496 | 1 | 0 | 1 | 2 | 1 | 0 | 0 | 73 |
| 869210 | *Marinithermus* | *hydrothermalis* | UP000007030 | 2194 | 0 | 0 | 1 | 0 | 0 | 1 | 0 | 71 |
| 869212 | *Turneriella* | *parva* | UP000006048 | 4092 | 1 | 1 | 1 | 1 | 0 | 2 | 0 | 99 |
| 869213 | *Saccharicrinis* | *fermentans* | UP000019402 | 4663 | 1 | 1 | 1 | 1 | 1 | 3 | 0 | 107 |
| 869279 | *Thermanaerothrix* | *daxensis* | UP000050544 | 2394 | 0 | 1 | 1 | 0 | 0 | 7 | 1 | 89 |
| 870187 | *Thiothrix* | *nivea* | UP000005317 | 4218 | 1 | 3 | 1 | 1 | 1 | 1 | 1 | 179 |
| 272947 | *Rickettsia* | *prowazekii* | UP000002480 | 834 | 0 | 0 | 0 | 0 | 0 | 0 | 1 | 42 |
| 871571 | *Mobiluncus* | *mulieris* | UP000003045 | 2379 | 1 | 1 | 0 | 1 | 1 | 7 | 0 | 65 |
| 872965 | *Ardenticatena* | *maritima* | UP000037784 | 3144 | 0 | 0 | 1 | 0 | 1 | 3 | 1 | 79 |
| 879212 | *Desulfobacter* | *postgatei* | UP000005778 | 3396 | 1 | 1 | 1 | 1 | 1 | 0 | 1 | 163 |
| 880070 | *Cyclobacterium* | *marinum* | UP000001635 | 4983 | 1 | 1 | 1 | 2 | 1 | 5 | 0 | 102 |
| 880071 | *Flexibacter* | *litoralis* | UP000006054 | 3832 | 1 | 1 | 1 | 1 | 1 | 0 | 0 | 79 |
| 880072 | *Desulfobacca* | *acetoxidans* | UP000000483 | 2859 | 0 | 0 | 1 | 0 | 1 | 1 | 1 | 121 |
| 880073 | *Caldithrix* | *abyssi* | UP000004671 | 3674 | 0 | 0 | 1 | 1 | 1 | 6 | 1 | 162 |
| 880074 | *Barnesiella* | *viscericola* | UP000018901 | 2487 | 1 | 0 | 1 | 1 | 1 | 1 | 0 | 68 |
| 880591 | *Ketogulonicigenium* | *vulgare* | UP000006871 | 3209 | 1 | 0 | 1 | 1 | 0 | 2 | 0 | 143 |
| 883066 | *Actinobaculum* | *massiliense* | UP000009888 | 1696 | 1 | 1 | 0 | 1 | 1 | 4 | 0 | 51 |
| 883081 | *Alloiococcus* | *otitis* | UP000009875 | 1627 | 1 | 0 | 0 | 1 | 1 | 2 | 0 | 60 |
| 883096 | *Bergeyella* | *zoohelcum* | UP000006085 | 2120 | 1 | 0 | 1 | 1 | 1 | 0 | 0 | 63 |
| 883103 | *Dolosigranulum* | *pigrum* | UP000003599 | 1691 | 0 | 0 | 0 | 0 | 1 | 4 | 0 | 67 |
| 883111 | *Facklamia* | *hominis* | UP000004465 | 1717 | 1 | 0 | 1 | 1 | 1 | 2 | 1 | 75 |
| 883114 | *Helcococcus* | *kunzii* | UP000004191 | 1876 | 1 | 1 | 0 | 1 | 1 | 7 | 1 | 82 |
| 883126 | *Massilia* | *timonae* | UP000009874 | 5096 | 1 | 1 | 1 | 2 | 1 | 1 | 0 | 309 |
| 883161 | *Propionimicrobium* | *lymphophilum* | UP000014417 | 2076 | 1 | 0 | 0 | 1 | 1 | 2 | 0 | 63 |
| 883169 | *Turicella* | *otitidis* | UP000006078 | 1846 | 0 | 2 | 0 | 2 | 1 | 2 | 0 | 59 |
| 885272 | *Jonquetella* | *anthropi* | UP000003806 | 1508 | 0 | 0 | 1 | 1 | 1 | 0 | 2 | 55 |
| 886293 | *Singulisphaera* | *acidiphila* | UP000010798 | 7126 | 1 | 2 | 2 | 0 | 1 | 5 | 0 | 240 |
| 886377 | *Muricauda* | *ruestringensis* | UP000008908 | 3425 | 1 | 1 | 1 | 0 | 1 | 3 | 0 | 67 |
| 887062 | *Hylemonella* | *gracilis* | UP000016368 | 3342 | 0 | 0 | 1 | 1 | 1 | 0 | 0 | 210 |
| 887898 | *Lautropia* | *mirabilis* | UP000011021 | 2665 | 1 | 1 | 1 | 1 | 1 | 0 | 1 | 174 |
| 887929 | *Pseudoramibacter* | *alactolyticus* | UP000004754 | 2514 | 0 | 0 | 1 | 1 | 1 | 1 | 0 | 86 |
| 889306 | *Jeotgalibacillus* | *soli* | UP000031938 | 3938 | 1 | 1 | 0 | 1 | 1 | 4 | 3 | 147 |
| 891968 | *Anaerobaculum* | *mobile* | UP000006061 | 2004 | 0 | 0 | 1 | 1 | 1 | 1 | 2 | 77 |
| 891974 | *Plautia* | *stali* | UP000016901 | 5007 | 1 | 0 | 0 | 2 | 1 | 6 | 0 | 186 |
| 444179 | *Sulcia* | *muelleri* | UP000000781 | 227 | 0 | 0 | 0 | 0 | 0 | 0 | 0 | 4 |
| 908337 | *Eremococcus* | *coleocola* | UP000005990 | 1720 | 1 | 0 | 0 | 1 | 1 | 2 | 0 | 64 |
| 909613 | *Actinokineospora* | *spheciospongiae* | UP000019277 | 6610 | 1 | 2 | 1 | 3 | 1 | 8 | 0 | 209 |
| 910954 | *Dietzia* | *cinnamea* | UP000004165 | 3537 | 1 | 2 | 0 | 2 | 1 | 1 | 0 | 82 |
| 914150 | *Kangiella* | *geojedonensis* | UP000034071 | 2207 | 1 | 1 | 1 | 1 | 1 | 0 | 0 | 103 |
| 926550 | *Caldilinea* | *aerophila* | UP000007880 | 4097 | 1 | 1 | 1 | 1 | 1 | 5 | 1 | 121 |
| 926556 | *Echinicola* | *vietnamensis* | UP000010796 | 4509 | 1 | 1 | 1 | 3 | 1 | 6 | 0 | 97 |
| 926559 | *Joostella* | *marina* | UP000004690 | 3783 | 1 | 1 | 1 | 1 | 1 | 0 | 0 | 73 |
| 926562 | *Owenweeksia* | *hongkongensis* | UP000005631 | 3471 | 1 | 1 | 1 | 2 | 1 | 0 | 0 | 83 |
| 926566 | *Terriglobus* | *roseus* | UP000006056 | 3936 | 1 | 1 | 0 | 1 | 1 | 4 | 1 | 154 |
| 926569 | *Anaerolinea* | *thermophila* | UP000008922 | 3110 | 0 | 1 | 1 | 0 | 1 | 6 | 1 | 112 |
| 927083 | *Sandaracinus* | *amylolyticus* | UP000034883 | 8941 | 1 | 2 | 1 | 3 | 0 | 1 | 1 | 274 |
| 927661 | *Cryptosporangium* | *arvum* | UP000021053 | 4033 | 0 | 1 | 0 | 1 | 1 | 12 | 0 | 126 |
| 927668 | *Pseudanabaena* | *biceps* | UP000011201 | 4751 | 1 | 1 | 2 | 1 | 1 | 0 | 0 | 136 |
| 929556 | *Solitalea* | *canadensis* | UP000007590 | 4304 | 2 | 2 | 1 | 2 | 1 | 4 | 0 | 99 |
| 929562 | *Emticicia* | *oligotrophica* | UP000002875 | 4251 | 1 | 1 | 1 | 2 | 1 | 5 | 0 | 106 |
| 929563 | *Leptonema* | *illini* | UP000005737 | 4129 | 1 | 2 | 1 | 1 | 0 | 2 | 1 | 138 |
| 929704 | *Myroides* | *odoratus* | UP000005785 | 3687 | 1 | 1 | 1 | 1 | 1 | 0 | 0 | 95 |
| 929713 | *Niabella* | *soli* | UP000003586 | 4110 | 2 | 1 | 1 | 1 | 1 | 6 | 0 | 99 |
| 930169 | *Alcanivorax* | *dieselolei* | UP000006286 | 4388 | 1 | 1 | 2 | 1 | 1 | 0 | 1 | 249 |
| 931626 | *Acetobacterium* | *woodii* | UP000007177 | 3445 | 1 | 1 | 0 | 3 | 1 | 1 | 0 | 183 |
| 935700 | *Jannaschia* | *aquimarina* | UP000032232 | 4049 | 1 | 2 | 1 | 2 | 0 | 1 | 1 | 123 |
| 937774 | *Taylorella* | *equigenitalis* | UP000007472 | 1556 | 0 | 1 | 1 | 1 | 1 | 0 | 0 | 98 |
| 940190 | *Melissococcus* | *plutonius* | UP000008456 | 1876 | 0 | 0 | 0 | 0 | 1 | 4 | 0 | 74 |
| 941824 | *Thermobrachium* | *celere* | UP000014923 | 2370 | 0 | 0 | 1 | 0 | 1 | 2 | 2 | 104 |
| 945021 | *Tetragenococcus* | *halophilus* | UP000002663 | 2507 | 0 | 0 | 0 | 0 | 1 | 4 | 0 | 87 |
| 945713 | *Ignavibacterium* | *album* | UP000007394 | 3169 | 1 | 1 | 1 | 1 | 1 | 2 | 1 | 116 |
| 946077 | *Imtechella* | *halotolerans* | UP000005938 | 2694 | 1 | 1 | 1 | 1 | 1 | 0 | 1 | 58 |
| 948458 | *Actinotalea* | *ferrariae* | UP000019753 | 3558 | 1 | 1 | 0 | 1 | 1 | 6 | 0 | 100 |
| 981222 | *Chloracidobacterium* | *thermophilum* | UP000006791 | 3053 | 0 | 0 | 1 | 1 | 1 | 2 | 1 | 132 |
| 983917 | *Rubrivivax* | *gelatinosus* | UP000007883 | 4686 | 1 | 2 | 1 | 1 | 1 | 0 | 0 | 258 |
| 983920 | *Novosphingobium* | *nitrogenifigens* | UP000004728 | 3800 | 1 | 1 | 0 | 2 | 1 | 1 | 1 | 153 |
| 984262 | *Saprospira* | *grandis* | UP000007519 | 4218 | 1 | 0 | 1 | 0 | 1 | 0 | 0 | 67 |
| 986075 | *Caldalkalibacillus* | *thermarum* | UP000010716 | 2969 | 0 | 0 | 0 | 0 | 1 | 1 | 1 | 95 |
| 988812 | *Marinovum* | *algicola* | UP000036352 | 4976 | 1 | 2 | 1 | 2 | 0 | 2 | 1 | 171 |
| 991905 | *Polymorphum* | *gilvum* | UP000008130 | 4354 | 1 | 3 | 1 | 2 | 1 | 2 | 0 | 160 |
| 994573 | *Youngiibacter* | *fragilis* | UP000017747 | 3736 | 1 | 1 | 0 | 2 | 1 | 5 | 1 | 121 |
| 999415 | *Eggerthia* | *catenaformis* | UP000011758 | 1874 | 0 | 0 | 0 | 0 | 1 | 3 | 0 | 71 |
| 999552 | *Leisingera* | *methylohalidivorans* | UP000018780 | 4508 | 0 | 2 | 1 | 2 | 0 | 0 | 1 | 141 |
| 1000565 | *Methyloversatilis* | *universalis* | UP000005019 | 3919 | 1 | 1 | 1 | 1 | 1 | 1 | 0 | 243 |
| 1001240 | *Cryobacterium* | *roopkundense* | UP000029864 | 3249 | 1 | 0 | 0 | 1 | 1 | 8 | 0 | 108 |
| 1002809 | *Solibacillus* | *silvestris* | UP000006691 | 3812 | 1 | 1 | 0 | 1 | 1 | 2 | 1 | 127 |
| 1003237 | *Nitrospirillum* | *amazonense* | UP000018435 | 3357 | 1 | 0 | 0 | 1 | 0 | 1 | 0 | 152 |
| 1005048 | *Collimonas* | *fungivorans* | UP000008392 | 4429 | 1 | 1 | 1 | 1 | 0 | 0 | 0 | 287 |
| 1005058 | *Gallibacterium* | *anatis* | UP000006908 | 2482 | 0 | 0 | 1 | 1 | 1 | 4 | 0 | 110 |
| 1005995 | *Tatumella* | *ptyseos* | UP000028602 | 3337 | 1 | 0 | 1 | 2 | 1 | 3 | 1 | 204 |
| 1005999 | *Leminorella* | *grimontii* | UP000028624 | 3756 | 1 | 0 | 1 | 2 | 1 | 3 | 1 | 239 |
| 1006576 | *Defluviitoga* | *tunisiensis* | UP000032809 | 1811 | 0 | 0 | 1 | 0 | 1 | 7 | 0 | 72 |
| 1009370 | *Acetonema* | *longum* | UP000003240 | 4283 | 1 | 0 | 1 | 2 | 1 | 1 | 3 | 198 |
| 1026882 | *Methylophaga* | *aminisulfidivorans* | UP000003544 | 3174 | 1 | 0 | 1 | 1 | 1 | 0 | 0 | 209 |
| 1027292 | *Sporosarcina* | *newyorkensis* | UP000005316 | 3825 | 0 | 0 | 0 | 0 | 1 | 0 | 1 | 142 |
| 1032480 | *Microlunatus* | *phosphovorus* | UP000007947 | 5305 | 1 | 2 | 0 | 1 | 1 | 5 | 0 | 123 |
| 1033802 | *Salinisphaera* | *shabanensis* | UP000006242 | 3524 | 1 | 0 | 1 | 1 | 1 | 1 | 0 | 199 |
| 1033810 | *Haloplasma* | *contractile* | UP000005707 | 3019 | 0 | 0 | 1 | 0 | 1 | 3 | 2 | 86 |
| 1036672 | *Advenella* | *kashmirensis* | UP000005267 | 3933 | 0 | 2 | 1 | 1 | 1 | 0 | 0 | 136 |
| 1045855 | *Pseudoxanthomonas* | *spadix* | UP000005870 | 3147 | 1 | 1 | 1 | 1 | 0 | 0 | 1 | 155 |
| 1046627 | *Bizionia* | *argentinensis* | UP000003730 | 3095 | 1 | 1 | 1 | 1 | 1 | 0 | 1 | 79 |
| 1048260 | *Leptospirillum* | *ferriphilum* | UP000006177 | 2450 | 0 | 1 | 1 | 2 | 1 | 0 | 1 | 118 |
| 1048983 | *Anditalea* | *andensis* | UP000027821 | 4341 | 1 | 0 | 1 | 3 | 1 | 5 | 1 | 93 |
| 1051632 | *Sulfobacillus* | *acidophilus* | UP000000291 | 3722 | 0 | 0 | 1 | 0 | 1 | 5 | 1 | 82 |
| 1072685 | *Basilea* | *psittacipulmonis* | UP000028945 | 1452 | 0 | 1 | 1 | 1 | 1 | 0 | 0 | 82 |
| 1076588 | *Thiolapillus* | *brandeum* | UP000031631 | 2824 | 0 | 2 | 1 | 1 | 1 | 1 | 1 | 176 |
| 1082931 | *Pelagibacterium* | *halotolerans* | UP000008850 | 3875 | 1 | 1 | 1 | 2 | 1 | 5 | 0 | 153 |
| 1082933 | *Mesorhizobium* | *amorphae* | UP000002949 | 7083 | 1 | 2 | 1 | 2 | 0 | 8 | 0 | 293 |
| 1085623 | *Glaciecola* | *nitratireducens* | UP000009282 | 3653 | 1 | 1 | 0 | 2 | 1 | 1 | 1 | 191 |
| 1086011 | *Flavobacterium* | *frigoris* | UP000005566 | 3590 | 1 | 1 | 1 | 1 | 1 | 1 | 0 | 85 |
| 1088868 | *Commensalibacter* | *intestini* | UP000005939 | 2209 | 1 | 1 | 1 | 2 | 0 | 0 | 1 | 95 |
| 1089455 | *Mobilicoccus* | *pelagius* | UP000004367 | 3112 | 1 | 3 | 0 | 2 | 1 | 2 | 0 | 103 |
| 1089553 | *Thermacetogenium* | *phaeum* | UP000000467 | 2803 | 0 | 0 | 1 | 1 | 2 | 1 | 1 | 100 |
| 1094508 | *Thermoanaerobacterium* | *saccharolyticum* | UP000006178 | 2778 | 0 | 0 | 1 | 1 | 1 | 6 | 1 | 92 |
| 1097667 | *Patulibacter* | *medicamentivorans* | UP000005143 | 4372 | 1 | 1 | 1 | 2 | 1 | 3 | 1 | 113 |
| 1105367 | *Paenirhodobacter* | *enshiensis* | UP000028824 | 2781 | 1 | 2 | 1 | 2 | 0 | 0 | 1 | 115 |
| 1110502 | *Tistrella* | *mobilis* | UP000005258 | 5778 | 1 | 1 | 1 | 2 | 0 | 1 | 1 | 214 |
| 273119 | *Ureaplasma* | *parvum* | UP000000423 | 611 | 0 | 0 | 0 | 0 | 0 | 0 | 0 | 14 |
| 1116472 | *Methyloglobulus* | *morosus* | UP000017842 | 3737 | 1 | 1 | 1 | 1 | 1 | 0 | 0 | 181 |
| 1117647 | *Simiduia* | *agarivorans* | UP000000466 | 3812 | 1 | 0 | 0 | 1 | 1 | 2 | 1 | 221 |
| 1120923 | *Acidocella* | *aminolytica* | UP000032668 | 3751 | 1 | 1 | 0 | 2 | 0 | 3 | 1 | 180 |
| 1121014 | *Arenimonas* | *donghaensis* | UP000029085 | 2685 | 1 | 1 | 1 | 1 | 1 | 0 | 1 | 130 |
| 1121126 | *Brochothrix* | *thermosphacta* | UP000019242 | 2589 | 0 | 0 | 0 | 1 | 1 | 4 | 1 | 87 |
| 1121877 | *Ferrimicrobium* | *acidiphilum* | UP000032336 | 2974 | 1 | 1 | 0 | 1 | 1 | 1 | 1 | 68 |
| 1123057 | *Rhodonellum* | *psychrophilum* | UP000016843 | 4904 | 1 | 1 | 1 | 3 | 1 | 4 | 0 | 100 |
| 1123069 | *Rubellimicrobium* | *thermophilum* | UP000015346 | 3244 | 1 | 0 | 1 | 1 | 1 | 3 | 1 | 108 |
| 1123237 | *Salipiger* | *mucosus* | UP000015347 | 5583 | 1 | 2 | 1 | 2 | 0 | 2 | 1 | 183 |
| 1123360 | *Litoreibacter* | *arenae* | UP000015351 | 3640 | 1 | 2 | 1 | 2 | 1 | 2 | 1 | 101 |
| 1123366 | *Thalassospira* | *xiamenensis* | UP000007127 | 4340 | 1 | 1 | 1 | 2 | 0 | 3 | 1 | 188 |
| 1123367 | *Thauera* | *linaloolentis* | UP000013232 | 3836 | 1 | 3 | 1 | 1 | 1 | 0 | 0 | 265 |
| 1123501 | *Wenxinia* | *marina* | UP000035100 | 4030 | 1 | 1 | 1 | 2 | 0 | 3 | 1 | 124 |
| 1124991 | *Morganella* | *morganii* | UP000011834 | 3510 | 1 | 0 | 1 | 2 | 1 | 5 | 1 | 222 |
| 1125718 | *Actinomyces* | *massiliensis* | UP000002941 | 3129 | 1 | 1 | 0 | 1 | 1 | 5 | 0 | 83 |
| 1127673 | *Aliiglaciecola* | *lipolytica* | UP000006334 | 4327 | 1 | 0 | 1 | 2 | 1 | 0 | 0 | 180 |
| 1129897 | *Nitrolancea* | *hollandica* | UP000004221 | 3969 | 0 | 0 | 0 | 0 | 1 | 2 | 0 | 75 |
| 1131935 | *Paenibacillus* | *dendritiformis* | UP000003900 | 5660 | 0 | 0 | 0 | 0 | 1 | 10 | 2 | 229 |
| 1133849 | *Nocardia* | *brasiliensis* | UP000006304 | 8414 | 1 | 1 | 0 | 2 | 1 | 12 | 0 | 267 |
| 1134406 | *Ornatilinea* | *apprima* | UP000050417 | 3344 | 1 | 2 | 2 | 0 | 1 | 10 | 0 | 102 |
| 1141663 | *Providencia* | *rettgeri* | UP000009338 | 3847 | 0 | 0 | 1 | 2 | 1 | 3 | 1 | 267 |
| 1142394 | *Phycisphaera* | *mikurensis* | UP000007881 | 3269 | 1 | 1 | 1 | 0 | 1 | 1 | 1 | 92 |
| 1144275 | *Corallococcus* | *coralloides* | UP000007587 | 8019 | 1 | 2 | 1 | 2 | 1 | 3 | 0 | 315 |
| 1144889 | *Modestobacter* | *marinus* | UP000006461 | 5435 | 1 | 3 | 0 | 2 | 1 | 6 | 0 | 115 |
| 1145276 | *Lysinibacillus* | *varians* | UP000019675 | 4504 | 1 | 1 | 0 | 1 | 1 | 1 | 1 | 156 |
| 1146883 | *Blastococcus* | *saxobsidens* | UP000007517 | 4791 | 1 | 1 | 0 | 2 | 1 | 4 | 0 | 129 |
| 1149862 | *Pelosinus* | *fermentans* | UP000004324 | 4690 | 1 | 0 | 1 | 1 | 1 | 3 | 1 | 241 |
| 1150469 | *Pararhodospirillum* | *photometricum* | UP000033220 | 3275 | 1 | 1 | 1 | 2 | 0 | 1 | 1 | 143 |
| 1150600 | *Arcticibacter* | *svalbardensis* | UP000014174 | 4309 | 2 | 1 | 1 | 2 | 1 | 0 | 0 | 101 |
| 1150626 | *Phaeospirillum* | *molischianum* | UP000004169 | 3771 | 1 | 1 | 1 | 2 | 0 | 0 | 1 | 189 |
| 1156417 | *Caloranaerobacter* | *azorensis* | UP000029622 | 2148 | 0 | 0 | 0 | 0 | 1 | 3 | 2 | 86 |
| 1157490 | *Tumebacillus* | *flagellatus* | UP000027931 | 4452 | 1 | 0 | 1 | 1 | 1 | 1 | 1 | 168 |
| 1160710 | *Microbacterium* | *laevaniformans* | UP000004547 | 3249 | 1 | 2 | 0 | 1 | 1 | 6 | 0 | 84 |
| 1163407 | *Rhodanobacter* | *spathiphylli* | UP000003226 | 3438 | 1 | 1 | 1 | 1 | 0 | 0 | 0 | 177 |
| 1163617 | *Sulfuricella* | *denitrificans* | UP000015559 | 3071 | 1 | 2 | 1 | 1 | 1 | 1 | 0 | 221 |
| 1164990 | *Richelia* | *intracellularis* | UP000019767 | 7610 | 1 | 0 | 1 | 1 | 1 | 1 | 0 | 71 |
| 1166018 | *Fibrella* | *aestuarina* | UP000011058 | 5627 | 1 | 2 | 1 | 3 | 1 | 8 | 0 | 125 |
| 1167006 | *Desulfocapsa* | *sulfexigens* | UP000011721 | 3514 | 0 | 3 | 1 | 1 | 1 | 0 | 1 | 180 |
| 1168034 | *Draconibacterium* | *orientale* | UP000023772 | 3940 | 1 | 1 | 1 | 1 | 1 | 6 | 0 | 95 |
| 1171373 | *Propionibacterium* | *acidipropionici* | UP000000214 | 3316 | 1 | 1 | 0 | 1 | 1 | 3 | 0 | 71 |
| 1171377 | *Bibersteinia* | *trehalosi* | UP000011846 | 2244 | 0 | 0 | 1 | 1 | 1 | 2 | 1 | 129 |
| 1172194 | *Hydrocarboniphaga* | *effusa* | UP000003704 | 4474 | 0 | 1 | 1 | 1 | 1 | 2 | 1 | 220 |
| 1173020 | *Chamaesiphon* | *minutus* | UP000010366 | 5815 | 1 | 3 | 1 | 1 | 1 | 1 | 0 | 168 |
| 1173022 | *Crinalium* | *epipsammum* | UP000010472 | 4697 | 1 | 1 | 2 | 1 | 1 | 1 | 0 | 133 |
| 1179773 | *Saccharothrix* | *espanaensis* | UP000006281 | 8423 | 1 | 3 | 1 | 4 | 1 | 15 | 0 | 234 |
| 1184607 | *Austwickia* | *chelonae* | UP000008495 | 3048 | 1 | 1 | 0 | 2 | 1 | 7 | 0 | 132 |
| 1184609 | *Kineosphaera* | *limosa* | UP000008366 | 4422 | 1 | 4 | 0 | 2 | 1 | 4 | 0 | 131 |
| 1185766 | *Thioclava* | *dalianensis* | UP000027725 | 4007 | 1 | 1 | 1 | 2 | 0 | 2 | 1 | 135 |
| 1185876 | *Fibrisoma* | *limi* | UP000009309 | 6341 | 1 | 1 | 1 | 2 | 1 | 6 | 0 | 132 |
| 1188319 | *Ferriphaselus* | *amnicola* | UP000033070 | 2638 | 1 | 0 | 1 | 2 | 1 | 0 | 0 | 190 |
| 1189611 | *Nitratireductor* | *aquibiodomus* | UP000004622 | 4240 | 1 | 1 | 1 | 2 | 0 | 3 | 0 | 153 |
| 1189612 | *Indibacter* | *alkaliphilus* | UP000006073 | 4650 | 1 | 0 | 1 | 3 | 1 | 3 | 0 | 87 |
| 1189621 | *Nitritalea* | *halalkaliphila* | UP000005551 | 3102 | 0 | 0 | 1 | 2 | 0 | 3 | 0 | 58 |
| 1191523 | *Melioribacter* | *roseus* | UP000009011 | 2833 | 1 | 0 | 1 | 2 | 1 | 4 | 1 | 110 |
| 1192034 | *Chondromyces* | *apiculatus* | UP000019678 | 9037 | 0 | 1 | 1 | 2 | 0 | 2 | 1 | 370 |
| 1193181 | *Tetrasphaera* | *elongata* | UP000013167 | 3089 | 1 | 3 | 0 | 1 | 1 | 3 | 1 | 77 |
| 1195246 | *Alishewanella* | *agri* | UP000035062 | 3152 | 1 | 1 | 1 | 2 | 1 | 0 | 0 | 172 |
| 1196095 | *Gilliamella* | *apicola* | UP000019656 | 2784 | 1 | 0 | 1 | 1 | 1 | 2 | 1 | 137 |
| 1197477 | *Mangrovimonas* | *yunxiaonensis* | UP000028521 | 2307 | 1 | 1 | 1 | 1 | 1 | 0 | 0 | 61 |
| 1198114 | *Granulicella* | *tundricola* | UP000000343 | 4514 | 1 | 2 | 0 | 1 | 1 | 2 | 1 | 143 |
| 1203606 | *Butyricicoccus* | *pullicaecorum* | UP000013981 | 3107 | 1 | 0 | 0 | 0 | 1 | 4 | 0 | 67 |
| 1207063 | *Oceanibaculum* | *indicum* | UP000006746 | 3754 | 1 | 1 | 1 | 2 | 1 | 1 | 1 | 156 |
| 1208323 | *Celeribacter* | *baekdonensis* | UP000006762 | 4198 | 0 | 3 | 1 | 2 | 0 | 2 | 1 | 139 |
| 1209989 | *Tepidanaerobacter* | *acetatoxydans* | UP000010802 | 2640 | 0 | 0 | 1 | 1 | 1 | 6 | 1 | 79 |
| 1210046 | *Janibacter* | *hoylei* | UP000004474 | 3070 | 1 | 2 | 0 | 2 | 1 | 3 | 0 | 74 |
| 1215343 | *Liberibacter* | *crescens* | UP000010799 | 1376 | 1 | 0 | 0 | 2 | 1 | 0 | 0 | 63 |
| 1223802 | *Sulfuritalea* | *hydrogenivorans* | UP000031637 | 3578 | 1 | 2 | 1 | 1 | 1 | 1 | 0 | 228 |
| 1225176 | *Cecembia* | *lonarensis* | UP000004478 | 4213 | 1 | 2 | 1 | 2 | 1 | 3 | 0 | 104 |
| 1227360 | *Viridibacillus* | *arenosi* | UP000019062 | 4296 | 1 | 1 | 0 | 1 | 1 | 2 | 2 | 160 |
| 1227739 | *Hymenobacter* | *swuensis* | UP000019423 | 4612 | 2 | 1 | 1 | 2 | 1 | 0 | 1 | 121 |
| 1228997 | *Riemerella* | *anatipestifer* | UP000006276 | 2187 | 1 | 2 | 1 | 1 | 1 | 1 | 0 | 54 |
| 1229521 | *Nitrincola* | *nitratireducens* | UP000019464 | 4019 | 1 | 1 | 1 | 1 | 1 | 0 | 1 | 249 |
| 1230341 | *Salimicrobium* | *jeotgali* | UP000011746 | 2730 | 0 | 0 | 0 | 0 | 1 | 3 | 3 | 107 |
| 1231350 | *Acidisphaera* | *rubrifaciens* | UP000032680 | 2788 | 0 | 1 | 1 | 0 | 0 | 0 | 1 | 122 |
| 1231351 | *Acidomonas* | *methanolica* | UP000019760 | 3270 | 1 | 1 | 0 | 2 | 1 | 5 | 1 | 143 |
| 1231392 | *Oceaniovalibus* | *guishaninsula* | UP000006765 | 2840 | 1 | 1 | 1 | 2 | 0 | 1 | 1 | 84 |
| 1231623 | *Tanticharoenia* | *sakaeratensis* | UP000032679 | 3154 | 1 | 1 | 1 | 2 | 1 | 3 | 1 | 135 |
| 1233231 | *Scytonema* | *tolypothrichoides* | UP000031959 | 7078 | 1 | 1 | 2 | 1 | 1 | 2 | 0 | 186 |
| 1234409 | *Catellicoccus* | *marimammalium* | UP000016057 | 1196 | 0 | 0 | 0 | 0 | 1 | 1 | 0 | 50 |
| 1234679 | *Carnobacterium* | *maltaromaticum* | UP000000212 | 3539 | 0 | 0 | 0 | 0 | 1 | 6 | 1 | 109 |
| 1235279 | *Bhargavaea* | *cecembensis* | UP000011919 | 3168 | 0 | 0 | 1 | 0 | 1 | 1 | 1 | 116 |
| 1236989 | *Geofilum* | *rubicundum* | UP000032900 | 4411 | 1 | 1 | 1 | 0 | 1 | 6 | 0 | 109 |
| 1237149 | *Fulvivirga* | *imtechensis* | UP000011135 | 5952 | 2 | 1 | 1 | 2 | 1 | 3 | 1 | 142 |
| 1238182 | *Caenispirillum* | *salinarum* | UP000009881 | 4574 | 1 | 1 | 1 | 2 | 0 | 1 | 1 | 188 |
| 1239962 | *Mariniradius* | *saccharolyticus* | UP000010953 | 4633 | 1 | 2 | 1 | 2 | 1 | 3 | 0 | 84 |
| 1242864 | *Cystobacter* | *fuscus* | UP000011682 | 10511 | 1 | 1 | 1 | 4 | 1 | 5 | 0 | 357 |
| 1260251 | *Spiribacter* | *salinus* | UP000017881 | 1684 | 1 | 0 | 1 | 1 | 1 | 0 | 0 | 70 |
| 1261130 | *Wohlfahrtiimonas* | *chitiniclastica* | UP000011617 | 2004 | 1 | 0 | 0 | 1 | 1 | 0 | 0 | 99 |
| 1263073 | *Dorea* | *formicigenerans* | UP000018109 | 2461 | 2 | 1 | 0 | 0 | 1 | 2 | 0 | 61 |
| 1263095 | *Paraprevotella* | *clara* | UP000017958 | 2847 | 1 | 0 | 1 | 1 | 1 | 1 | 0 | 61 |
| 1263099 | *Parasutterella* | *excrementihominis* | UP000018034 | 2228 | 1 | 2 | 1 | 1 | 1 | 0 | 0 | 61 |
| 1265313 | *Pseudohaliea* | *rubra* | UP000029640 | 2860 | 1 | 1 | 0 | 1 | 1 | 0 | 1 | 130 |
| 1266370 | *Nitrospina* | *gracilis* | UP000011704 | 2962 | 0 | 0 | 1 | 1 | 1 | 0 | 0 | 104 |
| 1267021 | *Frischella* | *perrara* | UP000030901 | 2270 | 1 | 0 | 0 | 1 | 1 | 2 | 1 | 161 |
| 1267766 | *Altererythrobacter* | *atlanticus* | UP000034392 | 3254 | 1 | 2 | 1 | 2 | 1 | 1 | 0 | 113 |
| 1279009 | *Cesiribacter* | *andamanensis* | UP000011910 | 4225 | 0 | 1 | 2 | 2 | 1 | 2 | 0 | 89 |
| 1280514 | *Acidithrix* | *ferrooxidans* | UP000032360 | 3548 | 1 | 1 | 0 | 1 | 1 | 2 | 1 | 77 |
| 1286631 | *Sphaerotilus* | *natans* | UP000026714 | 4177 | 1 | 2 | 1 | 1 | 1 | 1 | 0 | 261 |
| 1286632 | *Zhouia* | *amylolytica* | UP000018850 | 3362 | 1 | 1 | 1 | 1 | 1 | 0 | 0 | 67 |
| 1286635 | *Desulfotignum* | *phosphitoxidans* | UP000014216 | 4515 | 0 | 0 | 1 | 0 | 1 | 0 | 1 | 187 |
| 1292022 | *Curtobacterium* | *flaccumfaciens* | UP000019755 | 3386 | 1 | 2 | 0 | 1 | 1 | 10 | 0 | 98 |
| 1294273 | *Roseibacterium* | *elongatum* | UP000019593 | 3433 | 1 | 2 | 1 | 2 | 0 | 1 | 0 | 95 |
| 1298593 | *Thalassolituus* | *oleivorans* | UP000011866 | 3634 | 1 | 1 | 1 | 1 | 1 | 1 | 1 | 189 |
| 1300341 | *Croceitalea* | *dokdonensis* | UP000050280 | 3682 | 1 | 1 | 1 | 1 | 1 | 1 | 1 | 58 |
| 1303518 | *Chthonomonas* | *calidirosea* | UP000014227 | 2809 | 1 | 1 | 1 | 2 | 1 | 2 | 1 | 109 |
| 1304284 | *Caldisalinibacter* | *kiritimatiensis* | UP000013378 | 2681 | 1 | 0 | 1 | 2 | 1 | 1 | 1 | 94 |
| 1304833 | *Hassallia* | *byssoidea* | UP000031549 | 10137 | 2 | 2 | 2 | 3 | 1 | 4 | 1 | 256 |
| 1307761 | *Salinispira* | *pacifica* | UP000018680 | 3350 | 1 | 0 | 1 | 1 | 1 | 6 | 0 | 106 |
| 1308866 | *Gracilibacillus* | *halophilus* | UP000012283 | 2968 | 1 | 0 | 0 | 1 | 1 | 3 | 2 | 106 |
| 1312852 | *Thermoanaerobaculum* | *aquaticum* | UP000027284 | 2252 | 0 | 0 | 1 | 0 | 1 | 0 | 1 | 107 |
| 1313172 | *Ilumatobacter* | *coccineus* | UP000011863 | 4290 | 1 | 2 | 1 | 0 | 1 | 4 | 0 | 116 |
| 1313304 | *Chitinivibrio* | *alkaliphilus* | UP000017148 | 2304 | 0 | 0 | 0 | 0 | 1 | 1 | 0 | 92 |
| 1315976 | *Plesiomonas* | *shigelloides* | UP000014012 | 3377 | 1 | 1 | 1 | 2 | 1 | 4 | 1 | 222 |
| 1317118 | *Roseivivax* | *atlanticus* | UP000019063 | 4264 | 1 | 0 | 1 | 2 | 1 | 2 | 1 | 139 |
| 1317122 | *Aquimarina* | *atlantica* | UP000023541 | 4786 | 1 | 3 | 1 | 1 | 1 | 1 | 1 | 121 |
| 1319815 | *Cetobacterium* | *somerae* | UP000017081 | 2974 | 2 | 1 | 0 | 2 | 1 | 8 | 0 | 102 |
| 1321816 | *Alloscardovia* | *omnicolens* | UP000016519 | 1649 | 1 | 0 | 0 | 1 | 1 | 6 | 0 | 35 |
| 1328313 | *Catenovulum* | *agarivorans* | UP000019276 | 3835 | 1 | 0 | 1 | 2 | 1 | 0 | 0 | 186 |
| 1330036 | *Osedax* | *symbiont* | UP000014822 | 4383 | 0 | 0 | 1 | 0 | 1 | 0 | 1 | 238 |
| 1330458 | *Agrococcus* | *pavilionensis* | UP000016462 | 2776 | 1 | 1 | 1 | 1 | 1 | 4 | 0 | 83 |
| 1331007 | *Agarivorans* | *albus* | UP000014461 | 4397 | 1 | 1 | 1 | 2 | 1 | 3 | 1 | 222 |
| 1333998 | *Tepidicaulis* | *marinus* | UP000028702 | 3398 | 1 | 1 | 1 | 2 | 1 | 1 | 1 | 123 |
| 1338011 | *Elizabethkingia* | *anophelis* | UP000028933 | 4061 | 1 | 1 | 1 | 1 | 1 | 1 | 0 | 103 |
| 1338436 | *Sinomonas* | *humi* | UP000030982 | 3444 | 1 | 2 | 0 | 2 | 0 | 6 | 0 | 92 |
| 1347342 | *Formosa* | *agariphila* | UP000016160 | 3557 | 1 | 1 | 1 | 1 | 1 | 0 | 0 | 84 |
| 1348774 | *Croceicoccus* | *naphthovorans* | UP000035287 | 3411 | 1 | 2 | 2 | 2 | 0 | 0 | 0 | 89 |
| 1348852 | *Mumia* | *flava* | UP000031002 | 14150 | 2 | 5 | 1 | 4 | 1 | 9 | 1 | 676 |
| 1349421 | *Flavihumibacter* | *solisilvae* | UP000031408 | 4215 | 1 | 2 | 1 | 1 | 1 | 6 | 0 | 109 |
| 1349767 | *Janthinobacterium* | *agaricidamnosum* | UP000027604 | 5488 | 1 | 1 | 1 | 1 | 1 | 2 | 0 | 362 |
| 1379159 | *Dyella* | *jiangningensis* | UP000024387 | 4342 | 1 | 3 | 1 | 1 | 1 | 0 | 0 | 225 |
| 1379858 | *Mucispirillum* | *schaedleri* | UP000017429 | 2091 | 1 | 0 | 1 | 3 | 1 | 1 | 1 | 99 |
| 1382230 | *Asaia* | *platycodi* | UP000027583 | 3273 | 1 | 1 | 1 | 2 | 0 | 2 | 1 | 128 |
| 1382798 | *Tamlana* | *nanhaiensis* | UP000032361 | 3029 | 1 | 1 | 1 | 1 | 1 | 0 | 0 | 68 |
| 1384459 | *Methyloceanibacter* | *caenitepidi* | UP000031643 | 3341 | 1 | 3 | 1 | 2 | 1 | 0 | 0 | 104 |
| 1384484 | *Adlercreutzia* | *equolifaciens* | UP000015924 | 2281 | 1 | 1 | 0 | 2 | 1 | 0 | 0 | 57 |
| 1385369 | *Skermanella* | *stibiiresistens* | UP000019486 | 7269 | 1 | 3 | 1 | 3 | 0 | 6 | 1 | 296 |
| 1385514 | *Pontibacillus* | *yanchengensis* | UP000030147 | 3964 | 1 | 1 | 0 | 1 | 1 | 3 | 3 | 131 |
| 1385518 | *Knoellia* | *flava* | UP000029990 | 3373 | 1 | 2 | 0 | 2 | 1 | 4 | 0 | 75 |
| 1395513 | *Sporolactobacillus* | *laevolacticus* | UP000018296 | 3497 | 1 | 1 | 0 | 2 | 1 | 2 | 0 | 117 |
| 1398085 | *Inquilinus* | *limosus* | UP000029995 | 6228 | 1 | 2 | 1 | 2 | 0 | 6 | 1 | 249 |
| 871271 | *Zinderia* | *insecticola* | UP000001303 | 206 | 0 | 0 | 0 | 0 | 0 | 0 | 0 | 3 |
| 1401065 | *Oligella* | *urethralis* | UP000029629 | 2118 | 0 | 2 | 1 | 1 | 1 | 0 | 0 | 99 |
| 1403537 | *Fervidicella* | *metallireducens* | UP000019681 | 2800 | 0 | 0 | 1 | 1 | 0 | 1 | 1 | 98 |
| 1403945 | *Negativicoccus* | *succinicivorans* | UP000018840 | 1679 | 2 | 1 | 1 | 2 | 1 | 0 | 0 | 58 |
| 1403948 | *Varibaculum* | *cambriense* | UP000018843 | 2003 | 1 | 1 | 0 | 1 | 1 | 4 | 0 | 53 |
| 1408226 | *Vagococcus* | *lutrae* | UP000018126 | 1736 | 0 | 0 | 0 | 0 | 1 | 4 | 0 | 85 |
| 1408281 | *Endomicrobium* | *proavitum* | UP000035337 | 1336 | 0 | 0 | 1 | 0 | 1 | 1 | 1 | 38 |
| 1414851 | *Pelistega* | *indica* | UP000018766 | 2121 | 0 | 2 | 1 | 1 | 1 | 0 | 0 | 94 |
| 1429043 | *Dethiosulfatarculus* | *sandiegensis* | UP000032233 | 5395 | 0 | 0 | 1 | 1 | 1 | 2 | 2 | 251 |
| 1432561 | *Klebsiella* | *pneumoniae* | UP000019190 | 5313 | 1 | 1 | 1 | 2 | 1 | 5 | 0 | 169 |
| 1433126 | *Mucinivorans* | *hirudinis* | UP000027616 | 2816 | 1 | 0 | 1 | 1 | 1 | 3 | 0 | 71 |
| 1437824 | *Castellaniella* | *defragrans* | UP000019805 | 3575 | 1 | 0 | 1 | 1 | 1 | 0 | 0 | 222 |
| 1441930 | *Chania* | *multitudinisentens* | UP000019030 | 4521 | 1 | 0 | 2 | 2 | 1 | 5 | 1 | 336 |
| 1443103 | *Snodgrassella* | *alvi* | UP000028323 | 1571 | 0 | 1 | 1 | 0 | 1 | 0 | 1 | 60 |
| 1444315 | *Lysobacter* | *capsici* | UP000023435 | 5139 | 1 | 1 | 1 | 1 | 0 | 2 | 1 | 204 |
| 1445510 | *Gynuella* | *sunshinyii* | UP000032266 | 5913 | 1 | 0 | 0 | 2 | 1 | 4 | 1 | 339 |
| 1449976 | *Kutzneria* | *albida* | UP000019225 | 8775 | 1 | 1 | 0 | 2 | 1 | 16 | 0 | 290 |
| 1453496 | *Hafnia* | *alvei* | UP000029986 | 4059 | 1 | 0 | 1 | 2 | 1 | 6 | 1 | 271 |
| 1453500 | *Schleiferia* | *thermophila* | UP000028720 | 2301 | 1 | 0 | 1 | 2 | 1 | 0 | 1 | 65 |
| 1454006 | *Siansivirga* | *zeaxanthinifaciens* | UP000032229 | 2744 | 1 | 2 | 1 | 1 | 1 | 0 | 0 | 55 |
| 1461693 | *Actibacterium* | *atlanticum* | UP000024836 | 3126 | 1 | 3 | 1 | 2 | 0 | 2 | 1 | 91 |
| 1461694 | *Pseudooceanicola* | *atlanticus* | UP000030004 | 4202 | 1 | 2 | 1 | 2 | 0 | 1 | 1 | 124 |
| 1472767 | *Lentibacillus* | *amyloliquefaciens* | UP000050331 | 3553 | 0 | 0 | 0 | 0 | 1 | 2 | 2 | 98 |
| 1479485 | *Tolypothrix* | *bouteillei* | UP000029738 | 7755 | 1 | 0 | 2 | 1 | 1 | 2 | 0 | 247 |
| 1486262 | *Martelella* | *endophytica* | UP000032611 | 4020 | 1 | 1 | 1 | 2 | 1 | 5 | 0 | 196 |
| 1497020 | *Neosynechococcus* | *sphagnicola* | UP000030170 | 2723 | 0 | 2 | 1 | 1 | 1 | 0 | 0 | 68 |
| 1514904 | *Ahrensia* | *marina* | UP000038011 | 3161 | 1 | 1 | 1 | 2 | 1 | 3 | 0 | 112 |
| 1524460 | *Phaeodactylibacter* | *xiamenensis* | UP000029736 | 5064 | 2 | 1 | 1 | 1 | 1 | 4 | 1 | 88 |
| 1543381 | *Oleiagrimonas* | *soli* | UP000029708 | 2676 | 1 | 1 | 1 | 1 | 1 | 0 | 0 | 134 |
| 1547597 | *Sanguibacteroides* | *justesenii* | UP000031980 | 2587 | 0 | 0 | 0 | 0 | 1 | 1 | 0 | 94 |
| 1549748 | *Kiloniella* | *litopenaei* | UP000034491 | 3735 | 1 | 0 | 1 | 1 | 1 | 3 | 1 | 142 |
| 1562970 | *Fermentimonas* | *caenicola* | UP000032417 | 2391 | 1 | 1 | 1 | 0 | 1 | 6 | 0 | 60 |
| 1572751 | *Porphyrobacter* | *mercurialis* | UP000030988 | 2758 | 1 | 1 | 1 | 2 | 1 | 2 | 0 | 105 |
| 1574623 | *Lyngbya* | *confervoides* | UP000031561 | 6060 | 2 | 2 | 2 | 1 | 1 | 2 | 1 | 151 |
| 1577792 | *Terrisporobacter* | *othiniensis* | UP000031189 | 3449 | 0 | 0 | 1 | 2 | 1 | 2 | 1 | 120 |
| 1594576 | *Mastigocladus* | *laminosus* | UP000032368 | 5930 | 2 | 1 | 2 | 1 | 1 | 1 | 0 | 197 |
| 1618023 | *Chroococcales* | *cyanobacterium* | UP000032452 | 4366 | 1 | 1 | 2 | 0 | 1 | 2 | 0 | 145 |
| 1629334 | *Caedibacter* | *varicaedens* | UP000036771 | 1663 | 0 | 0 | 1 | 1 | 0 | 0 | 1 | 70 |
| 1637645 | *Limnoraphis* | *robusta* | UP000033607 | 5035 | 0 | 1 | 2 | 2 | 1 | 1 | 0 | 163 |
| 1666911 | *Phormidesmis* | *priestleyi* | UP000050465 | 4851 | 1 | 1 | 2 | 1 | 2 | 1 | 1 | 160 |

^*^ *arp* is a set of actin-related genes and their corresponding protein products form a complex and are suspected to be an unknown mechanism for polyP synthesis in bacteria.
