# Supplementary material for: Distribution Patterns of Polyphosphate Metabolism Pathway and Its Relationships With Bacterial Durability and Virulence
Source: Front Microbiol. 2018 Apr 24;9:782. doi: 10.3389/fmicb.2018.00782 (PMC5932413; doi:10.3389/fmicb.2018.00782)
Supplement: Supplementary file 2 [file Table_2.DOCX]

**Table S2. Lifestyle and environment persistence of bacteria with complete polyP metabolism pathway**

| **Taxonomy ID** | **Genus** | **Species** | **Protein count** | **Virulence** | **Host**  **/Niche** | **Disease** | **Transmission** | **Lifestyle** | **Persistence** | **Ref.** |
| --- | --- | --- | --- | --- | --- | --- | --- | --- | --- | --- |
| 329726 | *Acaryochloris* | *marina* | 8172 | 199 | coral | n/a | n/a | free-living or  symbiotic | infinite | [[1](#_ENREF_1)] |
| 522306 | *Accumulibacter* | *phosphatis* | 4438 | 247 | n/a | n/a | water-borne | free-living | infinite | [[2](#_ENREF_2)] |
| 243159 | *Acidithiobacillus* | *ferrooxidans* | 3120 | 136 | n/a | n/a | water-borne | free-living | infinite | [[3](#_ENREF_3)] |
| 397945 | *Acidovorax* | *citrulli* | 4602 | 283 | plant | fruit blotch | seed-borne | n/a | >30 years | [4] |
| 909613 | *Actinokineospora* | *spheciospongiae* | 6610 | 209 | plant | n/a | sponge-associated | free-living | infinite | [[5](#_ENREF_5)] |
| 512565 | *Actinoplanes* | *missouriensis* | 8113 | 221 | soil | n/a | n/a | free-living | infinite | [[6](#_ENREF_6)] |
| 446462 | *Actinosynnema* | *mirum* | 6912 | 241 | soil | n/a | n/a | free-living | infinite | [[7](#_ENREF_7)] |
| 380703 | *Aeromonas* | *hydrophila* | 4121 | 296 | water | gastroenteritis | food-borne | free-living | infinite | [[8](#_ENREF_8)] |
| 1331007 | *Agarivorans* | *albus* | 4397 | 222 | molluscs | n/a | n/a | n/a | n/a | [[9](#_ENREF_9)] |
| 1330458 | *Agrococcus* | *pavilionensis* | 2776 | 83 | water | n/a | n/a | free-living | infinite | [[10](#_ENREF_10)] |
| 1514904 | *Ahrensia* | *marina* | 3161 | 112 | water | n/a | n/a | free-living | infinite | [[11](#_ENREF_11)] |
| 388413 | *Algoriphagus* | *machipongonensis* | 3932 | 90 | mud | n/a | n/a | free-living | infinite | [[12](#_ENREF_12)] |
| 596154 | *Alicycliphilus* | *denitrificans* | 4620 | 220 | water  soil | n/a | n/a | free-living | infinite | [[13](#_ENREF_13)] |
| 1267766 | *Altererythrobacter* | *atlanticus* | 3254 | 113 | water | n/a | n/a | free-living | infinite | [[14](#_ENREF_14)] |
| 584708 | *Aminomonas* | *paucivorans* | 2391 | 111 | n/a | n/a | n/a | n/a | n/a | n/a |
| 713604 | *Amycolatopsis* | *mediterranei* | 9551 | 210 | n/a | n/a | n/a | n/a | n/a | n/a |
| 272123 | *Anabaena* | *cylindrica* | 5797 | 165 | water | n/a | n/a | free-living | infinite | [[15](#_ENREF_15)] |
| 47500 | *Aneurinibacillus* | *migulanus* | 5649 | 236 | soil | n/a | n/a | free-living | infinite | [[16](#_ENREF_16)] |
| 69279 | *Aquamicrobium* | *defluvii* | 4454 | 173 | sewage | n/a | n/a | free-living | infinite | [[17](#_ENREF_17)] |
| 1317122 | *Aquimarina* | *atlantica* | 4786 | 121 | seawater | n/a | n/a | free-living | infinite | [[18](#_ENREF_18)] |
| 48 | *Archangium* | *gephyra* | 10110 | 335 | soil | n/a | n/a | free-living | infinite | [[19](#_ENREF_19)] |
| 696747 | *Arthrospira* | *platensis* | 6009 | 124 | water | n/a | n/a | free-living | infinite | [[20](#_ENREF_20)] |
| 287752 | *Aurantimonas* | *manganoxydans* | 3625 | 154 | seawater | n/a | n/a | free-living | infinite | [[21](#_ENREF_21)] |
| 370622 | *Aureimonas* | *altamirensis* | 3519 | 154 | subterranean | peritonitis | n/a | free-living | infinite | [[22](#_ENREF_22)] |
| 322710 | *Azotobacter* | *vinelandii* | 4990 | 308 | soil | n/a | n/a | free-living | infinite | [[23](#_ENREF_23)] |
| 226186 | *Bacteroides* | *thetaiotaomicron* | 4782 | 119 | human gastrointestinal tract | opportunistic pathogen | n/a | commensal | n/a | [[24](#_ENREF_24)] |
| 395963 | *Beijerinckia* | *indica* | 3774 | 153 | soil | n/a | n/a | free-living | infinite | [[25](#_ENREF_25)] |
| 471853 | *Beutenbergia* | *cavernae* | 4195 | 104 | cave | n/a | n/a | free-living | infinite | [[26](#_ENREF_26)] |
| 314230 | *Blastopirellula* | *marina* | 6010 | 149 | seawater | n/a | n/a | free-living | infinite | [[27](#_ENREF_27)] |
| 359391 | *Brucella* | *abortus* | 3022 | 152 | human | brucellosis | blood-borne | parasite | n/a | [[28](#_ENREF_28)] |
| 272560 | *Burkholderia* | *pseudomallei* | 5717 | 510 | soil | Melioidosis  (fatal) | sit-and-wait | free-living | infinite | [[29](#_ENREF_29)] |
| 926550 | *Caldilinea* | *aerophila* | 4097 | 121 | hot spring | n/a | n/a | free-living  (thermophile) | infinite | [[30](#_ENREF_30)] |
| 768670 | *Calditerrivibrio* | *nitroreducens* | 2089 | 120 | hot spring | n/a | n/a | free-living  (thermophile) | infinite | [[31](#_ENREF_31)] |
| 1225176 | *Cecembia* | *lonarensis* | 4213 | 104 | water | n/a | n/a | free-living | infinite | [[32](#_ENREF_32)] |
| 867900 | *Cellulophaga* | *lytica* | 3281 | 71 | mud | n/a | n/a | free-living | infinite | [[33](#_ENREF_33)] |
| 264251 | *Cellulosimicrobium* | *funkei* | 3980 | 99 | hot spring | bacteraemia | n/a | free-living | infinite | [[34](#_ENREF_34)] |
| 1173020 | *Chamaesiphon* | *minutus* | 5815 | 168 | n/a | n/a | n/a | n/a | n/a | n/a |
| 505317 | *Chelonobacter* | *oris* | 2284 | 121 | tortoise | respiratory tract infection | n/a | n/a | n/a | [[35](#_ENREF_35)] |
| 485918 | *Chitinophaga* | *pinensis* | 7179 | 181 | soil | n/a | n/a | n/a | n/a | [[36](#_ENREF_36)] |
| 194439 | *Chlorobium* | *tepidum* | 2250 | 62 | water | n/a | n/a | free-living  (thermophile) | infinite | [[37](#_ENREF_37)] |
| 324602 | *Chloroflexus* | *aurantiacus* | 3850 | 133 | n/a | n/a | n/a | free-living  (photosynthetic) | infinite | [[38](#_ENREF_38)] |
| 517418 | *Chloroherpeton* | *thalassium* | 2708 | 82 | water | n/a | n/a | free-living  (autotroph) | infinite | [[39](#_ENREF_39)] |
| 243365 | *Chromobacterium* | *violaceum* | 4397 | 341 | water/soil | skin infection | n/a | free-living | infinite | [[40](#_ENREF_40)] |
| 251229 | *Chroococcidiopsis* | *thermalis* | 5740 | 268 | desert | n/a | n/a | free-living  (extremophile) | infinite | [[41](#_ENREF_41)] |
| 510955 | *Chryseobacterium* | *solincola* | 2087 | 60 | soil | n/a | n/a | free-living | infinite | [[42](#_ENREF_42)] |
| 1303518 | *Chthonomonas* | *calidirosea* | 2809 | 109 | hot soil | n/a | n/a | free-living | infinite | [[43](#_ENREF_43)] |
| 118168 | *Coleofasciculus* | *chthonoplastes* | 8193 | 162 | marine | n/a | n/a | free-living | infinite | [[44](#_ENREF_44)] |
| 469383 | *Conexibacter* | *woesei* | 5912 | 166 | forest soil | n/a | n/a | free-living | infinite | [[45](#_ENREF_45)] |
| 583355 | *Coraliomargarita* | *akajimensis* | 3110 | 79 | seawater | n/a | n/a | free-living | infinite | [[46](#_ENREF_46)] |
| 1144275 | *Corallococcus* | *coralloides* | 8019 | 315 | n/a | n/a | n/a | n/a | n/a | n/a |
| 1173022 | *Crinalium* | *epipsammum* | 4697 | 133 | sand dunes | n/a | n/a | free-living | infinite | [[47](#_ENREF_47)] |
| 1300341 | *Croceitalea* | *dokdonensis* | 3682 | 58 | marine | n/a | n/a | n/a | n/a | [[48](#_ENREF_48)] |
| 165597 | *Crocosphaera* | *watsonii* | 5659 | 130 | marine | n/a | n/a | free-living | infinite | [[49](#_ENREF_49)] |
| 755178 | *Cyanobacterium* | *aponinum* | 3415 | 128 | water | n/a | n/a | free-living | infinite | [[50](#_ENREF_50)] |
| 292563 | *Cyanobacterium* | *stanieri* | 2831 | 94 | water | n/a | n/a | free-living | infinite | [[51](#_ENREF_51)] |
| 292564 | *Cyanobium* | *gracile* | 3261 | 94 | water | n/a | n/a | free-living | infinite | [[52](#_ENREF_52)] |
| 880070 | *Cyclobacterium* | *marinum* | 4983 | 102 | sea sediment | n/a | n/a | n/a | n/a | [[53](#_ENREF_53)] |
| 56107 | *Cylindrospermum* | *stagnale* | 6200 | 190 | soil | n/a | n/a | free-living | infinite | [[54](#_ENREF_54)] |
| 1242864 | *Cystobacter* | *fuscus* | 10511 | 357 | n/a | n/a | n/a | n/a | n/a | n/a |
| 269798 | *Cytophaga* | *hutchinsonii* | 3771 | 79 | soil | n/a | n/a | free-living | infinite | [[55](#_ENREF_55)] |
| 159087 | *Dechloromonas* | *aromatica* | 4155 | 250 | soil | n/a | n/a | free-living | infinite | [[56](#_ENREF_56)] |
| 640081 | *Dechlorosoma* | *suillum* | 3432 | 247 | animal waste | n/a | n/a | n/a | n/a | [[57](#_ENREF_57)] |
| 522772 | *Denitrovibrio* | *acetiphilus* | 2901 | 137 | oil reservoir | n/a | n/a | n/a | n/a | [[58](#_ENREF_58)] |
| 694431 | *Desulfurella* | *acetivorans* | 1825 | 105 | sediment | n/a | n/a | free-living | infinite | [[59](#_ENREF_59)] |
| 653733 | *Desulfurispirillum* | *indicum* | 2551 | 151 | river sediment | n/a | n/a | free-living | infinite | [[60](#_ENREF_60)] |
| 469381 | *Dethiosulfovibrio* | *peptidovorans* | 2432 | 104 | oil well | n/a | n/a | free-living | infinite | [[61](#_ENREF_61)] |
| 46914 | *Devosia* | *riboflavina* | 4273 | 176 | soil | n/a | n/a | free-living | infinite | [[62](#_ENREF_62)] |
| 742743 | *Dialister* | *succinatiphilus* | 2140 | 68 | human feces | n/a | n/a | n/a | n/a | [[63](#_ENREF_63)] |
| 1168034 | *Draconibacterium* | *orientale* | 3940 | 95 | marine | n/a | n/a | n/a | n/a | [[64](#_ENREF_64)] |
| 471854 | *Dyadobacter* | *fermentans* | 5703 | 142 | plant | n/a | n/a | n/a | n/a | [[65](#_ENREF_65)] |
| 742766 | *Dysgonomonas* | *gadei* | 4153 | 87 | gall bladder | pathogenic | n/a | n/a | n/a | [[66](#_ENREF_66)] |
| 926556 | *Echinicola* | *vietnamensis* | 4509 | 97 | marine | n/a | n/a | n/a | n/a | [[67](#_ENREF_67)] |
| 1338011 | *Elizabethkingia* | *anophelis* | 4061 | 103 | mosquito | pathogenic | mosquito-borne | n/a | n/a | [[68](#_ENREF_68)] |
| 929562 | *Emticicia* | *oligotrophica* | 4251 | 106 | warm spring water | n/a | n/a | n/a | n/a | [[69](#_ENREF_69)] |
| 701347 | *Enterobacter* | *lignolyticus* | 4393 | 312 | soil | n/a | n/a | free-living | infinite | [[70](#_ENREF_70)] |
| 1044 | *Erythrobacter* | *longus* | 3219 | 109 | seaweed | n/a | n/a | n/a | n/a | [[71](#_ENREF_71)] |
| 550540 | *Ferrimonas* | *balearica* | 3781 | 203 | marine sediment | n/a | n/a | free-living | infinite | [[72](#_ENREF_72)] |
| 1166018 | *Fibrella* | *aestuarina* | 5627 | 125 | sea mud | n/a | n/a | n/a | n/a | [[73](#_ENREF_73)] |
| 1185876 | *Fibrisoma* | *limi* | 6341 | 132 | sea mud | n/a | n/a | n/a | n/a | [[74](#_ENREF_74)] |
| 661478 | *Fimbriimonas* | *ginsengisoli* | 4817 | 96 | soil | n/a | n/a | free-living | infinite | [[75](#_ENREF_75)] |
| 1349421 | *Flavihumibacter* | *solisilvae* | 4215 | 109 | soil | n/a | n/a | free-living | infinite | [[76](#_ENREF_76)] |
| 1086011 | *Flavobacterium* | *frigoris* | 3590 | 85 | Antarctic lake | n/a | n/a | free-living | infinite | [[77](#_ENREF_77)] |
| 326424 | *Frankia* | *alni* | 6710 | 178 | soil | n/a | n/a | free-living | infinite | [[78](#_ENREF_78)] |
| 1237149 | *Fulvivirga* | *imtechensis* | 5952 | 142 | seawater | n/a | n/a | free-living | infinite | [[79](#_ENREF_79)] |
| 555500 | *Galbibacter* | *marinus* | 3099 | 71 | deep sea sediment | n/a | n/a | free-living | infinite | [[80](#_ENREF_80)] |
| 745411 | *Gallaecimonas* | *xiamenensis* | 3798 | 231 | sea water | n/a | n/a | free-living | infinite | [[81](#_ENREF_81)] |
| 379066 | *Gemmatimonas* | *aurantiaca* | 3932 | 167 | waste water | n/a | n/a | free-living | infinite | [[82](#_ENREF_82)] |
| 861299 | *Gemmatirosa* | *kalamazoonesis* | 6261 | 226 | soil | n/a | n/a | free-living | infinite | [[83](#_ENREF_83)] |
| 243231 | *Geobacter* | *sulfurreducens* | 3402 | 202 | fresh water | n/a | n/a | free-living | infinite | [[84](#_ENREF_84)] |
| 865937 | *Gillisia* | *limnaea* | 3411 | 90 | lake | n/a | n/a | free-living | infinite | [[85](#_ENREF_85)] |
| 344747 | *Gimesia* | *maris* | 6463 | 163 | marine | n/a | n/a | n/a | n/a | n/a |
| 251221 | *Gloeobacter* | *violaceus* | 4406 | 141 | water | n/a | n/a | free-living | infinite | [[86](#_ENREF_86)] |
| 272568 | *Gluconacetobacter* | *diazotrophicus* | 3783 | 134 | sugarcane | n/a | n/a | symbiotic | n/a | [[87](#_ENREF_87)] |
| 411154 | *Gramella* | *forsetii* | 3554 | 78 | water | n/a | n/a | free-living | infinite | [[88](#_ENREF_88)] |
| 502025 | *Haliangium* | *ochraceum* | 6684 | 251 | ocean | n/a | n/a | free-living | infinite | [[89](#_ENREF_89)] |
| 760192 | *Haliscomenobacter* | *hydrossis* | 6704 | 133 | waste water | n/a | n/a | free-living | infinite | [[90](#_ENREF_90)] |
| 555778 | *Halothiobacillus* | *neapolitanus* | 2353 | 157 | n/a | n/a | n/a | free-living | infinite | [[91](#_ENREF_91)] |
| 1304833 | *Hassallia* | *byssoidea* | 10137 | 256 | monument | n/a | n/a | free-living | infinite | [[92](#_ENREF_92)] |
| 316274 | *Herpetosiphon* | *aurantiacus* | 5254 | 204 | water/hot spring | n/a | n/a | free-living | infinite | [[93](#_ENREF_93)] |
| 582402 | *Hirschia* | *baltica* | 3187 | 108 | marine | n/a | n/a | free-living | infinite | [[94](#_ENREF_94)] |
| 394096 | *Hyalangium* | *minutum* | 8967 | 323 | terrestrial | n/a | n/a | free-living | infinite | [[95](#_ENREF_95)] |
| 28885 | *Hydrogenovibrio* | *marinus* | 2330 | 147 | marine | n/a | n/a | free-living | infinite | [[96](#_ENREF_96)] |
| 945713 | *Ignavibacterium* | *album* | 3169 | 116 | hot spring | n/a | n/a | free-living | infinite | [[97](#_ENREF_97)] |
| 572544 | *Ilyobacter* | *polytropus* | 2859 | 99 | marine sediment | n/a | n/a | free-living | infinite | [[98](#_ENREF_98)] |
| 743718 | *Isoptericola* | *variabilis* | 2879 | 76 | termite | n/a | n/a | n/a | n/a | [[99](#_ENREF_99)] |
| 1349767 | *Janthinobacterium* | *agaricidamnosum* | 5488 | 362 | water/soil/mushroom | soft-rot disease | n/a | n/a | n/a | [[100](#_ENREF_100)] |
| 1432561 | *Klebsiella* | *pneumoniae* | 5313 | 169 | human | pneumonia | water/soil | free-living | infinite | [[101](#_ENREF_101)] |
| 479435 | *Kribbella* | *flavida* | 6940 | 156 | soil | n/a | n/a | free-living | infinite | [[102](#_ENREF_102)] |
| 244592 | *Labrenzia* | *alexandrii* | 5363 | 191 | dinoflagellate | n/a | n/a | n/a | n/a | [[103](#_ENREF_103)] |
| 557598 | *Laribacter* | *hongkongensis* | 3208 | 192 | human/water | emerging pathogen | n/a | free-living | infinite | [[104](#_ENREF_104)] |
| 398720 | *Leeuwenhoekiella* | *blandensis* | 3687 | 70 | seawater | n/a | n/a | free-living | infinite | [[105](#_ENREF_105)] |
| 395495 | *Leptothrix* | *cholodnii* | 4343 | 259 | aquatic | n/a | n/a | free-living | infinite | [[106](#_ENREF_106)] |
| 1123360 | *Litoreibacter* | *arenae* | 3640 | 101 | sea sand | n/a | n/a | free-living | infinite | [[107](#_ENREF_107)] |
| 1574623 | *Lyngbya* | *confervoides* | 6060 | 151 | marine | n/a | n/a | free-living | infinite | [[108](#_ENREF_108)] |
| 1239962 | *Mariniradius* | *saccharolyticus* | 4633 | 84 | marine | n/a | n/a | free-living | infinite | [[109](#_ENREF_109)] |
| 351348 | *Marinobacter* | *hydrocarbonoclasticus* | 4170 | 244 | seawater | n/a | n/a | free-living | infinite | [[110](#_ENREF_110)] |
| 717774 | *Marinomonas* | *mediterranea* | 4118 | 268 | sea | n/a | n/a | free-living | infinite | [[111](#_ENREF_111)] |
| 643867 | *Marivirga* | *tractuosa* | 3748 | 80 | beach sand | n/a | n/a | free-living | infinite | [[112](#_ENREF_112)] |
| 1486262 | *Martelella* | *endophytica* | 4020 | 196 | plant root | n/a | n/a | n/a | n/a | [[113](#_ENREF_113)] |
| 883126 | *Massilia* | *timonae* | 5096 | 309 | human | possibly pathogenic | n/a | n/a | n/a | [[114](#_ENREF_114)] |
| 1594576 | *Mastigocladus* | *laminosus* | 5930 | 197 | hot spring | n/a | n/a | free-living | infinite | [[115](#_ENREF_115)] |
| 420662 | *Methylibium* | *petroleiphilum* | 4359 | 208 | oil | non-pathogenic | n/a | free-living | infinite | [[116](#_ENREF_116)] |
| 265072 | *Methylobacillus* | *flagellatus* | 2609 | 198 | fresh water | n/a | n/a | free-living | infinite | [[117](#_ENREF_117)] |
| 426355 | *Methylobacterium* | *radiotolerans* | 6388 | 260 | plant root | n/a | n/a | n/a | n/a | [[118](#_ENREF_118)] |
| 395965 | *Methylocella* | *silvestris* | 3816 | 161 | soil | n/a | n/a | free-living | infinite | [[119](#_ENREF_119)] |
| 243233 | *Methylococcus* | *capsulatus* | 2925 | 156 | sewer sludge | n/a | n/a | free-living | infinite | [[120](#_ENREF_120)] |
| 857087 | *Methylomonas* | *methanica* | 4435 | 273 | seawater | n/a | n/a | free-living | infinite | [[121](#_ENREF_121)] |
| 666681 | *Methylotenera* | *versatilis* | 2752 | 198 | lake sediment | n/a | n/a | free-living | infinite | [[122](#_ENREF_122)] |
| 1000565 | *Methyloversatilis* | *universalis* | 3919 | 243 | lake sediment | n/a | n/a | free-living | infinite | [[123](#_ENREF_123)] |
| 582744 | *Methylovorus* | *glucosetrophus* | 2907 | 226 | lake sediment | n/a | n/a | free-living | infinite | [[124](#_ENREF_124)] |
| 449447 | *Microcystis* | *aeruginosa* | 5981 | 118 | fresh water | n/a | n/a | free-living | infinite | [[125](#_ENREF_125)] |
| 644283 | *Micromonospora* | *aurantiaca* | 6204 | 177 | soil | n/a | n/a | free-living | infinite | [[126](#_ENREF_126)] |
| 313606 | *Microscilla* | *marina* | 8248 | 123 | marine | n/a | n/a | free-living | infinite | [[127](#_ENREF_127)] |
| 864069 | *Microvirga* | *lotononidis* | 6918 | 237 | root | n/a | n/a | free-living or symbiotic | infinite | [[128](#_ENREF_128)] |
| 489825 | *Moorea* | *producens* | 7382 | 198 | marine | n/a | n/a | free-living | infinite | [[129](#_ENREF_129)] |
| 714943 | *Mucilaginibacter* | *paludis* | 6864 | 131 | acidic peat bog | n/a | n/a | free-living | infinite | [[130](#_ENREF_130)] |
| 1348852 | *Mumia* | *flava* | 14150 | 676 | soil | n/a | n/a | free-living | infinite | [[131](#_ENREF_131)] |
| 246197 | *Myxococcus* | *xanthus* | 7314 | 299 | soil | n/a | n/a | free-living | infinite | [[132](#_ENREF_132)] |
| 207954 | *Neptuniibacter* | *caesariensis* | 3687 | 214 | surface water | n/a | n/a | free-living | infinite | [[133](#_ENREF_133)] |
| 929713 | *Niabella* | *soli* | 4110 | 99 | soil | n/a | n/a | free-living | infinite | [[134](#_ENREF_134)] |
| 700598 | *Niastella* | *koreensis* | 7171 | 156 | soil | n/a | n/a | free-living | infinite | [[135](#_ENREF_135)] |
| 749222 | *Nitratifractor* | *salsuginis* | 2076 | 87 | deep-sea | n/a | n/a | free-living | infinite | [[136](#_ENREF_136)] |
| 313624 | *Nodularia* | *spumigena* | 5211 | 157 | water | n/a | n/a | free-living | infinite | [[137](#_ENREF_137)] |
| 63737 | *Nostoc* | *punctiforme* | 6573 | 258 | water | n/a | n/a | free-living | infinite | [[138](#_ENREF_138)] |
| 1207063 | *Oceanibaculum* | *indicum* | 3754 | 156 | deep sea | n/a | n/a | free-living | infinite | [[139](#_ENREF_139)] |
| 391624 | *Oceanibulbus* | *indolifex* | 4147 | 118 | marine | n/a | n/a | free-living | infinite | [[140](#_ENREF_140)] |
| 439375 | *Ochrobactrum* | *anthropi* | 4757 | 209 | plant nodule | human pathogen | n/a | symbiotic | n/a | [[141](#_ENREF_141)] |
| 452637 | *Opitutus* | *terrae* | 4588 | 162 | rice | n/a | n/a | free-living | infinite | [[142](#_ENREF_142)] |
| 867902 | *Ornithobacterium* | *rhinotracheale* | 2138 | 54 | turkey  chicken | respiratory infection | n/a | n/a | n/a | [[143](#_ENREF_143)] |
| 56110 | *Oscillatoria* | *acuminata* | 5755 | 171 | n/a | n/a | n/a | n/a | n/a | n/a |
| 864702 | *Oscillatoriales* | *cyanobacterium* | 4673 | 138 | n/a | n/a | n/a | free-living | infinite | [[144](#_ENREF_144)] |
| 706191 | *Pantoea* | *ananatis* | 4241 | 231 | plant  human | pathogenic | n/a | n/a | n/a | [[145](#_ENREF_145)] |
| 402881 | *Parvibaculum* | *lavamentivorans* | 3580 | 125 | n/a | n/a | n/a | free-living | infinite | [[146](#_ENREF_146)] |
| 1097667 | *Patulibacter* | *medicamentivorans* | 4372 | 113 | wastewater sludge | n/a | n/a | free-living | infinite | [[147](#_ENREF_147)] |
| 485917 | *Pedobacter* | *heparinus* | 4249 | 115 | soil/water | n/a | n/a | free-living | infinite | [[148](#_ENREF_148)] |
| 1082931 | *Pelagibacterium* | *halotolerans* | 3875 | 153 | seawater | n/a | n/a | free-living | infinite | [[149](#_ENREF_149)] |
| 324925 | *Pelodictyon* | *phaeoclathratiforme* | 2685 | 94 | lake water | n/a | n/a | free-living | infinite | [[150](#_ENREF_150)] |
| 1524460 | *Phaeodactylibacter* | *xiamenensis* | 5064 | 88 | marine algae | n/a | n/a | n/a | n/a | [[151](#_ENREF_151)] |
| 450851 | *Phenylobacterium* | *zucineum* | 3838 | 146 | human | potentially pathogenic | n/a | facultative intracellular | n/a | [[152](#_ENREF_152)] |
| 1666911 | *Phormidesmis* | *priestleyi* | 4851 | 160 | Arctic | n/a | n/a | free-living | infinite | [[153](#_ENREF_153)] |
| 74109 | *Photobacterium* | *profundum* | 5328 | 270 | deep sea | n/a | n/a | free-living | infinite | [[154](#_ENREF_154)] |
| 530564 | *Pirellula* | *staleyi* | 4711 | 137 | water | n/a | n/a | free-living | infinite | [[155](#_ENREF_155)] |
| 521674 | *Planctopirus* | *limnophila* | 4258 | 130 | lake water | n/a | n/a | free-living | infinite | [[156](#_ENREF_156)] |
| 388467 | *Planktothrix* | *agardhii* | 4186 | 118 | lake water | n/a | n/a | free-living | infinite | [[157](#_ENREF_157)] |
| 1315976 | *Plesiomonas* | *shigelloides* | 3377 | 222 | water human | diarrheal | n/a | free-living | infinite | [[158](#_ENREF_158)] |
| 365044 | *Polaromonas* | *naphthalenivorans* | 4879 | 171 | freshwater sediment | n/a | n/a | free-living | infinite | [[159](#_ENREF_159)] |
| 413882 | *Polyangium* | *brachysporum* | 5541 | 281 | n/a | n/a | n/a | n/a | n/an | n/a |
| 991905 | *Polymorphum* | *gilvum* | 4354 | 160 | saline soil | n/a | n/a | free-living | infinite | [[160](#_ENREF_160)] |
| 1572751 | *Porphyrobacter* | *mercurialis* | 2758 | 105 | stadium seat | n/a | n/a | n/a | n/a | [[161](#_ENREF_161)] |
| 290512 | *Prosthecochloris* | *aestuarii* | 2319 | 72 | n/a | n/a | n/a | free-living | infinite | [[162](#_ENREF_162)] |
| 665126 | *Prosthecomicrobium* | *hirschii* | 5501 | 197 | freshwater pond | n/a | n/a | free-living | infinite | [[163](#_ENREF_163)] |
| 338969 | *Rhodoferax* | *ferrireducens* | 4401 | 262 | subsurface sediment | n/a | n/a | free-living | infinite | [[164](#_ENREF_164)] |
| 1123057 | *Rhodonellum* | *psychrophilum* | 4904 | 100 | marine  freshwater | n/a | n/a | free-living | infinite | [[165](#_ENREF_165)] |
| 243090 | *Rhodopirellula* | *baltica* | 7271 | 117 | marine  freshwater | n/a | n/a | free-living | infinite | [[166](#_ENREF_166)] |
| 1228997 | *Riemerella* | *anatipestifer* | 2187 | 54 | poultry | septicaemia | n/a | n/a | n/a | [[167](#_ENREF_167)] |
| 582515 | *Rubidibacter* | *lacunae* | 3455 | 118 | sea water | n/a | n/a | free-living | infinite | [[168](#_ENREF_168)] |
| 756272 | *Rubinisphaera* | *brasiliensis* | 4710 | 144 | water | n/a | n/a | free-living | infinite | [[156](#_ENREF_156)] |
| 761193 | *Runella* | *slithyformis* | 5750 | 124 | water | n/a | n/a | n/a | n/a | [[169](#_ENREF_169)] |
| 869213 | *Saccharicrinis* | *fermentans* | 4663 | 107 | marine sediment | n/a | n/a | free-living | infinite | [[170](#_ENREF_170)] |
| 1179773 | *Saccharothrix* | *espanaensis* | 8423 | 234 | soil | n/a | n/a | free-living | infinite | [[171](#_ENREF_171)] |
| 1233231 | *Scytonema* | *tolypothrichoides* | 7078 | 186 | n/a | n/a | n/a | free-living | infinite | [[172](#_ENREF_172)] |
| 526218 | *Sebaldella* | *termitidis* | 4124 | 92 | termite | n/a | n/a | n/a | n/a | [[173](#_ENREF_173)] |
| 399741 | *Serratia* | *proteamaculans* | 4930 | 335 | water  soil  human | n/a | n/a | free-living | infinite | [[174](#_ENREF_174)] |
| 580332 | *Sideroxydans* | *lithotrophicus* | 2977 | 197 | ground-water | n/a | n/a | free-living | infinite | [[175](#_ENREF_175)] |
| 929556 | *Solitalea* | *canadensis* | 4304 | 99 | soil | n/a | n/a | free-living | infinite | [[176](#_ENREF_176)] |
| 1286631 | *Sphaerotilus* | *natans* | 4177 | 261 | water | n/a | n/a | free-living | infinite | [[177](#_ENREF_177)] |
| 525373 | *Sphingobacterium* | *spiritivorum* | 4471 | 98 | environment | opportunistic pathogen | n/a | free-living | infinite | [[178](#_ENREF_178)] |
| 504472 | *Spirosoma* | *linguale* | 6867 | 148 | water bath | n/n | n/a | free-living | infinite | [[179](#_ENREF_179)] |
| 153721 | *Sporocytophaga* | *myxococcoides* | 5042 | 136 | soil | n/a | n/a | free-living | infinite | [[180](#_ENREF_180)] |
| 111780 | *Stanieria* | *cyanosphaera* | 4751 | 181 | pool | n/a | n/a | free-living | infinite | [[181](#_ENREF_181)] |
| 378806 | *Stigmatella* | *aurantiaca* | 8307 | 314 | soil | n/a | n/a | free-living | infinite | [[182](#_ENREF_182)] |
| 1163617 | *Sulfuricella* | *denitrificans* | 3071 | 221 | fresh water | n/a | n/a | free-living  (autotrophy) | infinite | [[183](#_ENREF_183)] |
| 709032 | *Sulfuricurvum* | *kujiense* | 2794 | 178 | crude-oil | n/a | n/a | free-living  (autotrophy) | infinite | [[184](#_ENREF_184)] |
| 1223802 | *Sulfuritalea* | *hydrogenivorans* | 3578 | 228 | freshwater | n/a | water | free-living  (autotrophy) | infinite | [[185](#_ENREF_185)] |
| 1140 | *Synechococcus* | *elongatus* | 2657 | 89 | water | n/a | water | free-living | infinite | [[186](#_ENREF_186)] |
| 645991 | *Syntrophobotulus* | *glycolicus* | 3105 | 142 | sludge | n/a | n/a | free-living | n/a | [[187](#_ENREF_187)] |
| 1231623 | *Tanticharoenia* | *sakaeratensis* | 3154 | 135 | soil | n/a | n/a | free-living | n/a | [[188](#_ENREF_188)] |
| 1333998 | *Tepidicaulis* | *marinus* | 3398 | 123 | marine sediment | n/a | seawater | free-living | infinite | [[189](#_ENREF_189)] |
| 1298593 | *Thalassolituus* | *oleivorans* | 3634 | 189 | seawater | n/a | seawater | free-living | infinite | [[190](#_ENREF_190)] |
| 525903 | *Thermanaerovibrio* | *acidaminovorans* | 1737 | 78 | refinery | n/a | n/a | n/a | n/a | [[191](#_ENREF_191)] |
| 197221 | *Thermosynechococcus* | *elongatus* | 2451 | 84 | n/a | n/a | n/a | free-living | infinite | [[192](#_ENREF_192)] |
| 717772 | *Thioalkalimicrobium* | *aerophilum* | 2067 | 137 | soda lake | n/a | n/a | free-living  (autotrophy) | infinite | [[193](#_ENREF_193)] |
| 396588 | *Thioalkalivibrio* | *sulfidiphilus* | 3272 | 209 | soda lake | n/a | n/a | free-living  (autotrophy) | infinite | [[194](#_ENREF_194)] |
| 292415 | *Thiobacillus* | *denitrificans* | 2826 | 183 | n/a | n/a | n/a | free-living  (autotrophy) | infinite | [[195](#_ENREF_195)] |
| 317025 | *Thiomicrospira* | *crunogena* | 2187 | 149 | sea | n/a | n/a | free-living  (autotrophy) | infinite | [[196](#_ENREF_196)] |
| 75379 | *Thiomonas* | *intermedia* | 3134 | 172 | sewage | n/a | n/a | free-living | infinite | [[197](#_ENREF_197)] |
| 765913 | *Thiorhodococcus* | *drewsii* | 4731 | 314 | marine | n/a | marine | free-living | infinite | [[198](#_ENREF_198)] |
| 870187 | *Thiothrix* | *nivea* | 4218 | 179 | sludge | n/a | n/a | free-living | infinite | [[199](#_ENREF_199)] |
| 34073 | *Variovorax* | *paradoxus* | 6788 | 318 | n/a | n/a | n/a | free-living | infinite | [[200](#_ENREF_200)] |
| 391735 | *Verminephrobacter* | *eiseniae* | 4911 | 222 | nephridium | n/a | n/a | symbiotic | n/a | [[201](#_ENREF_201)] |
| 78245 | *Xanthobacter* | *autotrophicus* | 4971 | 204 | sludge | n/a | n/a | free-living | infinite | [[202](#_ENREF_202)] |
| 446471 | *Xylanimonas* | *cellulosilytica* | 3441 | 93 | decayed tree | n/a | n/a | free-living | infinite | [[203](#_ENREF_203)] |
| 63186 | *Zobellia* | *galactanivorans* | 4708 | 85 | seaweed | n/a | seawater | symbiotic | n/a | [[204](#_ENREF_204)] |
| 264203 | *Zymomonas* | *mobilis* | 1779 | 104 | n/a | n/a | n/a | free-living | infinite | [[205](#_ENREF_205)] |

**References**

1. Swingley WD, Chen M, Cheung PC, Conrad AL, Dejesa LC, Hao J *et al*. Niche adaptation and genome expansion in the chlorophyll d-producing cyanobacterium Acaryochloris marina*.* *Proc Natl Acad Sci U S A* 105(6), 2005-2010 (2008).

2. Mao Y, Yu K, Xia Y, Chao Y, Zhang T. Genome reconstruction and gene expression of "Candidatus Accumulibacter phosphatis" Clade IB performing biological phosphorus removal*.* *Environ Sci Technol* 48(17), 10363-10371 (2014).

3. Valdes J, Pedroso I, Quatrini R, Dodson RJ, Tettelin H, Blake R, 2nd *et al*. Acidithiobacillus ferrooxidans metabolism: from genome sequence to industrial applications*.* *BMC Genomics* 9 597 (2008).

4. Popović T, Ivanović Ž. Occurrence of Acidovorax citrulli Causing Bacterial Fruit Blotch of Watermelon in Serbia*.* *Plant Disease* 99(6), (2015).

5. Yamamura H, Ohnishi Y, Ishikawa J, Ichikawa N, Ikeda H, Sekine M *et al*. Complete genome sequence of the motile actinomycete Actinoplanes missouriensis 431(T) (= NBRC 102363(T))*.* *Stand Genomic Sci* 7(2), 294-303 (2012).

6. Jang MS, Mouri Y, Uchida K, Aizawa S, Hayakawa M, Fujita N *et al*. Genetic and Transcriptional Analyses of the Flagellar Gene Cluster in Actinoplanes missouriensis*.* *J Bacteriol* 198(16), 2219-2227 (2016).

7. Asamizu S, Abugreen M, Mahmud T. Comparative metabolomic analysis of an alternative biosynthetic pathway to pseudosugars in Actinosynnema mirum DSM 43827*.* *Chembiochem* 14(13), 1548-1551 (2013).

8. R. H, C. B. Modern Trends in Aeromonas hydrophila Disease Management with Fish*.* *Rev Fish Sci* 13(4), 40 (2005).

9. Yasuike M, Nakamura Y, Kai W, Fujiwara A, Fukui Y, Satomi M *et al*. Draft Genome Sequence of Agarivorans albus Strain MKT 106T, an Agarolytic Marine Bacterium*.* *Genome Announc* 1(4), (2013).

10. White RA, 3rd, Grassa CJ, Suttle CA. First draft genome sequence from a member of the genus agrococcus, isolated from modern microbialites*.* *Genome Announc* 1(4), (2013).

11. Liu J, Wang Y, Liu Y, Zhang XH. Ahrensia marina sp. nov., a novel dimethylsulfoniopropionate-cleavage bacterium isolated from seawater and emended descriptions of the genus Ahrensia and Ahrensia kielensis*.* *Int J Syst Evol Microbiol* doi:10.1099/ijsem.0.000805 (2015).

12. Alegado RA, Grabenstatter JD, Zuzow R, Morris A, Huang SY, Summons RE *et al*. Algoriphagus machipongonensis sp. nov., co-isolated with a colonial choanoflagellate*.* *Int J Syst Evol Microbiol* 63(Pt 1), 163-168 (2013).

13. B. HS, Gam ZBA, Rezgui R, Ghram A, Marroufi A, Labat M. Diversity of culturable aerobic bacteria colonizing four petroleum by-products storage reservoirs*.* *Afr J Microbiol Res* 7(21), 6 (2013).

14. Wu YH, Xu L, Meng FX, Zhang DS, Wang CS, Oren A *et al*. Altererythrobacter atlanticus sp. nov., isolated from deep-sea sediment*.* *Int J Syst Evol Microbiol* 64(Pt 1), 116-121 (2014).

15. Smith RV, Evans MC. Nitrogenase activity in cell-free extracts of the blue-green alga, Anabaena cylindrica*.* *J Bacteriol* 105(3), 913-917 (1971).

16. Berditsch M, Afonin S, Ulrich AS. The ability of Aneurinibacillus migulanus (Bacillus brevis) to produce the antibiotic gramicidin S is correlated with phenotype variation*.* *Appl Environ Microbiol* 73(20), 6620-6628 (2007).

17. Bambauer A, Rainey FA, Stackebrandt E, Winter J. Characterization of Aquamicrobium defluvii gen. nov. sp. nov., a thiophene-2-carboxylate-metabolizing bacterium from activated sludge*.* *Arch Microbiol* 169(4), 293-302 (1998).

18. Li G, Lai Q, Sun F, Liu X, Xie Y, Du Y *et al*. Aquimarina atlantica sp. nov., isolated from surface seawater of the Atlantic Ocean*.* *Antonie Van Leeuwenhoek* 106(2), 293-300 (2014).

19. Kunze B, Reichenbach H, Muller R, Hofle G. Aurafuron A and B, new bioactive polyketides from Stigmatella aurantiaca and Archangium gephyra (Myxobacteria). Fermentation, isolation, physico-chemical properties, structure and biological activity*.* *J Antibiot (Tokyo)* 58(4), 244-251 (2005).

20. Ibrahem MD, Ibrahim MA. The potential effects of Spirulina platensis (Arthrospira platensis) on tissue protection of Nile tilapia (Oreochromis niloticus) through estimation of P53 level*.* *J Adv Res* 5(1), 133-136 (2014).

21. Anderson CR, Dick GJ, Chu ML, Cho JC, Davis RE, Brauer SL *et al*. Aurantimonas manganoxydans, sp. nov. and Aurantimonas litoralis, sp. nov.: Mn(II) oxidizing representatives of a globally distributed clade of alpha-Proteobacteria from the order Rhizobiales*.* *Geomicrobiol J* 26(3), 189-198 (2009).

22. Schrottner P, Rudolph WW, Taube F, Gunzer F. First report on the isolation of Aureimonas altamirensis from a patient with peritonitis*.* *Int J Infect Dis* 29 71-73 (2014).

23. Setubal JC, Dos Santos P, Goldman BS, Ertesvag H, Espin G, Rubio LM *et al*. Genome sequence of Azotobacter vinelandii, an obligate aerobe specialized to support diverse anaerobic metabolic processes*.* *J Bacteriol* 191(14), 4534-4545 (2009).

24. Mishra S, Imlay JA. An anaerobic bacterium, Bacteroides thetaiotaomicron, uses a consortium of enzymes to scavenge hydrogen peroxide*.* *Mol Microbiol* 90(6), 1356-1371 (2013).

25. Tamas I, Dedysh SN, Liesack W, Stott MB, Alam M, Murrell JC *et al*. Complete genome sequence of Beijerinckia indica subsp. indica*.* *J Bacteriol* 192(17), 4532-4533 (2010).

26. Land M, Pukall R, Abt B, Goker M, Rohde M, Glavina Del Rio T *et al*. Complete genome sequence of Beutenbergia cavernae type strain (HKI 0122)*.* *Stand Genomic Sci* 1(1), 21-28 (2009).

27. Schlesner H. *Bergey's Manual of Systematics of Archaea and Bacteria*. 1 (2015).

28. Halling SM, Peterson-Burch BD, Bricker BJ, Zuerner RL, Qing Z, Li LL *et al*. Completion of the genome sequence of Brucella abortus and comparison to the highly similar genomes of Brucella melitensis and Brucella suis*.* *J Bacteriol* 187(8), 2715-2726 (2005).

29. Kaestli M, Harrington G, Mayo M, Chatfield MD, Harrington I, Hill A *et al*. What drives the occurrence of the melioidosis bacterium Burkholderia pseudomallei in domestic gardens? *PLoS Negl Trop Dis* 9(3), e0003635 (2015).

30. Sekiguchi Y, Yamada T, Hanada S, Ohashi A, Harada H, Kamagata Y. Anaerolinea thermophila gen. nov., sp. nov. and Caldilinea aerophila gen. nov., sp. nov., novel filamentous thermophiles that represent a previously uncultured lineage of the domain Bacteria at the subphylum level*.* *Int J Syst Evol Microbiol* 53(Pt 6), 1843-1851 (2003).

31. Iino T, Nakagawa T, Mori K, Harayama S, Suzuki K. Calditerrivibrio nitroreducens gen. nov., sp. nov., a thermophilic, nitrate-reducing bacterium isolated from a terrestrial hot spring in Japan*.* *Int J Syst Evol Microbiol* 58(Pt 7), 1675-1679 (2008).

32. Anil Kumar P, Srinivas TN, Madhu S, Sravan R, Singh S, Naqvi SW *et al*. Cecembia lonarensis gen. nov., sp. nov., a haloalkalitolerant bacterium of the family Cyclobacteriaceae, isolated from a haloalkaline lake and emended descriptions of the genera Indibacter, Nitritalea and Belliella*.* *Int J Syst Evol Microbiol* 62(Pt 9), 2252-2258 (2012).

33. Pati A, Abt B, Teshima H, Nolan M, Lapidus A, Lucas S *et al*. Complete genome sequence of Cellulophaga lytica type strain (LIM-21)*.* *Stand Genomic Sci* 4(2), 221-232 (2011).

34. Sharma A, Gilbert JA, Lal R. (Meta)genomic insights into the pathogenome of Cellulosimicrobium cellulans*.* *Sci Rep* 6 25527 (2016).

35. Kudirkiene E, Hansen MJ, Bojesen AM. Draft Genome Sequence of Chelonobacter oris Strain 1662T, Associated with Respiratory Disease in Hermann's Tortoises*.* *Genome Announc* 2(6), (2014).

36. Mckee LS, Brumer H. Growth of Chitinophaga pinensis on Plant Cell Wall Glycans and Characterisation of a Glycoside Hydrolase Family 27 beta-l-Arabinopyranosidase Implicated in Arabinogalactan Utilisation*.* *PLoS One* 10(10), e0139932 (2015).

37. Wahlund TM, Madigan MT. Nitrogen fixation by the thermophilic green sulfur bacterium Chlorobium tepidum*.* *J Bacteriol* 175(2), 474-478 (1993).

38. Herter S, Farfsing J, Gad'on N, Rieder C, Eisenreich W, Bacher A *et al*. Autotrophic CO(2) fixation by Chloroflexus aurantiacus: study of glyoxylate formation and assimilation via the 3-hydroxypropionate cycle*.* *J Bacteriol* 183(14), 4305-4316 (2001).

39. Gibson J, Pfennig N, Waterbury JB. Chloroherpeton thalassium gen. nov. et spec. nov., a non-filamentous, flexing and gliding green sulfur bacterium*.* *Arch Microbiol* 138(2), 96-101 (1984).

40. Kumar MR. Chromobacterium violaceum: A rare bacterium isolated from a wound over the scalp*.* *Int J Appl Basic Med Res* 2(1), 70-72 (2012).

41. Bahl J, Lau MC, Smith GJ, Vijaykrishna D, Cary SC, Lacap DC *et al*. Ancient origins determine global biogeography of hot and cold desert cyanobacteria*.* *Nat Commun* 2 163 (2011).

42. Benmalek Y, Cayol JL, Bouanane NA, Hacene H, Fauque G, Fardeau ML. Chryseobacterium solincola sp. nov., isolated from soil*.* *Int J Syst Evol Microbiol* 60(Pt 8), 1876-1880 (2010).

43. Lee KC, Dunfield PF, Morgan XC, Crowe MA, Houghton KM, Vyssotski M *et al*. Chthonomonas calidirosea gen. nov., sp. nov., an aerobic, pigmented, thermophilic micro-organism of a novel bacterial class, Chthonomonadetes classis nov., of the newly described phylum Armatimonadetes originally designated candidate division OP10*.* *Int J Syst Evol Microbiol* 61(Pt 10), 2482-2490 (2011).

44. Siegesmund MA, Johansen JR, Karsten U, Friedl T. Coleofasciculus Gen. Nov. (Cyanobacteria): Morphological and Molecular Criteria for Revision of the Genus Microcoleus Gomont(1)*.* *J Phycol* 44(6), 1572-1585 (2008).

45. Monciardini P, Cavaletti L, Schumann P, Rohde M, Donadio S. Conexibacter woesei gen. nov., sp. nov., a novel representative of a deep evolutionary line of descent within the class Actinobacteria*.* *Int J Syst Evol Microbiol* 53(Pt 2), 569-576 (2003).

46. Mavromatis K, Abt B, Brambilla E, Lapidus A, Copeland A, Deshpande S *et al*. Complete genome sequence of Coraliomargarita akajimensis type strain (04OKA010-24)*.* *Stand Genomic Sci* 2(3), 290-299 (2010).

47. Vazquez G. *The role of algal mats on community succession in dunes and dune slacks*. Spinger, Berlin, Heidelberg. 171, (2008).

48. Kwon SK, Lee HG, Kwak MJ, Kim JF. Genome sequence of the marine flavobacterium Croceitalea dokdonensis DOKDO 023 that contains proton- and sodium-pumping rhodopsins*.* *Mar Genomics* 26 1-3 (2016).

49. Bench SR, Ilikchyan IN, Tripp HJ, Zehr JP. Two Strains of Crocosphaera watsonii with Highly Conserved Genomes are Distinguished by Strain-Specific Features*.* *Front Microbiol* 2 261 (2011).

50. Winckelmann D, Bleeke F, Bergmann P, Klock G. Growth of Cyanobacterium aponinum influenced by increasing salt concentrations and temperature*.* *3 Biotech* 5(3), 253-260 (2015).

51. Turner S, Huang T, Chaw S. Molecular phylogeny of nitrogen-fixing unicellular cyanobacteria*.* *Bot Bull Acad Sin* 42 6 (2001).

52. Lima AR, Siqueira AS, Dos Santos BG, Da Silva FD, Lima CP, Cardoso JF *et al*. Draft Genome Sequence of the Brazilian Cyanobium sp. Strain CACIAM 14*.* *Genome Announc* 2(4), (2014).

53. Ying JY, Wang BJ, Yang SS, Liu SJ. Cyclobacterium lianum sp. nov., a marine bacterium isolated from sediment of an oilfield in the South China Sea, and emended description of the genus Cyclobacterium*.* *Int J Syst Evol Microbiol* 56(Pt 12), 2927-2930 (2006).

54. Carr NG, Whitton BA. *The Biology of Blue-Green Algae*. University of California Press, Berkeley. (1973).

55. Verma JP, Martin HH. Chemistry and ultrastructure of surface layers in primitive myxobacteria: Cytophaga hutchinsonii and Sporocytophaga myxococcoides*.* *Folia Microbiol (Praha)* 12(3), 248-254 (1967).

56. Salinero KK, Keller K, Feil WS, Feil H, Trong S, Di Bartolo G *et al*. Metabolic analysis of the soil microbe Dechloromonas aromatica str. RCB: indications of a surprisingly complex life-style and cryptic anaerobic pathways for aromatic degradation*.* *BMC Genomics* 10 351 (2009).

57. Byrne-Bailey KG, Coates JD. Complete genome sequence of the anaerobic perchlorate-reducing bacterium Azospira suillum strain PS*.* *J Bacteriol* 194(10), 2767-2768 (2012).

58. Myhr S, Torsvik T. Denitrovibrio acetiphilus, a novel genus and species of dissimilatory nitrate-reducing bacterium isolated from an oil reservoir model column*.* *Int J Syst Evol Microbiol* 50 Pt 4 1611-1619 (2000).

59. Bonch-Osmolovskaya EA, Sokolova TG, Kostrikina NA, Zavarzin GA. Desulfurella acetivorans gen. nov. and sp. nov. —a new thermophilic sulfur-reducing eubacterium*.* *Arch Microbiol* 153(2), 5 (1989).

60. Bini E, Rauschenbach I, Narasingarao P, Starovoytov V, Hauser L, Jeffries CD *et al*. Complete genome sequence of Desulfurispirillum indicum strain S5(T)*.* *Stand Genomic Sci* 5(3), 371-378 (2011).

61. Magot M, Ravot G, Campaignolle X, Ollivier B, Patel BK, Fardeau ML *et al*. Dethiosulfovibrio peptidovorans gen. nov., sp. nov., a new anaerobic, slightly halophilic, thiosulfate-reducing bacterium from corroding offshore oil wells*.* *Int J Syst Bacteriol* 47(3), 818-824 (1997).

62. Nakagawa Y, Sakane T, Yokota A. Transfer of "Pseudomonas riboflavina" (Foster 1944), a gram-negative, motile rod with long-chain 3-hydroxy fatty acids, to Devosia riboflavina gen. nov., sp. nov., nom. rev*.* *Int J Syst Bacteriol* 46(1), 16-22 (1996).

63. Morotomi M, Nagai F, Sakon H, Tanaka R. Dialister succinatiphilus sp. nov. and Barnesiella intestinihominis sp. nov., isolated from human faeces*.* *Int J Syst Evol Microbiol* 58(Pt 12), 2716-2720 (2008).

64. Du ZJ, Wang Y, Dunlap C, Rooney AP, Chen GJ. Draconibacterium orientale gen. nov., sp. nov., isolated from two distinct marine environments, and proposal of Draconibacteriaceae fam. nov*.* *Int J Syst Evol Microbiol* 64(Pt 5), 1690-1696 (2014).

65. Chelius MK, Triplett EW. Dyadobacter fermentans gen. nov., sp. nov., a novel gram-negative bacterium isolated from surface-sterilized Zea mays stems*.* *Int J Syst Evol Microbiol* 50 Pt 2 751-758 (2000).

66. Vaughan LB, Forbes BA. Photo quiz: A 50-year-old with a 2-day history of right upper quadrant tenderness and septicemia caused by a Gram-negative organism*.* *J Clin Microbiol* 52(6), 1811, 2287 (2014).

67. Nedashkovskaya OI, Kim SB, Hoste B, Shin DS, Beleneva IA, Vancanneyt M *et al*. Echinicola vietnamensis sp. nov., a member of the phylum Bacteroidetes isolated from seawater*.* *Int J Syst Evol Microbiol* 57(Pt 4), 761-763 (2007).

68. Perrin A, Larsonneur E, Nicholson AC, Edwards DJ, Gundlach KM, Whitney AM *et al*. Evolutionary dynamics and genomic features of the Elizabethkingia anophelis 2015 to 2016 Wisconsin outbreak strain*.* *Nat Commun* 8 15483 (2017).

69. Saha P, Chakrabarti T. Emticicia oligotrophica gen. nov., sp. nov., a new member of the family 'Flexibacteraceae', phylum Bacteroidetes*.* *Int J Syst Evol Microbiol* 56(Pt 5), 991-995 (2006).

70. Deangelis KM, Sharma D, Varney R, Simmons B, Isern NG, Markilllie LM *et al*. Evidence supporting dissimilatory and assimilatory lignin degradation in Enterobacter lignolyticus SCF1*.* *Front Microbiol* 4 280 (2013).

71. Wang Y, Zhang R, Zheng Q, Jiao N. Draft Genome Sequences of Two Marine Phototrophic Bacteria, Erythrobacter longus Strain DSM 6997 and Erythrobacter litoralis Strain DSM 8509*.* *Genome Announc* 2(4), (2014).

72. Nolan M, Sikorski J, Davenport K, Lucas S, Del Rio TG, Tice H *et al*. Complete genome sequence of Ferrimonas balearica type strain (PAT)*.* *Stand Genomic Sci* 3(2), 174-182 (2010).

73. Filippini M, Svercel M, Laczko E, Kaech A, Ziegler U, Bagheri HC. Fibrella aestuarina gen. nov., sp. nov., a filamentous bacterium of the family Cytophagaceae isolated from a tidal flat, and emended description of the genus Rudanella Weon et al. 2008*.* *Int J Syst Evol Microbiol* 61(Pt 1), 184-189 (2011).

74. Filippini M, Kaech A, Ziegler U, Bagheri HC. Fibrisoma limi gen. nov., sp. nov., a filamentous bacterium isolated from tidal flats*.* *Int J Syst Evol Microbiol* 61(Pt 6), 1418-1424 (2011).

75. Im WT, Hu ZY, Kim KH, Rhee SK, Meng H, Lee ST *et al*. Description of Fimbriimonas ginsengisoli gen. nov., sp. nov. within the Fimbriimonadia class nov., of the phylum Armatimonadetes*.* *Antonie Van Leeuwenhoek* 102(2), 307-317 (2012).

76. Lee HJ, Jeong SE, Cho MS, Kim S, Lee SS, Lee BH *et al*. Flavihumibacter solisilvae sp. nov., isolated from forest soil*.* *Int J Syst Evol Microbiol* 64(Pt 8), 2897-2901 (2014).

77. Van Trappen S, Vandecandelaere I, Mergaert J, Swings J. Flavobacterium degerlachei sp. nov., Flavobacterium frigoris sp. nov. and Flavobacterium micromati sp. nov., novel psychrophilic bacteria isolated from microbial mats in Antarctic lakes*.* *Int J Syst Evol Microbiol* 54(Pt 1), 85-92 (2004).

78. Alloisio N, Queiroux C, Fournier P, Pujic P, Normand P, Vallenet D *et al*. The Frankia alni symbiotic transcriptome*.* *Mol Plant Microbe Interact* 23(5), 593-607 (2010).

79. Nupur, Sharma S, Kumar Singh P, Suresh K, Anil Kumar P. Fulvivirga imtechensis sp. nov., a member of the phylum Bacteroidetes*.* *Int J Syst Evol Microbiol* 62(Pt 9), 2213-2217 (2012).

80. Li C, Lai Q, Fu Y, Chen S, Shao Z. Galbibacter marinus sp. nov., isolated from deep-sea sediment*.* *Int J Syst Evol Microbiol* 63(Pt 4), 1427-1430 (2013).

81. Lai Q, Wang L, Wang W, Shao Z. Genome sequence of Gallaecimonas xiamenensis type strain 3-C-1*.* *J Bacteriol* 194(24), 6937 (2012).

82. Zhang H, Sekiguchi Y, Hanada S, Hugenholtz P, Kim H, Kamagata Y *et al*. Gemmatimonas aurantiaca gen. nov., sp. nov., a gram-negative, aerobic, polyphosphate-accumulating micro-organism, the first cultured representative of the new bacterial phylum Gemmatimonadetes phyl. nov*.* *Int J Syst Evol Microbiol* 53(Pt 4), 1155-1163 (2003).

83. Pascual J, Garcia-Lopez M, Bills GF, Genilloud O. Longimicrobium terrae gen. nov., sp. nov., an oligotrophic bacterium of the under-represented phylum Gemmatimonadetes isolated through a system of miniaturized diffusion chambers*.* *Int J Syst Evol Microbiol* 66(5), 1976-1985 (2016).

84. Coates JD, Phillips EJ, Lonergan DJ, Jenter H, Lovley DR. Isolation of Geobacter species from diverse sedimentary environments*.* *Appl Environ Microbiol* 62(5), 1531-1536 (1996).

85. Van Trappen S, Vandecandelaere I, Mergaert J, Swings J. Gillisia limnaea gen. nov., sp. nov., a new member of the family Flavobacteriaceae isolated from a microbial mat in Lake Fryxell, Antarctica*.* *Int J Syst Evol Microbiol* 54(Pt 2), 445-448 (2004).

86. Tsuchiya T, Takaichi S, Misawa N, Maoka T, Miyashita H, Mimuro M. The cyanobacterium Gloeobacter violaceus PCC 7421 uses bacterial-type phytoene desaturase in carotenoid biosynthesis*.* *FEBS Lett* 579(10), 2125-2129 (2005).

87. Arencibia AD, Vinagre F, Estevez Y, Bernal A, Perez J, Cavalcanti J *et al*. Gluconacetobacter diazotrophicus Elicits a Sugarcane Defense Response Against a Pathogenic Bacteria Xanthomonas albilineans*.* *Plant Signal Behav* 1(5), 265-273 (2006).

88. Panschin I, Becher M, Verbarg S, Sproer C, Rohde M, Schuler M *et al*. Description of Gramella forsetii sp. nov., a marine Flavobacteriaceae isolated from North Sea water, and emended description of Gramella gaetbulicola Cho et al. 2011*.* *Int J Syst Evol Microbiol* doi:10.1099/ijsem.0.001700 (2016).

89. Fudou R, Jojima Y, Iizuka T, Yamanaka S. Haliangium ochraceum gen. nov., sp. nov. and Haliangium tepidum sp. nov.: novel moderately halophilic myxobacteria isolated from coastal saline environments*.* *J Gen Appl Microbiol* 48(2), 109-116 (2002).

90. Daligault H, Lapidus A, Zeytun A, Nolan M, Lucas S, Del Rio TG *et al*. Complete genome sequence of Haliscomenobacter hydrossis type strain (O)*.* *Stand Genomic Sci* 4(3), 352-360 (2011).

91. Vikromvarasiria N, Pisutpaisal N. Potential Application of Halothiobacillus Neapolitanus for Hydrogen Sulfide Removal in Biogas*.* *Energy Procedia* 61 5 (2014).

92. Singh D, Chandrababunaidu MM, Panda A, Sen D, Bhattacharyya S, Adhikary SP *et al*. Draft Genome Sequence of Cyanobacterium Hassallia byssoidea Strain VB512170, Isolated from Monuments in India*.* *Genome Announc* 3(2), (2015).

93. Kiss H, Nett M, Domin N, Martin K, Maresca JA, Copeland A *et al*. Complete genome sequence of the filamentous gliding predatory bacterium Herpetosiphon aurantiacus type strain (114-95(T))*.* *Stand Genomic Sci* 5(3), 356-370 (2011).

94. Chertkov O, Brown PJ, Kysela DT, De Pedro MA, Lucas S, Copeland A *et al*. Complete genome sequence of Hirschia baltica type strain (IFAM 1418(T))*.* *Stand Genomic Sci* 5(3), 287-297 (2011).

95. Nadmid S, Plaza A, Lauro G, Garcia R, Bifulco G, Muller R. Hyalachelins A-C, unusual siderophores isolated from the terrestrial myxobacterium Hyalangium minutum*.* *Org Lett* 16(16), 4130-4133 (2014).

96. Nishihara H, Yaguchi T, Chung SY, Suzuki K, Yanagi M, Yamasato K *et al*. Phylogenetic position of an obligately chemoautotrophic, marine hydrogen-oxidizing bacterium, Hydrogenovibrio marinus, on the basis of 16S rRNA gene sequences and two form I RuBisCO gene sequences*.* *Arch Microbiol* 169(4), 364-368 (1998).

97. Iino T, Mori K, Uchino Y, Nakagawa T, Harayama S, Suzuki K. Ignavibacterium album gen. nov., sp. nov., a moderately thermophilic anaerobic bacterium isolated from microbial mats at a terrestrial hot spring and proposal of Ignavibacteria classis nov., for a novel lineage at the periphery of green sulfur bacteria*.* *Int J Syst Evol Microbiol* 60(Pt 6), 1376-1382 (2010).

98. Sikorski J, Chertkov O, Lapidus A, Nolan M, Lucas S, Del Rio TG *et al*. Complete genome sequence of Ilyobacter polytropus type strain (CuHbu1)*.* *Stand Genomic Sci* 3(3), 304-314 (2010).

99. Groth I, Schumann P, Schutze B, Gonzalez JM, Laiz L, Saiz-Jimenez C *et al*. Isoptericola hypogeus sp. nov., isolated from the Roman catacomb of Domitilla*.* *Int J Syst Evol Microbiol* 55(Pt 4), 1715-1719 (2005).

100. Graupner K, Lackner G, Hertweck C. Genome Sequence of Mushroom Soft-Rot Pathogen Janthinobacterium agaricidamnosum*.* *Genome Announc* 3(2), (2015).

101. Bagley ST. Habitat association of Klebsiella species*.* *Infect Control* 6(2), 52-58 (1985).

102. Pukall R, Lapidus A, Glavina Del Rio T, Copeland A, Tice H, Cheng JF *et al*. Complete genome sequence of Kribbella flavida type strain (IFO 14399)*.* *Stand Genomic Sci* 2(2), 186-193 (2010).

103. Biebl H, Pukall R, Lunsdorf H, Schulz S, Allgaier M, Tindall BJ *et al*. Description of Labrenzia alexandrii gen. nov., sp. nov., a novel alphaproteobacterium containing bacteriochlorophyll a, and a proposal for reclassification of Stappia aggregata as Labrenzia aggregata comb. nov., of Stappia marina as Labrenzia marina comb. nov. and of Stappia alba as Labrenzia alba comb. nov., and emended descriptions of the genera Pannonibacter, Stappia and Roseibium, and of the species Roseibium denhamense and Roseibium hamelinense*.* *Int J Syst Evol Microbiol* 57(Pt 5), 1095-1107 (2007).

104. Curreem SO, Teng JL, Tse H, Yuen KY, Lau SK, Woo PC. General metabolism of Laribacter hongkongensis: a genome-wide analysis*.* *Cell Biosci* 1(1), 16 (2011).

105. Pinhassi J, Bowman JP, Nedashkovskaya OI, Lekunberri I, Gomez-Consarnau L, Pedros-Alio C. Leeuwenhoekiella blandensis sp. nov., a genome-sequenced marine member of the family Flavobacteriaceae*.* *Int J Syst Evol Microbiol* 56(Pt 7), 1489-1493 (2006).

106. Takeda M, Makita H, Ohno K, Nakahara Y, Koizumi J. Structural analysis of the sheath of a sheathed bacterium, Leptothrix cholodnii*.* *Int J Biol Macromol* 37(1-2), 92-98 (2005).

107. Riedel T, Fiebig A, Petersen J, Gronow S, Kyrpides NC, Goker M *et al*. Genome sequence of the Litoreibacter arenae type strain (DSM 19593(T)), a member of the Roseobacter clade isolated from sea sand*.* *Stand Genomic Sci* 9(1), 117-127 (2013).

108. Chandrababunaidu MM, Sen D, Tripathy S. Draft Genome Sequence of Filamentous Marine Cyanobacterium Lyngbya confervoides Strain BDU141951*.* *Genome Announc* 3(2), (2015).

109. Bhumika V, Srinivas TN, Ravinder K, Kumar PA. Mariniradius saccharolyticus gen. nov., sp. nov., a member of the family Cyclobacteriaceae isolated from marine aquaculture pond water, and emended descriptions of the genus Aquiflexum and Aquiflexum balticum*.* *Int J Syst Evol Microbiol* 63(Pt 6), 2088-2094 (2013).

110. Gauthier MJ, Lafay B, Christen R, Fernandez L, Acquaviva M, Bonin P *et al*. Marinobacter hydrocarbonoclasticus gen. nov., sp. nov., a new, extremely halotolerant, hydrocarbon-degrading marine bacterium*.* *Int J Syst Bacteriol* 42(4), 568-576 (1992).

111. Solano F, Lucas-Elio P, Fernandez E, Sanchez-Amat A. Marinomonas mediterranea MMB-1 transposon mutagenesis: isolation of a multipotent polyphenol oxidase mutant*.* *J Bacteriol* 182(13), 3754-3760 (2000).

112. Pagani I, Chertkov O, Lapidus A, Lucas S, Del Rio TG, Tice H *et al*. Complete genome sequence of Marivirga tractuosa type strain (H-43)*.* *Stand Genomic Sci* 4(2), 154-162 (2011).

113. Khan A, Khan H, Chung EJ, Hossain MT, Chung YR. Complete Genome Sequence of Martelella endophytica YC6887, Which Has Antifungal Activity Associated with a Halophyte*.* *Genome Announc* 3(3), (2015).

114. La Scola B, Birtles RJ, Mallet MN, Raoult D. Massilia timonae gen. nov., sp. nov., isolated from blood of an immunocompromised patient with cerebellar lesions*.* *J Clin Microbiol* 36(10), 2847-2852 (1998).

115. Nechushtai R, Muster P, Binder A, Liveanu V, Nelson N. Photosystem I reaction center from the thermophilic cyanobacterium Mastigocladus laminosus*.* *Proc Natl Acad Sci U S A* 80(5), 1179-1183 (1983).

116. Nakatsu CH, Hristova K, Hanada S, Meng XY, Hanson JR, Scow KM *et al*. Methylibium petroleiphilum gen. nov., sp. nov., a novel methyl tert-butyl ether-degrading methylotroph of the Betaproteobacteria*.* *Int J Syst Evol Microbiol* 56(Pt 5), 983-989 (2006).

117. Chistoserdova L, Lapidus A, Han C, Goodwin L, Saunders L, Brettin T *et al*. Genome of Methylobacillus flagellatus, molecular basis for obligate methylotrophy, and polyphyletic origin of methylotrophy*.* *J Bacteriol* 189(11), 4020-4027 (2007).

118. Eevers N, Van Hamme JD, Bottos EM, Weyens N, Vangronsveld J. Draft Genome Sequence of Methylobacterium radiotolerans, a DDE-Degrading and Plant Growth-Promoting Strain Isolated from Cucurbita pepo*.* *Genome Announc* 3(3), (2015).

119. Dunfield PF, Khmelenina VN, Suzina NE, Trotsenko YA, Dedysh SN. Methylocella silvestris sp. nov., a novel methanotroph isolated from an acidic forest cambisol*.* *Int J Syst Evol Microbiol* 53(Pt 5), 1231-1239 (2003).

120. Kleiveland CR, Hult LT, Kuczkowska K, Jacobsen M, Lea T, Pope PB. Draft genome sequence of the methane-oxidizing bacterium Methylococcus capsulatus (Texas)*.* *J Bacteriol* 194(23), 6626 (2012).

121. Boden R, Cunliffe M, Scanlan J, Moussard H, Kits KD, Klotz MG *et al*. Complete genome sequence of the aerobic marine methanotroph Methylomonas methanica MC09*.* *J Bacteriol* 193(24), 7001-7002 (2011).

122. Kalyuzhnaya MG, Beck DA, Vorobev A, Smalley N, Kunkel DD, Lidstrom ME *et al*. Novel methylotrophic isolates from lake sediment, description of Methylotenera versatilis sp. nov. and emended description of the genus Methylotenera*.* *Int J Syst Evol Microbiol* 62(Pt 1), 106-111 (2012).

123. Kalyuzhnaya MG, De Marco P, Bowerman S, Pacheco CC, Lara JC, Lidstrom ME *et al*. Methyloversatilis universalis gen. nov., sp. nov., a novel taxon within the Betaproteobacteria represented by three methylotrophic isolates*.* *Int J Syst Evol Microbiol* 56(Pt 11), 2517-2522 (2006).

124. Lapidus A, Clum A, Labutti K, Kaluzhnaya MG, Lim S, Beck DA *et al*. Genomes of three methylotrophs from a single niche reveal the genetic and metabolic divergence of the methylophilaceae*.* *J Bacteriol* 193(15), 3757-3764 (2011).

125. Dang TC, Fujii M, Rose AL, Bligh M, Waite TD. Characteristics of the freshwater cyanobacterium Microcystis aeruginosa grown in iron-limited continuous culture*.* *Appl Environ Microbiol* 78(5), 1574-1583 (2012).

126. Trujillo ME, Riesco R, Benito P, Carro L. Endophytic Actinobacteria and the Interaction of Micromonospora and Nitrogen Fixing Plants*.* *Front Microbiol* 6 1341 (2015).

127. Hopkinson BM, Roe KL, Barbeau KA. Heme uptake by Microscilla marina and evidence for heme uptake systems in the genomes of diverse marine bacteria*.* *Appl Environ Microbiol* 74(20), 6263-6270 (2008).

128. Reeve W, Ardley J, Tian R, De Meyer S, Terpolilli J, Melino V *et al*. Genome sequence of the Listia angolensis microsymbiont Microvirga lotononidis strain WSM3557(T.)*.* *Stand Genomic Sci* 9(3), 540-550 (2014).

129. Engene N, Rottacker EC, Kastovsky J, Byrum T, Choi H, Ellisman MH *et al*. Moorea producens gen. nov., sp. nov. and Moorea bouillonii comb. nov., tropical marine cyanobacteria rich in bioactive secondary metabolites*.* *Int J Syst Evol Microbiol* 62(Pt 5), 1171-1178 (2012).

130. Pankratov TA, Tindall BJ, Liesack W, Dedysh SN. Mucilaginibacter paludis gen. nov., sp. nov. and Mucilaginibacter gracilis sp. nov., pectin-, xylan- and laminarin-degrading members of the family Sphingobacteriaceae from acidic Sphagnum peat bog*.* *Int J Syst Evol Microbiol* 57(Pt 10), 2349-2354 (2007).

131. Lee LH, Zainal N, Azman AS, Mutalib NS, Hong K, Chan KG. Mumia flava gen. nov., sp. nov., an actinobacterium of the family Nocardioidaceae*.* *Int J Syst Evol Microbiol* 64(Pt 5), 1461-1467 (2014).

132. Vos M, Velicer GJ. Genetic population structure of the soil bacterium Myxococcus xanthus at the centimeter scale*.* *Appl Environ Microbiol* 72(5), 3615-3625 (2006).

133. Arahal DR, Lekunberri I, Gonzalez JM, Pascual J, Pujalte MJ, Pedros-Alio C *et al*. Neptuniibacter caesariensis gen. nov., sp. nov., a novel marine genome-sequenced gammaproteobacterium*.* *Int J Syst Evol Microbiol* 57(Pt 5), 1000-1006 (2007).

134. Weon HY, Kim BY, Joa JH, Kwon SW, Kim WG, Koo BS. Niabella soli sp. nov., isolated from soil from Jeju Island, Korea*.* *Int J Syst Evol Microbiol* 58(Pt 2), 467-469 (2008).

135. Weon HY, Kim BY, Yoo SH, Lee SY, Kwon SW, Go SJ *et al*. Niastella koreensis gen. nov., sp. nov. and Niastella yeongjuensis sp. nov., novel members of the phylum Bacteroidetes, isolated from soil cultivated with Korean ginseng*.* *Int J Syst Evol Microbiol* 56(Pt 8), 1777-1782 (2006).

136. Nakagawa S, Takai K, Inagaki F, Horikoshi K, Sako Y. Nitratiruptor tergarcus gen. nov., sp. nov. and Nitratifractor salsuginis gen. nov., sp. nov., nitrate-reducing chemolithoautotrophs of the epsilon-Proteobacteria isolated from a deep-sea hydrothermal system in the Mid-Okinawa Trough*.* *Int J Syst Evol Microbiol* 55(Pt 2), 925-933 (2005).

137. Mcgregor GB, Stewart I, Sendall BC, Sadler R, Reardon K, Carter S *et al*. First report of a toxic Nodularia spumigena (Nostocales/ Cyanobacteria) bloom in sub-tropical Australia. I. Phycological and public health investigations*.* *Int J Environ Res Public Health* 9(7), 2396-2411 (2012).

138. Moraes LE, Blow MJ, Hawley ER, Piao H, Kuo R, Chiniquy J *et al*. Resequencing and annotation of the Nostoc punctiforme ATTC 29133 genome: facilitating biofuel and high-value chemical production*.* *AMB Express* 7(1), 42 (2017).

139. Lai Q, Yuan J, Wu C, Shao Z. Oceanibaculum indicum gen. nov., sp. nov., isolated from deep seawater of the Indian Ocean*.* *Int J Syst Evol Microbiol* 59(Pt 7), 1733-1737 (2009).

140. Wagner-Dobler I, Rheims H, Felske A, El-Ghezal A, Flade-Schroder D, Laatsch H *et al*. Oceanibulbus indolifex gen. nov., sp. nov., a North Sea alphaproteobacterium that produces bioactive metabolites*.* *Int J Syst Evol Microbiol* 54(Pt 4), 1177-1184 (2004).

141. Hagiya H, Ohnishi K, Maki M, Watanabe N, Murase T. Clinical characteristics of Ochrobactrum anthropi bacteremia*.* *J Clin Microbiol* 51(4), 1330-1333 (2013).

142. Chin KJ, Liesack W, Janssen PH. Opitutus terrae gen. nov., sp. nov., to accommodate novel strains of the division 'Verrucomicrobia' isolated from rice paddy soil*.* *Int J Syst Evol Microbiol* 51(Pt 6), 1965-1968 (2001).

143. Van Empel PC, Hafez HM. Ornithobacterium rhinotracheale: A review*.* *Avian Pathol* 28(3), 217-227 (1999).

144. Martins MD, Branco LH. Potamolinea gen. nov. (Oscillatoriales, Cyanobacteria): a phylogenetically and ecologically coherent cyanobacterial genus*.* *Int J Syst Evol Microbiol* 66(9), 3632-3641 (2016).

145. Coutinho TA, Venter SN. Pantoea ananatis: an unconventional plant pathogen*.* *Mol Plant Pathol* 10(3), 325-335 (2009).

146. Schleheck D, Weiss M, Pitluck S, Bruce D, Land ML, Han S *et al*. Complete genome sequence of Parvibaculum lavamentivorans type strain (DS-1(T))*.* *Stand Genomic Sci* 5(3), 298-310 (2011).

147. Almeida B, Vaz-Moreira I, Schumann P, Nunes OC, Carvalho G, Barreto Crespo MT. Patulibacter medicamentivorans sp. nov., isolated from activated sludge of a wastewater treatment plant*.* *Int J Syst Evol Microbiol* 63(Pt 7), 2588-2593 (2013).

148. Steyn PL, Segers P, Vancanneyt M, Sandra P, Kersters K, Joubert JJ. Classification of heparinolytic bacteria into a new genus, Pedobacter, comprising four species: Pedobacter heparinus comb. nov., Pedobacter piscium comb. nov., Pedobacter africanus sp. nov. and Pedobacter saltans sp. nov. proposal of the family Sphingobacteriaceae fam. nov*.* *Int J Syst Bacteriol* 48 Pt 1 165-177 (1998).

149. Huo YY, Cheng H, Han XF, Jiang XW, Sun C, Zhang XQ *et al*. Complete genome sequence of Pelagibacterium halotolerans B2(T)*.* *J Bacteriol* 194(1), 197-198 (2012).

150. Overmann J, Pfennig N. Pelodictyon phaeoclathratiforme sp. nov., a new brown-colored member of the Chlorobiaceae forming net-like colonies*.* *Arch Microbiol* 152(4), 6 (1989).

151. Chen Z, Lei X, Lai Q, Li Y, Zhang B, Zhang J *et al*. Phaeodactylibacter xiamenensis gen. nov., sp. nov., a member of the family Saprospiraceae isolated from the marine alga Phaeodactylum tricornutum*.* *Int J Syst Evol Microbiol* 64(Pt 10), 3496-3502 (2014).

152. Zhang K, Han W, Zhang R, Xu X, Pan Q, Hu X. Phenylobacterium zucineum sp. nov., a facultative intracellular bacterium isolated from a human erythroleukemia cell line K562*.* *Syst Appl Microbiol* 30(3), 207-212 (2007).

153. Chrismas NA, Barker G, Anesio AM, Sanchez-Baracaldo P. Genomic mechanisms for cold tolerance and production of exopolysaccharides in the Arctic cyanobacterium Phormidesmis priestleyi BC1401*.* *BMC Genomics* 17 533 (2016).

154. Vezzi A, Campanaro S, D'angelo M, Simonato F, Vitulo N, Lauro FM *et al*. Life at depth: Photobacterium profundum genome sequence and expression analysis*.* *Science* 307(5714), 1459-1461 (2005).

155. Clum A, Tindall BJ, Sikorski J, Ivanova N, Mavrommatis K, Lucas S *et al*. Complete genome sequence of Pirellula staleyi type strain (ATCC 27377)*.* *Stand Genomic Sci* 1(3), 308-316 (2009).

156. Scheuner C, Tindall BJ, Lu M, Nolan M, Lapidus A, Cheng JF *et al*. Complete genome sequence of Planctomyces brasiliensis type strain (DSM 5305(T)), phylogenomic analysis and reclassification of Planctomycetes including the descriptions of Gimesia gen. nov., Planctopirus gen. nov. and Rubinisphaera gen. nov. and emended descriptions of the order Planctomycetales and the family Planctomycetaceae*.* *Stand Genomic Sci* 9 10 (2014).

157. Tonk L, Visser PM, Christiansen G, Dittmann E, Snelder EO, Wiedner C *et al*. The microcystin composition of the cyanobacterium Planktothrix agardhii changes toward a more toxic variant with increasing light intensity*.* *Appl Environ Microbiol* 71(9), 5177-5181 (2005).

158. Kain KC, Kelly MT. Clinical features, epidemiology, and treatment of Plesiomonas shigelloides diarrhea*.* *J Clin Microbiol* 27(5), 998-1001 (1989).

159. Jeon CO, Park W, Ghiorse WC, Madsen EL. Polaromonas naphthalenivorans sp. nov., a naphthalene-degrading bacterium from naphthalene-contaminated sediment*.* *Int J Syst Evol Microbiol* 54(Pt 1), 93-97 (2004).

160. Nie Y, Tang YQ, Li Y, Chi CQ, Cai M, Wu XL. The genome sequence of Polymorphum gilvum SL003B-26A1(T) reveals its genetic basis for crude oil degradation and adaptation to the saline soil*.* *PLoS One* 7(2), e31261 (2012).

161. Coil DA, Flanagan JC, Stump A, Alexiev A, Lang JM, Eisen JA. Porphyrobacter mercurialis sp. nov., isolated from a stadium seat and emended description of the genus Porphyrobacter*.* *PeerJ* 3 e1400 (2015).

162. Gorlenko VM. A new phototrophic green sulphur bacterium. Prosthecochloris aestuarii nov. gen. nov. spec*.* *Z Allg Mikrobiol* 10(2), 147-149 (1970).

163. Grebers R, Wehmeyer U, Roggentin T, Schlesner H, Kolbel-Boelke J, Hirsch P. Deoxyribonucleic acid base composition of Prosthcomicrobium and Ancalomicrobium strains*.* *Microbiology* 35 10 (1985).

164. Finneran KT, Johnsen CV, Lovley DR. Rhodoferax ferrireducens sp. nov., a psychrotolerant, facultatively anaerobic bacterium that oxidizes acetate with the reduction of Fe(III)*.* *Int J Syst Evol Microbiol* 53(Pt 3), 669-673 (2003).

165. Schmidt M, Prieme A, Stougaard P. Rhodonellum psychrophilum gen. nov., sp. nov., a novel psychrophilic and alkaliphilic bacterium of the phylum Bacteroidetes isolated from Greenland*.* *Int J Syst Evol Microbiol* 56(Pt 12), 2887-2892 (2006).

166. Glockner FO, Kube M, Bauer M, Teeling H, Lombardot T, Ludwig W *et al*. Complete genome sequence of the marine planctomycete Pirellula sp. strain 1*.* *Proc Natl Acad Sci U S A* 100(14), 8298-8303 (2003).

167. Leavitt S, Ayroud M. Riemerella anatipestifer infection of domestic ducklings*.* *Can Vet J* 38(2), 113 (1997).

168. Choi DH, Noh JH, Lee CM, Rho S. Rubidibacter lacunae gen. nov., sp. nov., a unicellular, phycoerythrin-containing cyanobacterium isolated from seawater of Chuuk lagoon, Micronesia*.* *Int J Syst Evol Microbiol* 58(Pt 12), 2807-2811 (2008).

169. Copeland A, Zhang X, Misra M, Lapidus A, Nolan M, Lucas S *et al*. Complete genome sequence of the aquatic bacterium Runella slithyformis type strain (LSU 4(T))*.* *Stand Genomic Sci* 6(2), 145-154 (2012).

170. Liu QQ, Li J, Xiao D, Lu JX, Chen GJ, Du ZJ. Saccharicrinis marinus sp. nov., isolated from marine sediment*.* *Int J Syst Evol Microbiol* 65(10), 3427-3432 (2015).

171. Boubetra D, Zitouni A, Bouras N, Schumann P, Sproer C, Klenk HP *et al*. Saccharothrix tamanrassetensis sp. nov., an actinomycete isolated from Saharan soil*.* *Int J Syst Evol Microbiol* 65(Pt 4), 1316-1320 (2015).

172. Das A, Panda A, Singh D, Chandrababunaidu MM, Mishra GP, Bhan S *et al*. Deciphering the Genome Sequences of the Hydrophobic Cyanobacterium Scytonema tolypothrichoides VB-61278*.* *Genome Announc* 3(2), (2015).

173. Harmon-Smith M, Celia L, Chertkov O, Lapidus A, Copeland A, Glavina Del Rio T *et al*. Complete genome sequence of Sebaldella termitidis type strain (NCTC 11300)*.* *Stand Genomic Sci* 2(2), 220-227 (2010).

174. Grimont PaD, Grimont F, Starr MP. Serratia proteamaculans (Paine and Stansfield) comb. nov., a Senior Subjective Synonym of Serratia liquefaciens (Grimes and Hennerty) Bascomb et al. *Int J Syst Evol Microbiol* 28 8 (1978).

175. Beckwith CR, Edwards MJ, Lawes M, Shi L, Butt JN, Richardson DJ *et al*. Characterization of MtoD from Sideroxydans lithotrophicus: a cytochrome c electron shuttle used in lithoautotrophic growth*.* *Front Microbiol* 6 332 (2015).

176. Weon HY, Kim BY, Lee CM, Hong SB, Jeon YA, Koo BS *et al*. Solitalea koreensis gen. nov., sp. nov. and the reclassification of [Flexibacter] canadensis as Solitalea canadensis comb. nov*.* *Int J Syst Evol Microbiol* 59(Pt 8), 1969-1975 (2009).

177. Park S, Kim DH, Lee JH, Hur HG. Sphaerotilus natans encrusted with nanoball-shaped Fe(III) oxide minerals formed by nitrate-reducing mixotrophic Fe(II) oxidation*.* *FEMS Microbiol Ecol* 90(1), 68-77 (2014).

178. Gupta A, Logan J, Elhag N, Almond M. Sphingobacterium spiritivorum infection in a patient with end stage renal disease on haemodialysis*.* *Ann Clin Microbiol Antimicrob* 15 25 (2016).

179. Lail K, Sikorski J, Saunders E, Lapidus A, Glavina Del Rio T, Copeland A *et al*. Complete genome sequence of Spirosoma linguale type strain (1)*.* *Stand Genomic Sci* 2(2), 176-185 (2010).

180. Liu L, Gao P, Chen G, Wang L. Draft Genome Sequence of Cellulose-Digesting Bacterium Sporocytophaga myxococcoides PG-01*.* *Genome Announc* 2(6), (2014).

181. Shih PM, Wu D, Latifi A, Axen SD, Fewer DP, Talla E *et al*. Improving the coverage of the cyanobacterial phylum using diversity-driven genome sequencing*.* *Proc Natl Acad Sci U S A* 110(3), 1053-1058 (2013).

182. Heidelbach M, Skladny H, Schairer HU. Heat shock and development induce synthesis of a low-molecular-weight stress-responsive protein in the myxobacterium Stigmatella aurantiaca*.* *J Bacteriol* 175(22), 7479-7482 (1993).

183. Kojima H, Fukui M. Sulfuricella denitrificans gen. nov., sp. nov., a sulfur-oxidizing autotroph isolated from a freshwater lake*.* *Int J Syst Evol Microbiol* 60(Pt 12), 2862-2866 (2010).

184. Kodama Y, Watanabe K. Sulfuricurvum kujiense gen. nov., sp. nov., a facultatively anaerobic, chemolithoautotrophic, sulfur-oxidizing bacterium isolated from an underground crude-oil storage cavity*.* *Int J Syst Evol Microbiol* 54(Pt 6), 2297-2300 (2004).

185. Kojima H, Fukui M. Sulfuritalea hydrogenivorans gen. nov., sp. nov., a facultative autotroph isolated from a freshwater lake*.* *Int J Syst Evol Microbiol* 61(Pt 7), 1651-1655 (2011).

186. Yu J, Liberton M, Cliften PF, Head RD, Jacobs JM, Smith RD *et al*. Synechococcus elongatus UTEX 2973, a fast growing cyanobacterial chassis for biosynthesis using light and CO(2)*.* *Sci Rep* 5 8132 (2015).

187. Han C, Mwirichia R, Chertkov O, Held B, Lapidus A, Nolan M *et al*. Complete genome sequence of Syntrophobotulus glycolicus type strain (FlGlyR)*.* *Stand Genomic Sci* 4(3), 371-380 (2011).

188. Yukphan P, Malimas T, Muramatsu Y, Takahashi M, Kaneyasu M, Tanasupawat S *et al*. Tanticharoenia sakaeratensis gen. nov., sp. nov., a new osmotolerant acetic acid bacterium in the alpha-Proteobacteria*.* *Biosci Biotechnol Biochem* 72(3), 672-676 (2008).

189. Takeuchi M, Yamagishi T, Kamagata Y, Oshima K, Hattori M, Katayama T *et al*. Tepidicaulis marinus gen. nov., sp. nov., a marine bacterium that reduces nitrate to nitrous oxide under strictly microaerobic conditions*.* *Int J Syst Evol Microbiol* 65(Pt 6), 1749-1754 (2015).

190. Yakimov MM, Giuliano L, Denaro R, Crisafi E, Chernikova TN, Abraham WR *et al*. Thalassolituus oleivorans gen. nov., sp. nov., a novel marine bacterium that obligately utilizes hydrocarbons*.* *Int J Syst Evol Microbiol* 54(Pt 1), 141-148 (2004).

191. Chovatia M, Sikorski J, Schroder M, Lapidus A, Nolan M, Tice H *et al*. Complete genome sequence of Thermanaerovibrio acidaminovorans type strain (Su883)*.* *Stand Genomic Sci* 1(3), 254-261 (2009).

192. Prabha R, Singh DP, Rai A. Looking into the genome of Thermosynechococcus elongatus (thermophilic cyanobacteria) with codon selection and usage perspective*.* *Int J Bioinform Res Appl* 11(2), 130-141 (2015).

193. Sorokin DY, Lysenko AM, Mityushina LL, Tourova TP, Jones BE, Rainey FA *et al*. Thioalkalimicrobium aerophilum gen. nov., sp. nov. and Thioalkalimicrobium sibericum sp. nov., and Thioalkalivibrio versutus gen. nov., sp. nov., Thioalkalivibrio nitratis sp.nov., novel and Thioalkalivibrio denitrificancs sp. nov., novel obligately alkaliphilic and obligately chemolithoautotrophic sulfur-oxidizing bacteria from soda lakes*.* *Int J Syst Evol Microbiol* 51(Pt 2), 565-580 (2001).

194. Sorokin DY, Muntyan MS, Panteleeva AN, Muyzer G. Thioalkalivibrio sulfidiphilus sp. nov., a haloalkaliphilic, sulfur-oxidizing gammaproteobacterium from alkaline habitats*.* *Int J Syst Evol Microbiol* 62(Pt 8), 1884-1889 (2012).

195. Beller HR, Chain PS, Letain TE, Chakicherla A, Larimer FW, Richardson PM *et al*. The genome sequence of the obligately chemolithoautotrophic, facultatively anaerobic bacterium Thiobacillus denitrificans*.* *J Bacteriol* 188(4), 1473-1488 (2006).

196. Scott KM, Sievert SM, Abril FN, Ball LA, Barrett CJ, Blake RA *et al*. The genome of deep-sea vent chemolithoautotroph Thiomicrospira crunogena XCL-2*.* *PLoS Biol* 4(12), e383 (2006).

197. Wentzien SW, Sand W. Tetrathionate Disproportionation by Thiomonas intermedia K12*.* *Eng Life Sci* 4(1), 6 (2004).

198. Zaar A, Fuchs G, Golecki JR, Overmann J. A new purple sulfur bacterium isolated from a littoral microbial mat, Thiorhodococcus drewsii sp. nov*.* *Arch Microbiol* 179(3), 174-183 (2003).

199. Kawasaki Y, Endo T, Fujiwara A, Kondo K, Katahira M, Nittami T *et al*. Elongation pattern and fine structure of the sheaths formed by Thiothrix nivea and Thiothrix fructosivorans*.* *Int J Biol Macromol* 95 1280-1288 (2017).

200. Han JI, Choi HK, Lee SW, Orwin PM, Kim J, Laroe SL *et al*. Complete genome sequence of the metabolically versatile plant growth-promoting endophyte Variovorax paradoxus S110*.* *J Bacteriol* 193(5), 1183-1190 (2011).

201. Pinel N, Davidson SK, Stahl DA. Verminephrobacter eiseniae gen. nov., sp. nov., a nephridial symbiont of the earthworm Eisenia foetida (Savigny)*.* *Int J Syst Evol Microbiol* 58(Pt 9), 2147-2157 (2008).

202. Janssen DB, Scheper A, Dijkhuizen L, Witholt B. Degradation of halogenated aliphatic compounds by Xanthobacter autotrophicus GJ10*.* *Appl Environ Microbiol* 49(3), 673-677 (1985).

203. Rivas R, Sanchez M, Trujillo ME, Zurdo-Pineiro JL, Mateos PF, Martinez-Molina E *et al*. Xylanimonas cellulosilytica gen. nov., sp. nov., a xylanolytic bacterium isolated from a decayed tree (Ulmus nigra)*.* *Int J Syst Evol Microbiol* 53(Pt 1), 99-103 (2003).

204. Groisillier A, Labourel A, Michel G, Tonon T. The mannitol utilization system of the marine bacterium Zobellia galactanivorans*.* *Appl Environ Microbiol* 81(5), 1799-1812 (2015).

205. He MX, Wu B, Qin H, Ruan ZY, Tan FR, Wang JL *et al*. Zymomonas mobilis: a novel platform for future biorefineries*.* *Biotechnol Biofuels* 7 101 (2014).
