# Supplementary material for: Distribution Patterns of Polyphosphate Metabolism Pathway and Its Relationships With Bacterial Durability and Virulence
Source: Front Microbiol. 2018 Apr 24;9:782. doi: 10.3389/fmicb.2018.00782 (PMC5932413; doi:10.3389/fmicb.2018.00782)
Supplement: Supplementary file 3 [file Table_3.DOCX]

**Table S3. Lifestyle and environmental persistence of 14 bacterial species with complete loss of polyP metabolism pathway**

| **Genus** | **Species** | **UPI^#^** | **PS^#^** | **V^#^** | **Disease** | **Transmission** | **Lifestyle** | **P^#^** | **Ref.** |
| --- | --- | --- | --- | --- | --- | --- | --- | --- | --- |
| *Anaplasma^*^* | *phagocytophilum* | UP000001943 | 1330 | 23 | Anaplasmosis  (rarely fatal) | Tick-borne | Obligate intracellular parasitic | N/A | **[**[**1**](#_ENREF_1)**]** |
| *Buchnera* | *aphidicola* | UP000006811 | 359 | 12 | N/A | Aphid-associated | Intracellular symbiotic | N/A | **[**[**2**](#_ENREF_2)**]** |
| *Chlamydia^*^* | *psittaci* | UP000014827 | 2871 | 38 | Respiratory psittacosis  (can be life-threatening) | Air- or water-borne, etc. | Obligate intracellular parasitic | 15-60 Days | [**http://www.cfsph.iastate.edu/Factsheets/pdfs/psittacosis.pdf**](http://www.cfsph.iastate.edu/Factsheets/pdfs/psittacosis.pdf) |
| *Hodgkinia* | *cicadicola* | UP000002741 | 169 | 3 | N/A | Cicada-associated | Endosymbiont | N/A | **[**[**3**](#_ENREF_3)**]** |
| *Holospora* | *obtusa* | UP000019112 | 1116 | 30 | N/A | *Paramecium caudatum*-associated | Endosymbiont | N/A | **[**[**4**](#_ENREF_4)**]** |
| *Midichloria* | *mitochondrii* | UP000006639 | 1181 | 43 | N/A | tick-borne | Intramitochondrial symbiont | N/A | **[**[**5**](#_ENREF_5)**]** |
| *Moranella* | *endobia* | UP000000504 | 405 | 18 | N/A | N/A | Symbiotic | N/A | **[**[**6**](#_ENREF_6)**]** |
| *Onion* | *yellows* | UP000002523 | 730 | 11 | Mild-syndrome | Plant/insect-associated | Obligate intracellular parasitic | N/A | **[**[**7**](#_ENREF_7)**]** |
| *Orientia^*^* | *tsutsugamushi* | UP000001565 | 966 | 20 | Scrub typhus | Mite-borne | Obligate intracellular parasitic | N/A | **[**[**8**](#_ENREF_8)**]** |
| *Phytoplasma* | *mali* | UP000002020 | 448 | 15 | Apple proliferation | N/A | Plant parasite | N/A | **[**[**9**](#_ENREF_9)**]** |
| *Rickettsia^*^* | *prowazekii* | UP000002480 | 834 | 42 | Typhus | Lice-borne | Obligate intracellular parasitic | Several months in feces | [**https://www.ncbi.nlm.nih.gov/books/NBK448173/**](https://www.ncbi.nlm.nih.gov/books/NBK448173/) |
| *Sulcia* | *muelleri* | UP000000781 | 227 | 4 | N/A | Insect-associated | Symbiotic | N/A | **[**[**10**](#_ENREF_10)**]** |
| *Ureaplasma*^*^ | *parvum* | UP000000423 | 611 | 14 | Mild diseases | Sexually transmitted | Parasitic | N/A | **[**[**11**](#_ENREF_11)**]** |
| *Zinderia* | *insecticola* | UP000001303 | 206 | 3 | N/A | Spittlebug-associated | Symbiotic | N/A | **[**[**12**](#_ENREF_12)**]** |

^*^Human pathogens.

^#^UPI: UniProt Proteome ID, : Proteome Size, V: Virulence, P: Persistence.

**References**

1. Dumler JS, Choi KS, Garcia-Garcia JC, Barat NS, Scorpio DG, Garyu JW *et al*. Human granulocytic anaplasmosis and Anaplasma phagocytophilum*.* *Emerg Infect Dis* 11(12), 1828-1834 (2005).

2. Van Ham RC, Kamerbeek J, Palacios C, Rausell C, Abascal F, Bastolla U *et al*. Reductive genome evolution in Buchnera aphidicola*.* *Proc Natl Acad Sci U S A* 100(2), 581-586 (2003).

3. Molloy S. Bacterial genetics: A tiny alternative*.* *Nat Rev Microbiol* 7 1 (2009).

4. Hori M, Fujishima M. The endosymbiotic bacterium Holospora obtusa enhances heat-shock gene expression of the host Paramecium caudatum*.* *J Eukaryot Microbiol* 50(4), 293-298 (2003).

5. Sassera D, Beninati T, Bandi C, Bouman EA, Sacchi L, Fabbi M *et al*. 'Candidatus Midichloria mitochondrii', an endosymbiont of the tick Ixodes ricinus with a unique intramitochondrial lifestyle*.* *Int J Syst Evol Microbiol* 56(Pt 11), 2535-2540 (2006).

6. Lopez-Madrigal S, Balmand S, Latorre A, Heddi A, Moya A, Gil R. How does Tremblaya princeps get essential proteins from its nested partner Moranella endobia in the Mealybug Planoccocus citri? *PLoS One* 8(10), e77307 (2013).

7. Neriya Y, Maejima K, Nijo T, Tomomitsu T, Yusa A, Himeno M *et al*. Onion yellow phytoplasma P38 protein plays a role in adhesion to the hosts*.* *FEMS Microbiol Lett* 361(2), 115-122 (2014).

8. Paris DH, Phetsouvanh R, Tanganuchitcharnchai A, Jones M, Jenjaroen K, Vongsouvath M *et al*. Orientia tsutsugamushi in human scrub typhus eschars shows tropism for dendritic cells and monocytes rather than endothelium*.* *PLoS Negl Trop Dis* 6(1), e1466 (2012).

9. Seemuller E, Schneider B. 'Candidatus Phytoplasma mali', 'Candidatus Phytoplasma pyri' and 'Candidatus Phytoplasma prunorum', the causal agents of apple proliferation, pear decline and European stone fruit yellows, respectively*.* *Int J Syst Evol Microbiol* 54(Pt 4), 1217-1226 (2004).

10. Chang HH, Cho ST, Canale MC, Mugford ST, Lopes JR, Hogenhout SA *et al*. Complete Genome Sequence of "Candidatus Sulcia muelleri" ML, an Obligate Nutritional Symbiont of Maize Leafhopper (Dalbulus maidis)*.* *Genome Announc* 3(1), (2015).

11. Kong F, Ma Z, James G, Gordon S, Gilbert GL. Species identification and subtyping of Ureaplasma parvum and Ureaplasma urealyticum using PCR-based assays*.* *J Clin Microbiol* 38(3), 1175-1179 (2000).

12. Mccutcheon JP, Moran NA. Functional convergence in reduced genomes of bacterial symbionts spanning 200 My of evolution*.* *Genome Biol Evol* 2 708-718 (2010).
